# High Internal Phase Pickering Emulsions as Structurally Tunable Bioinks for Extrusion‐Based Printing From 3D Fabrication to Adaptive Multiresponsive Architectures

**DOI:** 10.1111/1541-4337.70579

**Published:** 2026-07-23

**Authors:** Parham Joolaei Ahranjani, Kamine Dehghan, Gergely Kali, Andreas Bernkop‐Schnürch, Giovanna Ferrentino

**Affiliations:** ^1^ Faculty of Agricultural, Environmental and Food Sciences Free University of Bolzano Bolzano Italy; ^2^ Centre for Chemistry and Biomedicine (CCB), Department of Pharmaceutical Technology, Institute of Pharmacy University of Innsbruck Innsbruck Austria

**Keywords:** extrusion‐based printing, high internal phase Pickering emulsions (HIPPEs), interface‐driven design, interfacial jamming, multidimensional printing (3D–6D), stimuli‐responsive architectures

## Abstract

High internal phase Pickering emulsions (HIPPEs) have recently emerged as a versatile class of bioinks for extrusion‐based printing, offering unique opportunities for structural tunability, hierarchical architecture, and functional responsiveness. Stabilized by solid particles at high internal phase fractions (typically ≥74%), HIPPEs exhibit yield stress, pronounced shear thinning, and rapid structural recovery, which are essential for printability and shape fidelity. Unlike conventional hydrogel‐based bioinks that primarily rely on bulk polymer networks, HIPPEs derive their mechanical integrity from particle‐stabilized interfaces and jammed droplet assemblies, enabling decoupled control over mechanics, porosity, and functional loading. This systematic review critically examines the application of HIPPEs as bioinks in extrusion‐based printing, from 3D fabrication toward emerging adaptive multiresponsive architectures. Emphasis is placed on fundamental stabilization mechanisms, particle–interface interactions, formulation parameters governing internal architecture, and rheological properties underpinning extrusion behaviors. Representative case studies are analyzed to elucidate structure–function relationships, encapsulation performance, and functional outcomes in food, biomedical, and bioengineering contexts. Additionally, a further comparison of HIPPE‐based bioinks with conventional hydrogel systems highlights distinct advantages in hierarchical porosity, hydrophobic cargo protection, and responsiveness, while also addressing key challenges related to formulation sensitivity, scalability, reproducibility, and translational validation. Finally, emerging trends in interface‐driven design, multi‐stimuli adaptability, and integration with artificial intelligence and digital fabrication are discussed as future directions for advancing HIPPE‐based bioinks toward intelligent, multifunctional printing platforms.

**Abbreviations**
3ITT: three‐interval thixotropy test
ASN: aminated silica nanoparticle
CA: cinnamaldehyde
C‐ChNW: carboxylated chitin nanowhisker
CG: carrageenan
CIFI: citrus insoluble fiber ingredient
CMC: carboxymethyl cellulose
CN: cellulose nanocrystal (shell component)
CNC: cellulose nanocrystal
CS: chitosan
CUR: curcumin
DCM: dichloromethane
DIW: direct ink writing
DLP: digital light processing
EE: encapsulation efficiency
EGaIn: eutectic gallium–indium alloy
GA: Gum arabic
HAMA: methacrylated hyaluronic acid
H‐CNC: hydrophobically modified cellulose nanocrystal
HIPE: high internal phase emulsions
HIPPE: high internal phase Pickering emulsion
HSPI: heat‐set soy protein isolate
KCF: κ‐carrageenan fraction
KCM: κ‐carrageenan middle‐molecular‐weight fraction
KCO: κ‐carrageenan oligosaccharide fraction
KG: Konjac glucomannan
LAOS: large‐amplitude oscillatory shear
LC: loading capacity
LCST: lower critical solution temperature
MG63: human osteosarcoma MG‐63 cell line
m‐HAp: mesoporous hydroxyapatite
Na_3_Cit: sodium citrate
OP: ovalbumin–pectin
OSA: octenyl succinic anhydride
OTA: oxidized tannic acid
O/W: oil‐in‐water
PCL: poly(ε‐caprolactone)
PEGDA: poly(ethylene glycol) diacrylate
PLA‐GMA: polylactide glycidyl methacrylate
PPI: pea protein isolate
Pr: printability/print fidelity metric
SBP: soybean protein
SC: sodium caseinate
SLA: stereolithography
SPI: soy protein isolate
TA: tannic acid
Ugi‐OA: aldehyde‐modified alginate (Ugi reaction precursor)
W/O: water‐in‐oil
W/O/W: water‐in‐oil‐in‐water
XG: xanthan gum
ZnO: zinc oxide

## 1 Introduction

High internal phase Pickering emulsions (HIPPEs) are colloidal systems characterized by dispersed‐phase volume fractions typically exceeding 74%, at which droplets become densely packed and mechanically jammed (Durgut and Claeyssens 2025). Stabilized by solid particles irreversibly adsorbed at oil–water interfaces, HIPPEs exhibit exceptional resistance to coalescence and structural collapse compared to surfactant‐stabilized emulsions (Bolchini et al. 2026; Joolaei Ahranjani and Ferrentino 2025). The adsorption of particles with intermediate wettability generates robust interfacial films whose mechanical integrity governs the macroscopic behavior of the emulsion (Joolaei Ahranjani et al. 2025; Y. Xu et al. 2025). As a result, HIPPEs behave as soft solids with tunable viscoelastic properties, primarily arising from interfacial particle networks and droplet–droplet confinement rather than from bulk polymer crosslinking (Rehman et al. 2024).

The mechanical response of HIPPEs can be tailored through formulation parameters such as dispersed‐phase fraction, particle concentration and geometry, interfacial wettability, and continuous‐phase composition (Joolaei Ahranjani, Dehghan, et al. 2026; Rehman et al. 2024). By adjusting these variables, HIPPEs can be engineered to display yield stress (*τ*_0_), pronounced shear thinning, and rapid thixotropic recovery. Importantly, these rheological features emerge intrinsically from interfacial jamming and reversible droplet rearrangement, allowing for simultaneous control of mechanical strength, internal porosity, and functional loading (Joolaei Ahranjani, Dehghan, et al. 2026; Liao et al. 2024). The compartmentalized structure of HIPPEs further provides natural microreservoirs for encapsulating lipophilic compounds, bioactives, or phase‐specific additives, while particle‐laden interfaces can regulate diffusion, protection, and release (Ahmadi et al. 2025; Joolaei Ahranjani et al. 2025; Ekrami et al. 2022). Unlike conventional emulsions containing lower dispersed‐phase fractions, HIPPEs exploit droplet crowding above the random close‐packing threshold (internal [dispersed‐phase] volume fraction [*φ*] > 0.74), where droplets become mechanically jammed and collectively contribute to network formation. This jammed microstructure generates solid‐like characteristics, including *τ*_0_ and shape retention, without requiring excessive polymer concentrations or extensive post‐print crosslinking (Joolaei Ahranjani, Dehghan, et al. 2026). The high internal phase fraction also maximizes the volumetric loading capacity (LC) for hydrophobic bioactives, oils, nutraceuticals, and functional ingredients while creating interconnected porous pathways that can be tailored to regulate diffusion, release kinetics, and mass transport. Consequently, the high oil fraction in HIPPE‐based bioinks is not merely a compositional feature but a structural design strategy that simultaneously enables printability, hierarchical architecture formation, and multifunctional cargo delivery (Durgut and Claeyssens 2025; Rehman et al. 2024).

These characteristics have positioned HIPPEs as promising candidates for extrusion‐based printing technologies (Yu, Han, Liu, et al. 2024). Extrusion printing requires inks that undergo reversible flow under high shear within a nozzle and rapidly recover their solid‐like structure after deposition to preserve filament shape and enable multilayer stacking (Ahamdi et al. 2025; Feng et al. 2022; Joolaei Ahranjani et al. 2025). The interfacial origin of HIPPE rheology enables smooth extrusion through shear‐induced droplet rearrangement, followed by rapid re‐jamming of the droplet network upon shear cessation (X. He and Lu 2024; Joolaei Ahranjani and Ferrentino 2026). Unlike conventional hydrogel inks, which often rely on high polymer concentrations or fast crosslinking reactions to maintain shape fidelity, HIPPE‐based inks can achieve printability through physical, interface‐driven mechanisms, reducing dependence on chemical curing and expanding the accessible design space (Wenger et al. 2020).

In extrusion‐based printing, the ability to decouple printability from bulk polymer content is particularly advantageous for fabricating architectures with high porosity, hierarchical internal structures, and efficient incorporation of hydrophobic payloads (X. Li et al. 2023a). HIPPE‐based inks have therefore been explored in a growing range of printing applications, including food structuring, nutraceutical delivery, tissue‐mimetic scaffolds, and functional soft materials (Dehghan et al. 2025; Q. Zhao, Fan, et al. 2023). Moreover, the modularity of HIPPE formulations allows spatial and temporal adjustment of rheological and functional properties, supporting not only static three‐dimensional constructs but also more advanced printing paradigms (Y. Zhang and Yu 2023).

Recent studies have extended HIPPE‐enabled extrusion printing beyond conventional 3D fabrication to time‐dependent and stimuli‐responsive systems, as well as to spatially heterogeneous and functionally graded constructs (Ghazal et al. 2023; Shahbazi, Jäger, et al. 2024). In the broader additive manufacturing literature, 4D printing is generally associated with time‐dependent transformations under external stimuli, whereas terminology describing higher levels of functional complexity remains less standardized and is often used inconsistently across disciplines (Ghazal et al. 2023; Han et al. 2023). Accordingly, this review focuses on emerging adaptive multiresponsive architectures capable of modulating properties such as shape, permeability, release behavior, conductivity, or mechanical response under one or more external stimuli, rather than adopting non‐uniform dimensional classifications.

Previous investigations have summarized specific aspects of Pickering emulsions, food‐grade HIPPEs, or extrusion‐based printing systems, including stabilization mechanisms, rheological behavior, and general 3D printing applications (X. He and Lu 2024; Rehman et al. 2024; Wu et al. 2022). Other studies have discussed multidimensional food printing and stimuli‐responsive architectures more broadly without specifically focusing on HIPPE‐based bioinks or interfacial rheology‐governed printability (Ghazal et al. 2023; Han et al. 2023). In contrast, the present review integrates HIPPE stabilization mechanisms, interfacial particle assembly, rheology–printability relationships, multidimensional printing paradigms from 3D to emerging 6D architectures, and translational considerations within a unified interface‐driven framework. Particular emphasis is placed on the mechanistic coupling between interfacial organization, droplet jamming, nonlinear rheology, and adaptive structural functionality across food, biomedical, and functional material applications.

In this systematic review, we critically examine HIPPEs as structurally tunable bioinks for extrusion‐based multidimensional printing. The review is organized progressively from the physicochemical foundations governing HIPPE formation and stabilization (Section 3) to formulation–rheology–printability relationships relevant to bioink design (Section 4), followed by their application in multidimensional printing platforms (Section 5) and emerging stimuli‐responsive architectures (Section 6). Finally, current limitations, translational challenges, and future research directions are critically evaluated (Sections 7 and 8). This framework links interfacial science, printing performance, and functional adaptation within a unified design perspective.

## 2 Methodology

### 2.1 Search Strategy

This systematic review was conducted following the Preferred Reporting Items for Systematic reviews and Meta‐Analyses (PRISMA) guidelines (Moher et al. 2009). Literature searches were performed up to December 2025 using PubMed, Scopus, and Web of Science to capture studies at the intersection of HIPPEs and extrusion‐based or multidimensional printing technologies. Search terms encompassed HIPPE‐related concepts (e.g., Pickering stabilization, particle‐stabilized emulsions, and interfacial engineering) together with additive manufacturing and multidimensional printing terminology (e.g., extrusion printing, direct ink writing [DIW], 3D–6D printing, and stimuli‐responsive fabrication). Detailed search strategies are provided in the Supporting Information. A total of 668 records were initially identified across all databases (Supporting information).

### 2.2 Study Selection

After removal of duplicates, 352 unique records were screened based on titles and abstracts. Studies were considered eligible when they investigated particle‐stabilized high internal phase emulsions (HIPEs) and reported characteristics relevant to extrusion‐based printing, including rheological behavior, interfacial properties, structural stability, printability, or multidimensional printing performance. Conference abstracts, patents, editorials, non‐peer‐reviewed publications, and studies unrelated to extrusion‐based fabrication were excluded. Following full‐text assessment, 143 studies were retained for qualitative synthesis and critical evaluation.

### 2.3 Data Extraction and Synthesis Methods

**FIGURE 1:**
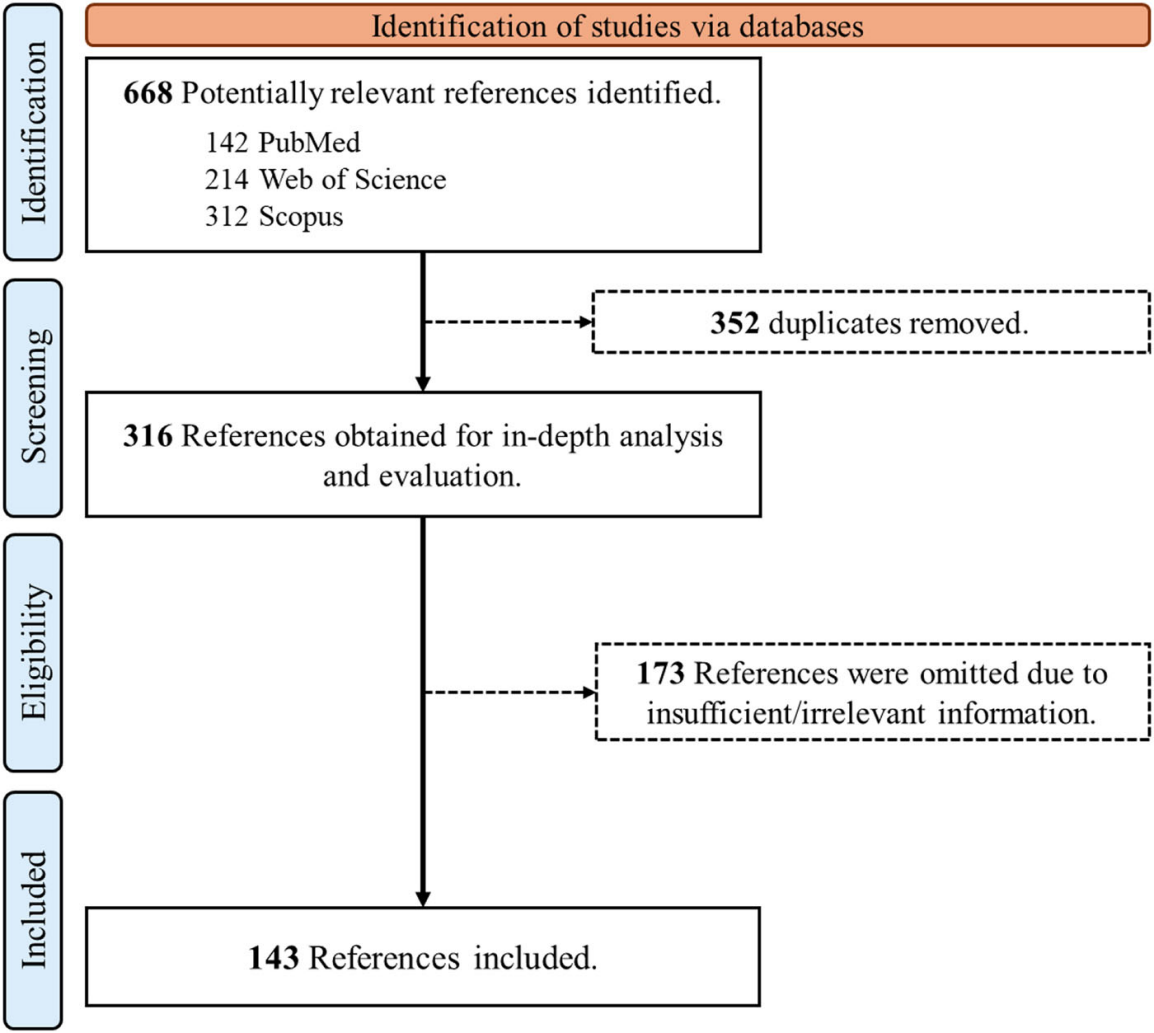
PRISMA flow diagram illustrating the identification, screening, eligibility assessment, and final inclusion of studies in this systematic review on bioinks for extrusion‐based and multidimensional printing.

Data were extracted on formulation composition, stabilization mechanisms, interfacial characteristics, rheological properties, printing parameters, and application‐specific outcomes. Owing to substantial heterogeneity among formulations, characterization methods, and printing platforms, quantitative meta‐analysis was not feasible. Therefore, a qualitative comparative approach was adopted, grouping studies according to stabilization strategy, particle type, and printing dimensionality to identify recurring formulation–rheology–printability relationships, emerging design principles, and current knowledge gaps. The study selection process is summarized in the PRISMA flow diagram (Figure 1).

## 3 Fundamentals of HIPPEs

### 3.1 Composition and Stabilization Mechanisms

**FIGURE 2:**
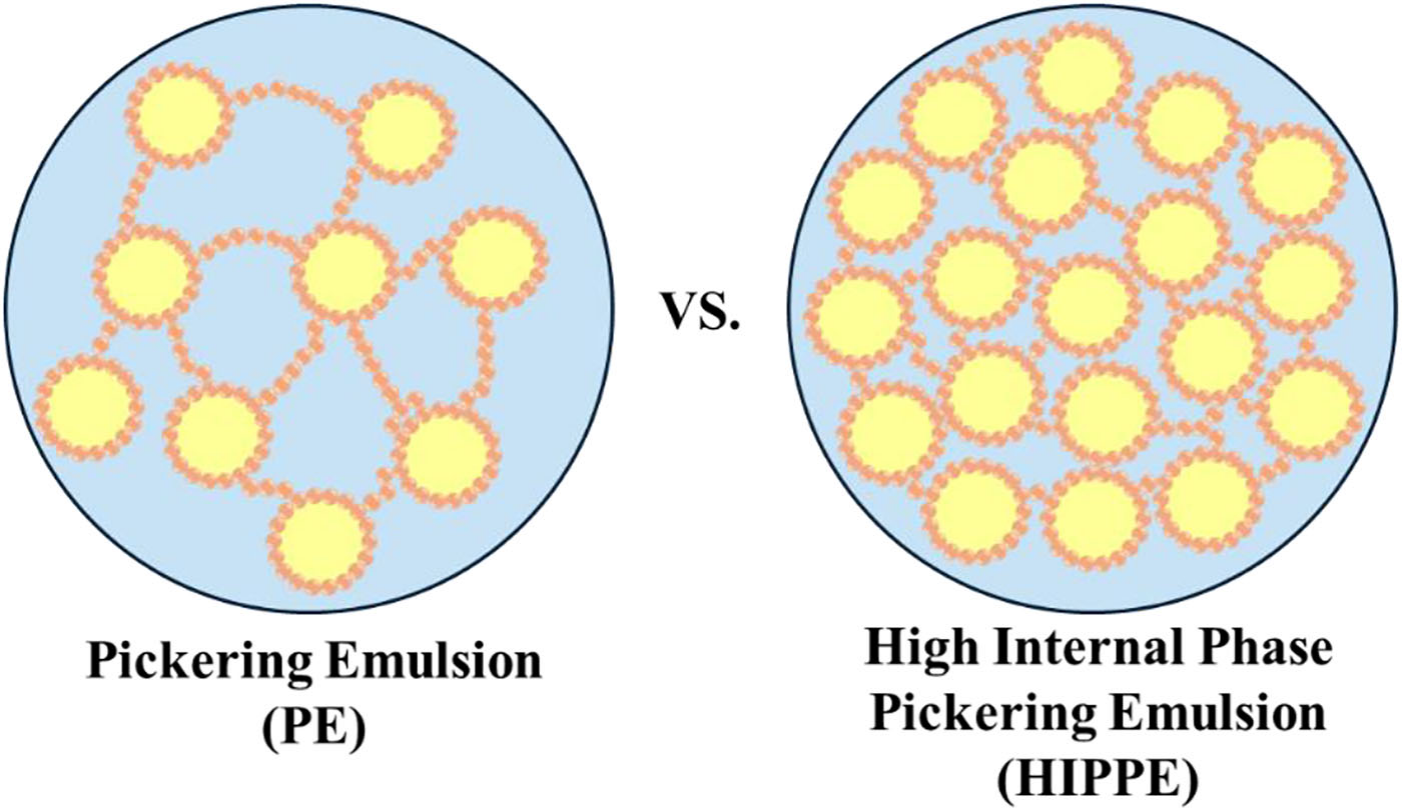
Schematic comparison of Pickering emulsion networks (left) versus densely packed HIPPE (right) within a continuous matrix.

HIPPEs are characterized by dispersed‐phase volume fractions exceeding ∼0.74 (Figure 2), where droplet jamming imposes solid‐like behavior and stabilization depends on irreversible particle adsorption rather than surfactant dynamics (Wenger et al. 2020). Across HIPPE‐based bioinks reported in Table 1, stable systems consistently require particle loadings above system‐specific thresholds to achieve sufficient interfacial coverage. For example, β‐cyclodextrin (β‐CD)‐stabilized HIPPEs form self‐supporting printed filaments only at ≥1.2–1.5 wt% β‐CD, corresponding to *τ*_0_ of ∼200–250 Pa, whereas formulations ≤0.9 wt% collapse after deposition despite identical oil fractions (X. Li, Fan, et al. 2023). Similarly, ovalbumin–pectin (OP) HIPPEs exhibit a sharp transition in stability when complexation reduces droplet size from around 35 µm to approximately 12 µm and increases viscosity recovery from about 67% to over 95%, enabling stable extrusion (Q.‐H. Li, Li, et al. 2023). From a mechanistic perspective, HIPPE stability originates from the irreversible adsorption of particles at the oil–water interface, where the particle detachment energy is several orders of magnitude greater than thermal energy (kBT), generating robust interfacial barriers against droplet coalescence and Ostwald ripening (Binks 2002; Durgut and Claeyssens 2025).

**TABLE 1:** Interfacial stabilization strategies, rheological properties, and printing performance of HIPPE‐based bioinks reported for extrusion‐based and multidimensional printing.

HIPPE system	Key principle	Composition	Interfacial mechanisms	Fabrication mechanisms	Rheological profile	Stability	Application	Advantages	Limitations	Reference
W/O PCL–DCM/water–PVA HIPE	Nanoclay Pickering stabilization for 3D printing	PCL 5–7 wt% in DCM; water *φ* = 0.75; PVA 0.1 wt%; Cloisite 30B 0.1–2 wt% (stable ≥1 wt%); ibuprofen 2.5 wt%	Clay platelets (∼200 nm) adsorb at DCM–water interface; PVA ↑ viscosity ∼5×; stable HIPE only when clay ≥1 wt%	Stirring 500 rpm, 30 min; printed at 25°C; nozzle Ø = 0.64 mm; layer height 0.30 mm; speed 15 mm/s	1000× shear thinning (0.1 → 100 s^−1^); *G*ʹ > *G*ʺ; rapid recovery; *η* ≈ 3370 Pa·s at 0.1 s^−1^ (with PVA)	<0.5 wt% clay → unstable (<12 h); ≥1 wt% → stable 24 h to 1 week; droplet size 3–25 µm	Bone scaffolds; ibuprofen delivery; MG63 growth	Porosity 93.8%–97.7%; density 0.034–0.096 g/cm^3^; drug loading 97%–99%; release 55%–75% (24 h) → 95%–99% (130 h); strong biomineralization (Ca/P > 1.67)	DCM solvent; clay >2 wt% lowers MG63 viability; *φ* = 0.75 may cause coalescence; slow PCL degradation	Ghosh et al. 2023
PPI/KG–soybean oil o/w HIPPE	Protein–KG complex stabilization for printability	PPI 5% + KG 0%–0.5%; soybean oil 75%; curcumin 0.1%; pH 3–9	*ζ*‐potential: −32.9 → −24.1 mV (0%–0.2% KG), droplet size 7.90 → 6.47 µm; complex size 3.48 → 9.93 µm	Homogenization 12,000 rpm, 3 min; printed with 0.75 mm nozzle at 25 mm/s	*τ*_0_ 199.5 → 896.8 Pa; *n* = 0.37–0.42; K = 70.3–195.6 Pa·s*^n^*; *G*ʹ > *G*ʺ	Stable ≥14 days at 4°C when KG ≥0.3%; unstable at pH 3–5	3D‐printed curcumin gels	Encapsulation efficiency 64.4% → 83.54%; improved shape fidelity KG ≥0.3%	Printing collapse at KG ≤0.1%; no *G*ʹ/*G*ʺ values reported	Z. Liu et al. 2024
OSA–potato starch (CF03/CM1120) + CIFI o/w HIPE	OSA–starch/CIFI hybrid stabilization tuned by DS	Starch 4% (DS: CF03 = 0.48%, CM1120 = 2.64%); CIFI 0%–0.8%; oil 75%	Droplet size (CF03) 16 → 7 µm; (CM1120) 7 → 3 µm; LF‐NMR T_2_ shift indicates thicker interfacial layer	Homogenization 12,000 rpm, 2 min; nozzle 0.84 mm; speed 15 mm/s; 25°C	*τ*_0_ CF03: 10 → 131 Pa; CM1120: 33 → 218 Pa (CIFI 0% → 0.8%); strong shear thinning; viscosity recovery max 80.3%	Side‐area loss 96 h: CF03 47% → 19%; CM1120 23% → 13%; thermal retention at 50°C: 96%–98%	3D‐printed edible shapes (30 × 30 × 8 mm)	Highest accuracy 97.7%; SFI 0.91; droplet size min 3 µm; improved hardness/adhesiveness	Pure starch fails to print; low CIFI (<0.2%) unstable; poor pH (<4) tolerance	Wei et al. 2025
β‐Cyclodextrin (β‐CD)–sunflower oil O/W HIPE	β‐CD inclusion complex particles stabilize interface; pH‐ and salt‐responsive assembly	Oil 75%; β‐CD 0.3–1.5 wt%; pH 3–7; NaCl 0–400 mM; CUR 0.1% (for 4D); NaHCO_3_ 0%–1.5%	Droplet size decreases as β‐CD ↑ (0.6%: large; 1.2%–1.5%: tightly packed); *ζ*‐potential increases with β‐CD; interfacial tension decreases with β‐CD and ↓pH; ionic strength 25 mM sharply reduces *D*_4,3_	Ultra‐Turrax 12,000 rpm, 3 min; printing nozzle 0.8 mm; speed 20 mm/s; retraction 50 mm/s; RT	Shear‐thinning; viscosity ↑ with β‐CD and ↓ pH; 3ITT recovery improved for β‐CD ≥1.2%; *τ*_0_: pH 3 = 244 Pa → pH 7 = 34.8 Pa; *G*ʹ > *G*ʺ across all conditions	Stable gel‐like inks at β‐CD ≥0.6%; best structural support at 1.2%–1.5%; pH 3–5: highest viscosity, lowest strain; NaCl 25–400 mM improves packing	3D printing: grids, hollow cuboids, butterflies, lotus, octopus; 4D color change with CUR/NaHCO_3_	β‐CD ≥1.2% gives good fidelity; optimal 3D: β‐CD 1.2%–1.5% and pH ≤4; 4D: tunable color (yellow → red) by heat/time/NaHCO_3_	β‐CD ≤0.9% collapses; pH ≥6 causes layer fusion/poor recovery; high heat (>105°C) breaks emulsion; 1.2% β‐CD insufficient for complex shapes	X. Li, Fan, et al. 2023
W/O HIPE (polyHIPE scaffold + enzyme‐laden hydrogel)	Cure‐on‐dispense UV polymerization; hydrogel‐filled voids for enzyme entrapment	Organic phase: EHA/IBOA/TMPTA (2.3:4.2:1), surfactant Pluronic L‐121 = 6%–12% w/w; aqueous phase: AA + PEG‐DA700 (AA:PEG‐DA = 10:1), LAP 1 mg/mL, β‐galactosidase 40 U/mL; aqueous phase fraction = 80%–90% v/v	Void size ↓ as surfactant ↑ (6% → 12%); 14% monomer → hydrogel spheres inside voids; high surfactant = smaller voids = better openness	Emulsification: add aqueous 1.25 mL/min; stirring 600 → 800 → 1000 rpm; 5 min final; printing nozzles: 110, 250, and 840 µm; layer height 100–300 µm; UV 3.5–13 mW/cm^2^; post‐cure 25 mW/cm^2^ × 2 min	*τ*_0_: surfactant 6% → 12% = 10× increase; aqueous 80% → 90% = 3.5× increase; monomer 0%–14% = 123–148 Pa (weak trend); *G*ʹ > *G*ʺ; gel‐like	Equilibration time: nozzle 110 µm = 6.6 ± 3.1 min → 840 µm = 52.0 ± 0.9 min; enzyme leaching: ∼0.14%–2.3% (most), 110 µm = 8%	3D‐printed polyHIPE enzymatic reactors, grids, gyroids, hollow cylinders (8 × 2.4 mm)	Printability excellent; complex overhangs; specific activity ↑ 5× when monomer 0 → 7%; nozzle 110 µm → 4× activity vs. 840 µm	Hydrogel shrinkage during SEM; 12% surfactant → scaffold collapse (drying); enzyme leaching during wash; diffusion limitations	Wenger et al. 2020
Casein–soybean/shiitake oil O/W HIPE	Phenolic–casein interactions form crosslinked droplet networks	5% casein; oil 80% phase (shiitake oil 0%–60% w/w blended with soybean oil); pH 6	Interfacial tension ↓ 17.93 → 7.74 mN/m (0% to 60% shiitake); ACP ↑ 53.75 → 89.66%; stronger hydrophobic aggregation with higher phenolics	Homogenization 8000 rpm, 1 min; nozzle 1 mm; print speed 5.2 mm^3^/s; RT	*τ*_0_ 81.8 → 309 Pa; viscosity 409–1.74 Pa·s → 1762–2.93 Pa·s; *G*ʹ > *G*ʺ; 3ITT recovery 95%–114%; stronger plasticity at 60% (rectangular LAOS loops)	Good layer retention for ≥35%–60% shiitake; low‐oil samples (0%–20%) spread and merge; cylinder collapse except at 60%	3D Chinese knots and cylinders (20 layers)	Best print fidelity at 50%–60% shiitake; strong crosslinked networks; greatly enhanced elasticity	0%–35% prints collapse; low recovery at 60% (95%); broad droplet distribution (10–100 µm) at high shiitake	A.‐Q. Bi et al. 2024
SNC‐P6/SNP‐P6 Pickering O/W HIPE for 4D printing	Hydroxybutyl‐modified starch nanocrystals/nanoparticles as thermoresponsive Pickering stabilizers	75% corn oil; 25% aqueous phase with SNC‐P6 or SNP‐P6 (0.5–1 wt% range inferred); internal phase *φ* = 0.75	Modified SNC/SNP show ↑ hydrophobicity (contact angle SNC‐P6 = 70.4°, SNP‐P6 = 63.0°); strong droplet flocculation; small mean droplet sizes (<20–30 µm) with aggregated clusters; high interfacial adsorption	Rotor‐stator 12,000 rpm, 2 min → ultrasound 5 min (20 kHz, 60%, 450 W) → printable gel ink	*G*ʹ > *G*ʺ across frequency; strong gel‐like behavior; LVR critical strain >10%; shear thinning; high viscosity; thixotropic recovery E‐SNC‐M ≈ 87%, E‐SNP‐M ≈ 82%; small harmonic nonlinearity (Q0 reduced by ∼2 orders)	Physically stable ≥30 days; no creaming; ΔBS ∼0; stable gel network; printed scaffolds remain intact	4D‐printed macroporous scaffolds; thermoresponsive LCST trigger around 37°C	High print fidelity (Pr: 0.93 ± 0.12 for SNP‐M; 0.98 ± 0.08 for SNC‐M); hierarchical pores; LCST‐responsive swelling; strong mechanical integrity	Requires plasma grafting; unmodified SNC/SNP fail to stabilize HIPE; flocculated structure sensitive to formulation	Shahbazi, Jäger, et al. 2024
Gel‐in‐gel W/O HIPE (SP + beeswax + potato starch)	Biphasic network stabilization for reduced‐fat 3D printing	SP 0.5–3 wt%; beeswax 1–4 wt%; water 70%–85% (*φ*); gel‐in‐gel *φ* = 0.80; starch hydrogel 1–2 wt%	SP lowers *γ* from 19.50 → 6.83 mN/m; droplet size decreases 8.60 → 3.12 µm; beeswax crystal spacing shrinks 4.09 → 3.49 Å	Oil phase melted at 70°C; premix at 1200 rpm; homogenization 12,000 rpm, 5 min; printing: 25°C, 0.8 mm nozzle, 15 mm/s	*G*ʹ > *G*ʺ in all HIPEs; *G*ʹ increases with SP and starch; viscosity ↑; shear thinning; thixotropic recovery: 51.3% (O‐HIPE) → 82.3% (2% starch); yield resistance ↑	O‐HIPE collapses at 45 days; gel‐in‐gel stable >60 days; centrifugation (7000 rpm, 30 min) → no leakage (1%–2% starch)	3D printing of hollow structures (“ladybug” and “pumpkin”); complete shape fidelity only with 2% starch	Fat reduced to 20%; droplet ↓ to 3.23 µm; strong viscoelasticity; high recovery; self‐supporting printing; fine structural resolution	Phase inversion at Φ = 0.85 or starch = 3%; excess beeswax (4%) causes separation; limited water fraction window	M. Wang, Zhou, et al. 2024
PPC–peanut oil HIPE	Natural bead‐on‐a‐string PPC nanostructures (≈300 nm beads, 50 nm strings) as amphiphilic stabilizers	PPC 3 wt% (protein 75.8%, polysaccharide 11.6%); peanut oil; *φ* = 80%; pH 7	TPCA ≈ 92.1° at pH 7; coherent protein–polysaccharide interfacial films; interwoven networks between droplets; zeta potential ≈ −35 mV (pH 7–12)	Homogenization at 20,000 rpm, 60 s	*G*ʹ >> *G*ʺ; *G*ʹ peak at pH 7; *τ*_0_ ≈ 77.13 Pa (pH 7, PPC 3%); viscosity highest at pH 7; smallest droplet ≈ 12.09 µm at 3% PPC	Heat‐stable up to 90°C × 20 min; storage‐stable 30 days with negligible size growth (≈17 → 19 µm)	3D‐printed self‐standing structures (1.2 mm nozzle; 900 mm/min; flow 0.149 cm^3^/s)	Forms HIPEs up to Φ 86%; excellent printability, elasticity, high structural resolution; natural, solvent‐free	No gelation at Φ ≥87%; unstable at pH 3–5; trypsin digestion eliminates stabilization (protein essential)	Zong et al. 2022
ACC‐copolymer/DCM–ZnO W/O HIPE	Keto–hydrazide + Zn^2^^+^ ionic crosslinking enabling ambient‐temperature curing and printability	ACC copolymer (DAAM 0–9.5 wt%); ZnO 1–5 w/v%; DCM 20 vol%; TEA 0.04 mmol; ADH 60 mol% (vs. DAAM); *φ* = 0.80; droplet size 5.1–13.5 µm	ZnO (∼30 nm) forms Pickering layer + ionic crosslinking with –COOH; keto–hydrazide crosslinking upon evaporation; higher ZnO ↓ droplet size (10.6 → 5.1 µm)	Homogenization 10,000 rpm, 2.5 min (lab); scale‐up 1500 rpm, 12 min; extrusion/3D‐printing via nozzle/cake bag	Strong shear thinning; *η* decreases 0.1 → 100 s^−1^; *G*ʹ > *G*ʺ across 0.1–100 rad/s; *G*ʹ >100 Pa; higher copolymer or ZnO ↑ viscosity	Stable during 7‐day ambient curing; shrinkage ≤ 8.6% (ZnO, Al_2_O_3_); gel content 23.3%–65.5%; density 46–114 kg/m^3^; porosity 90.9%–95.7%	3D‐printed porous scaffolds, coatings; large‐area putty‐like application	Ambient‐curing; no monomer residue; tunable pore size (3.8–13.5 µm); high porosity; mechanical modulus 0.6–1.9 MPa; scalable (20×)	Requires DCM; high ZnO → nanoparticle accumulation; curing 3–7 days; pore wall thickening at high copolymer	H. Jiang et al. 2024
Pea‐protein fibril (PPF)–sunflower oil HIPPE gel	One‐dimensional amyloid‐like PPFs forming strong interfacial Pickering layers and 3D gel networks	PPF 1–5 wt%; oil 70 wt%; pH 7; ionic strength 10 mM; incubation at 85°C for 4 h to form fibrils	PPFs adsorb at O/W interface forming rigid elastic shells; droplet size ↓ from 32.6 → 12.4 µm (PPF 1% → 5%); *ζ*‐potential ≈ −38 mV; strong flocculated network	Homogenization 10,000 rpm, 2 min; incubation 4°C × 12 h; 3D printing via extrusion, nozzle 0.8 mm, speed 20 mm/s	*G*ʹ > *G*ʺ; *G*ʹ increases from 1.2 → 4.8 kPa (PPF 1% → 5%); *τ*_0_ 62 → 185 Pa; strong shear thinning; thixotropic recovery ∼85%	Stable for ≥30 days at 4°C; negligible creaming; thermal stability up to 90°C (droplet coalescence only above 95°C); resistant to pH 5–9	3D‐printed self‐supporting grids and cylinders; high‐resolution constructive patterns	Excellent self‐supporting ability; tunable strength; clean‐label protein stabilizer; small droplet size; no surfactants required	Requires high PPF (>3%) for printability; fibril formation slow (4 h at 85°C); sensitive to proteolysis	Shahbazi et al. 2021
H‐CNC–stabilized O/W HIPPE (cyclohexane/canola oil/dodecane)	Hydrophobic interaction–driven stabilization using silylated CNC; aqueous‐condition‐tolerant HIPPE for 3D printing	CNC hydrophobization 2.3%–11.5%; H2‐CNC typically 0.06–2 wt% (aq. base), ≈0.06–0.3 wt% (emulsion); oil fraction 66%–83%; pH 3–12; NaCl 0–1 M	Hydrophobic attraction between grafted C8 chains; adsorption of H‐CNC at interface; reduced *γ* (interfacial tension) vs. CNC; droplet sizes vary 5–180 µm (H5‐CNC bimodal); compact cellulose interfacial film; droplet flocculation	One‐step emulsification: 9000 rpm, 1 min (T25 Ultra‐Turrax); inks 3D‐printed using BIOX; nozzle 200–840 µm; speed 10–20 mm/s; pressure 5–40 kPa	Strong shear thinning: *η* decreases 10^3 ^→ 10^1^ Pa·s (0.01 → 100 s^−1^); *G*ʹ > *G*ʺ; *G*ʹ increases with salt (max at 1 M), decreases with ↑pH; yield‐strain softening at *γ* >10%; rapid thixotropic recovery (≈80%–90%)	Stable under pH 3–12, NaCl 0–1 M, stabilizer ≥0.06 wt%; oil *φ* = 66%–83%; self‐supporting; no coalescence for days; tolerant to elevated temperature; maintains double‐emulsion structure during heating	3D printing of porous scaffolds; thermal‐insulation plates; thermoresponsive hydrogels; methanol‐responsive hydrogels; conductive force‐sensing hydrogels	Extremely low stabilizer use (0.06 wt%); broad aqueous tolerance; tunable droplet size; excellent printability; high resolution (492 ± 52 µm with 27G nozzle); porous structures tunable by pH/salt; high sensitivity as sensor (0.464 kPa^−1^)	H5‐CNC forms large aggregates → bimodal droplets; edible oils decrease resolution due to impurities; printed objects slightly oversized vs. CAD due to extrusion swelling; bottom‐layer compression during printing	X. He et al. 2022
C‐ChNW–paraffin oil O/W HIPE	Carboxylated chitin nanowhiskers (APS oxidation) form 3D interfacial network	C‐ChNWs 1.5–10 wt%; optimal = 7.5 wt%; oil *φ* = 0.5–0.8; pH 9 for *φ* = 0.8	Rod‐like nanowhiskers *L* ≈ 255 nm, *W* ≈ 29 nm; *ζ*‐potential −48.9 mV; droplets fully encased by whisker network (Cryo‐SEM); steric hindrance + electrostatic repulsion	High‐speed shearing 10,000 rpm, 3 min; high‐pressure homogenization (three cycles, 10,000 Pa); *φ* = 0.8 produced by sequential shearing at 15,000 rpm	*G*ʹ < *G*ʺ at ≤7.5 wt% suspension; at 7.5–10 wt% → gel transition; emulsions: *G*ʹ > *G*ʺ (solid‐like); strong shear thinning; viscosity ↓ with shear rate	*φ* = 0.5: separation unless C‐ChNW ≥3 wt%; *φ* = 0.8 with 7.5 wt% C‐ChNWs: no separation for 90 days; stable self‐supporting gel	3D‐printed rings and concentric disks	Strong structure at *φ* ≥0.7; TPA gel strength increases 89.26 → 186.50 g·cm (*φ* = 0.7 → 0.8); good print fidelity; clean‐label nanobiopolymer	Requires high stabilizer content (7.5 wt%); emulsions <3 wt% C‐ChNW unstable; sensitive to shear damage at high shear rates	Shuang et al. 2024
Egg‐yolk/CMC HIPPE (depletion attraction)	CMC acts as nonadsorbing depletant, inducing droplet clustering and particulate‐gel HIPPE	Yolk/CMC mass ratios: 20:0, 20:2.5, 20:5, 20:7.5, 20:10; oil *φ* = 0.8; β‐carotene 0.1% w/w; pH 6	Droplets shrink 34.6 → 8.9 µm (YC‐0 → YC‐7.5); depletion force forms tight droplet clusters; yolk particles adsorb at interface; interfacial tension decreases after 400–600 s	Ultrasonication 300 W, 10 min; homogenization 15,000 rpm, 60 s; printing: nozzle 0.84 mm, speed 25 mm/s, layer 0.6 mm, 25°C	Shear viscosity at 0.1 s^−1^: 99.09 → 768.90 Pa·s (YC‐0 → YC‐10); *G*ʹ > *G*ʺ; *τ*_0_ 24.21 → 90.83 Pa; 3ITT recovery: viscosity restored at 1 s^−1^; strong shear thinning *n* < 0.3	Stable self‐supporting gels for YC‐5–YC‐10; YC‐7.5 strongest structural recovery; YC‐10 excessive viscosity and slight destabilization; no droplet rupture during extrusion	3D‐printed cylinders, cats, dolphins; YC‐7.5 best fidelity (intact ears/fins)	Optimal swallowability for elderly (IDDSI level 4); smallest droplets (8.9 µm); FFA release ↑; β‐carotene bioaccessibility 13.24% → 29.98%; smooth extrusion and shape retention	YC‐0 unstable; YC‐2.5 shows bridging flocculation (no depletion); YC‐10 over‐viscous with reduced digestion rate; high viscosity may limit flow in some printers	Hou et al. 2024
Oxidized SPI–soybean oil O/W HIPE	AAPH oxidation (0–15 mmol/L) improves interfacial adsorption, viscosity, and printability	SPI oxidized at 0, 0.25, 0.5, 1, 5, 15 mmol/L; oil fraction *φ* = 0.75; β‐carotene added (∼0.1%)	Droplet size 34.6 → 28.0 → 17.3 → 8.9 → 11.3 µm (SPI → SPI‐10); adsorbed protein %: SPI ≈ 64%, SPI‐1 ≈ 75%; *ζ*‐potential becomes more negative up to SPI‐1	Ultrasonication 300 W × 10 min → homogenization 15,000 rpm, 60 s; printing: nozzle 0.84 mm, speed 25 mm/s, layer height 0.6 mm, 25°C	Apparent viscosity (0.1 s^−1^): 99.09, 159.34, 263.58, 440.76, 548.17, and 768.90 Pa·s; *τ*_0_: 24.21, 38.92, 62.33, 160.80, 132.44, and 90.83 Pa; consistency *K*: 20.94 → 157.43 mPa·s*^n^*; Flow index *n*: 0.29–0.31; *G*ʹ > *G*ʺ for all; 3ITT recovery >70% for SPI‐1	SPI‐1: stable uniform gel; SPI‐0 unstable; SPI‐5 and SPI‐15 show aggregation; no oil leakage in SPI‐1; consistent droplet morphology after extrusion	3D‐printed cubes and animal shapes; SPI‐1 yields sharpest edges and highest structural fidelity	Best formulation at 1 mmol/L AAPH: smallest droplets (8.9 µm), highest viscosity (440 Pa·s), highest *τ*_0_ (160.8 Pa), highest stability and printability	Over‐oxidized samples (5–15 mmol/L) show weaker gels, reduced *τ*_0_, lower viscosity recovery, and structure collapse	Yuan et al. 2024
OSA starch–citrus fiber HIPE (o/w)	OSA starch emulsification + citrus‐fiber–reinforced network tuning for printability	Sunflower oil 75 wt%; water 22.7 wt%; OSA starch 2 wt%; citrus fiber 0–0.4 wt%; optimal = 0.3 wt%	OSA starch forms interfacial particle network (CLSM; Figure 2); citrus fiber fills inter‐droplet spaces → ↑gel strength; steric hindrance prevents coalescence; higher CF → tighter network	High‐speed shearing 23,000 rpm, 2 min at 25°C; printing nozzle 0.5 mm, speed 25 mm/s, layer 0.5 mm; syringe diameter 3 cm	Shear‐thinning; apparent viscosity ↑ with CF (0.4% highest). At 1 Hz: *G*ʹ ≈ 820 Pa, *G*ʺ ≈ 76 Pa (0.3% CF); LAOS: elastic → viscous transition at 10%–100% strain	Shape accuracy (Table 1): at 0 min: 99.23% (0.3% CF), 97.27% (0.4%), 98.13% (0.2%); stability ≥95% up to 180 min for 0.3%–0.4% CF	3D‐printed elderly food shapes (bird, giraffe, leaf, butterfly, cylinder)	Smooth extrudability; accuracy >95%; hardness 19.18 g, gumminess 0.68 g, springiness 0.58 at 0.3% CF; tribology close to mayonnaise (*μ* < 0.05 at 10 mm/s)	Too low CF (0%–0.2%): weak network, linear defects; too high CF (0.4%): swelling and minor distortion; viscosity may hinder flow	F. Yang et al. 2024
MP–Trehalose O/W HIPE	Trehelase–protein synergy improves interfacial adsorption, freeze–thaw stability, and 3D printability	MP 2% (w/v); Tre 0%–20%; corn oil 80% (*φ* = 0.8); optimal = 10% Tre	AP% 73.67 → 85.22% (0% → 10% Tre); interfacial tension: Tre 20.93 → 12.61 mN/m, MP‐10T 6.89 mN/m; Kdiff 0.125–0.194 mN·m^−1^·s^−0.5^ (max at 10%); *K*_p_ 2.23 × 10^4^ → 3.44 × 10^4^ s^−1^; *K*_r_ 8.63 × 10^4^ → 10.22 × 10^4^ s^−1^	Homogenization 15,000 rpm, 2 min; nozzle 1.2 mm; printing speed 30 mm/s; layer height 1.2 mm	Shear‐thinning; viscosity (0.1 s^−1^): MP ∼1200 Pa·s, MP‐10T ↑ highest; *G*ʹ >> *G*ʺ; 3ITT recovery ∼93% (10% Tre); thermal sweep: *G*ʹ increases 20°C–80°C–20°C; smaller droplets improve moduli	Particle size (PBS): 67.61 → 25.09 µm (0% → 10% Tre); after F–T5: MP‐10T 25.09 → 54.41 µm; FD and CD lowest at 10% Tre; freezing point −14.2°C → −21.1°C; MP control fails after F–T1, MP‐10T stable after F–T5	3D‐printed cylinders, Chinese character “Hua,” snowflake model; PA% 84.26%–92.59% (10% Tre highest)	Best F–T stability, smallest droplets, highest adsorption, strongest *G*ʹ, best structure fidelity; line integrity >90%	Overdose Tre 15%–20% → MP competition, larger aggregates, poor extrusion, lower PA%, weakened gel	Hong et al. 2025
OP–soybean oil O/W HIPE	Protein–polysaccharide electrostatic complex tuning (wettability‐controlled printability)	Ovalbumin:pectin = 1:1 (4 g/L each), pH 4.0; oil = 75%–79%, optimized 77%; OP 0.70%–1.15%, optimal 1.0%	BPB binding: 502 → 1418 µg/g (WA → OP); contact angle: 52.4° → 69.9° (WA → OP); optimized OP: BPB 2710 µg/g, *θ* = 72.2° (pH 3.5), 90.2° (50°C); LF‐NMR: OP highest T22 → densest droplet network	Pre‐emulsion: 14,000 rpm, 5 min; printing: nozzle 0.84 mm, rate 7 mm/s, pressure 7 kPa, layer 0.8 mm, fill density 90%, RT	Shear‐thinning; OP highest viscosity and *G*ʹ; viscosity sequence: pH 3.5 > 3.0 > 4.0 > 5.0; at 3ITT: viscosity recovery 96.18% at OP 1.0%, lowest 67.27% at 1.15%; *G*ʹ >> *G*ʺ except WA (*G*ʹ ≈ *G*ʺ)	Stable self‐supporting at *φ* = 77%; *φ* = 75% → lowest viscosity + 50% recovery; *φ* = 79% → lowest shear stress, unstable; OP‐stabilized HIPE retains shape for complex geometries	3D‐printed pyramid, cube, flowerpot, hollow cylinder; optimized OP prints hollow cylinder and flowerpot without sagging	Highest wettability + compact network; best viscosity recovery (96%); shape fidelity; stable complex structures	WA, GA, and OA show sagging, deformation, water leakage; OP ink collapses for heavy overhangs (anchor tip)	Q.‐H. Li, Li, et al. 2023
SBP‐NH_2_/TA NPs–CA HIPE (o/w)	Interfacial conjugation (Schiff base + H‐bonding) strengthens adsorption, viscoelasticity, and bioactivity	SBP‐NH_2_ 1 wt%; TA mass ratio 1:0.2 or 1:0.5; CA 2.5 wt% in oil; MCT oil 75 vol%; optimal = SBP‐NH_2_/TA = 1:0.5 + CA	Contact angle ↑: SBP‐NH_2_ 81.05° → 98.25°, SBP‐NH_2_/TA 88.35° → 91.15° (with CA); interfacial tension ↓: SBP‐NH_2_ 8.23 → 6.85 mN/m (with CA); 1:0.5 NPs show strongest adsorption; CA induces covalent linkage → thicker interfacial layer	Ultra‐Turrax 12,000 rpm, 2 min at 25°C; oil *φ* = 0.75; 3D printing nozzle 1.2 mm, extrusion 1 mm^3^/s; cuboid prints (20 × 20 × 10 mm)	Strong shear thinning; viscosity ↑ with TA and CA; *G*ʹ >> *G*ʺ over 0.1–10 Hz; *G*ʹ highest for 1:0.5 + CA; time sweep: *G*ʹ increases continuously 0–12 h	Droplet size: 18.3–42.2 µm (control) → smaller with CA; 1:0.5 + CA shows smallest and most stable sizes; stable 90 days at 25°C with negligible coalescence; centrifugation: no oil and no water release (1:0.5 + CA)	3D‐printed cuboids with smooth surfaces; 1:0.5 + CA shows highest fidelity and structural integrity; compact longitudinal sections	Highest gel strength 122.32 g·mm, hardness 128.04 g, adhesiveness −175.58 g/s; strong antioxidant effect (lipid hydroperoxide ↓ 44.21 → 10.08 mmol/kg); hexanal formation strongly reduced; strong antibacterial zones vs. *E. coli* and *S. aureus*	SBP‐NH_2_ alone unstable; 1:0.2 NPs weak gels; CA‐free samples show larger droplets and weaker networks; slight surface roughness remains in prints	H. Chen, Yu, et al. 2023
HIPE–GE1.5–Cit^3−^ (gelatin HIPE)	Hofmeister Na_3_Cit enhances gelatin chain bundling and viscoelasticity	Oil 75%; aqueous 25%; GE 1.5 wt%; Na_3_Cit 20 wt% (glycerol/water 1:1); resveratrol 0.1%	Droplet size *D*_3,2_ 7–10 µm; stronger junction zones; compact continuous gel; increased adsorption and elasticity	Homogenization 15,000 rpm, 5 min; GE heated 60°C × 30 min; cooling 4°C × 1 h; salt soaking 3 h	Strong shear thinning; *G*ʹ > *G*ʺ; *K*′ 1781.7 Pa, *n*ʹ 0.029; *K*″ 68.2 Pa, *n*ʺ 0.212; recovery >70%; LVR widened	DSC: *T*_c_ −28.55°C, *T*_m_ −7.33°C; *T*_d_ 125.49°C, Δ*H* 274.2 J/g; compression limit 0.878 cm; resveratrol retention: >80% (8 h, 90°C), ∼73% (4 days UV); release: 12% → 25% → 65% (oral → gastric → intestinal)	3D‐printed nutraceutical HIPE gels; heat‐resistant functional carriers	Best shape fidelity; strongest elasticity and toughness; high thermal/UV protection; controlled intestinal release	GE >1.5% limits ion availability and reduces uniformity; requires salt‐soaking step	L. Zheng, Li, Wang, et al. 2025
Ugi‐OA/ASNs covalent‐bond HIPPE	Schiff‐base covalent recognition between aldehyde‐alginate and aminated silica NPs	Ugi‐OA 0.2 wt% (aq); ASNs 0.15–0.2 wt% (oil); oil fraction 75%; dodecane continuous phase	Strong Ugi‐OA–ASN covalent anchoring; interfacial film thickness 0.591 µm → 1.154 µm; QCM‐D Δ*f* −32.57 Hz, elution 9.12%; adsorbed mass 545.54 mg/cm^2^; film thickness 5.45 nm	High‐shear homogenization 10,000 rpm, 5 min; printing via Eazao Bio printer; nozzle 0.6 mm, pressure 9.85 × 10^3^ Pa, speed 5–10 mm/s	Strong shear thinning; viscosity ↑ 100× with ASNs; *G*ʹ >> *G*ʺ; *G*ʹ >100 Pa across 10–1000 rad/s; LVR at 1% strain; ASNs yield gel‐like elasticity	Stable up to 6 months (only slight stratification at 0.15% ASN); droplet size sharply decreases with ASN addition; robust bridged network; stable even with only 10% water phase	Complex structures: “A,” serotonin symbol, multilayer snowflake; high shape fidelity and self‐support	Thick, rigid interfacial film; high viscoelasticity at low NP loading; excellent extrusion continuity; covalent film prevents collapse	Excess ASNs → droplet aggregation, deformity, reduced flow; Ugi‐OA alone cannot form HIPPE	Z. Wang, Huang, et al. 2023
PMpDA and PMpTA–soybean oil W/O HIPE	Surface‐modified pea‐protein microgels (dynamic covalent pDA and Schiff‐base TA) tune wettability and interfacial elasticity	Pea protein microgels 1–4 wt%; modified PMpDA/PMpTA; soybean oil 80%; water 20%; optimal: PMpDA 2%, PMpTA 1%	PMpDA hydrophobicity; PMpTA forms covalent imine network; droplet size 40–90 µm → <30 µm after modification (Figure 3b); stronger interfacial elasticity; compact flocculated network	Pre‐emulsification 15,000 rpm, 2 min → homogenization 20,000 rpm, 1 min; printing nozzle 0.84 mm, speed 25 mm/s, 25°C	Strong shear thinning; *G*ʹ >> *G*ʺ; highest *G*ʹ for PMpDA‐2% and PMpTA 1% (∼10^3^–10^4^ Pa, Figure 4b); *τ*_0_ ↑; fast recovery ≈90% (3ITT)	Creaming index ↓ from ∼45% → <10% (after modification); stable morphology for 14 days; PMpTA gives smallest droplets and minimal oiling‐off	3D‐printed self‐supporting grids and cylinders; PMpDA → rigid structures; PMpTA → fine resolution	Improved wettability tuning; high structural fidelity; strong viscoelastic networks; excellent thixotropy	Too high PMpDA (>2%) → brittle gel; too high PMpTA (>1%) → reduced flowability; large oil fraction limits payload types	Shahbazi, Jäger, et al. 2024
PWP–Cur–PC/soybean oil O/W HIPPE	Whey protein nanoparticle–polyphenol (Cur + PC) complexes enhance wettability, adsorption, oxidative stability, and printability	PWP (100 mg/mL), Cur (EE 97.05%; LC 5.17 mg/g), PC 0.5–10 mg/mL; oil *φ* 0.80	Contact angle *θ* 52.1° → 65.3° (PC↑); surface tension ↓; interfacial tension drops rapidly in first 200 s; AP ↑; Γ ↑; droplet size 23.67 → 13.34 µm (PC↑); *ζ*‐potential −50 to −60 mV	Homogenization 12,000 rpm, 2 min; PWP prepared at 85°C × 20 min; printing: nozzle 1.0 mm, speed 30 mm/s, 15 layers	Shear‐thinning; viscosity ↑ with PC (highest at 10 mg/mL); strong *G*ʹ > *G*ʺ; LVR enlarges with PC; *τ*_0_ highest for PC‐rich PWP–Cur–PC; 3ITT recovery high but incomplete	Centrifugal water loss decreases from above 20% → below 10% (PC↑); printed droplet size slightly ↑ after 7 days at 4°C; improved stability vs. PWP and PWP–Cur	3D‐printed cubes and internal hollow lines; retained structure for 7 days at 4°C	Superior oxidative stability (POV: 55.47 → 36.91 mmol/kg); MDA lowest at PC 10 mg/mL; controlled Cur release; tight interfacial layer; smallest droplet size; best print fidelity	Small oil leakage in PWP and PWP–Cur (not PC‐rich); color shift (pinkness) with PC; recovery not 100% after shear; too much PC increases aggregation	Ji, Sun, et al. 2025
GA–sunflower oil O/W HIPE	Gum Arabic (amphiphilic aggregates) forms Pickering‐like interfacial films and GA–drop flocculated network	GA 2–8 wt%, optimal 6 wt%; oil fraction 75 vol%; water 25%; pH ≈ 7	GA aggregates adsorb at oil–water interface; droplet size ∼15–40 µm → ∼10–20 µm (↑GA); thickened interfacial layer; strong depletion‐driven droplet clustering; *ζ* ≈ −30 mV	Pre‐emulsion 10,000 rpm, 3 min; homogenization 18,000 rpm, 2 min; 3D printing: nozzle 0.84 mm, speed 25 mm/s, layer 0.6 mm, 25°C	Strong shear thinning; viscosity ↑ sharply with GA; at 6% GA: *G*ʹ > 10^3^ Pa, *G*ʹ >> *G*ʺ; *τ*_0_ highest at GA 6%; 3ITT recovery ∼85%–90%; LVR extends with GA	Stable >30 days (minimal creaming); GA 2% unstable; GA 6%–8% keeps droplet size constant during storage; no oiling‐off in GA ≥4%	3D‐printed grids, cylinders, solid blocks; GA 6% → highest shape fidelity and smoothest surfaces	Clean‐label emulsifier; small droplet size; excellent thixotropy; strong viscoelastic network; precise deposition and layer integrity	GA 8% excessively viscous → reduced flowability; GA 2% poor stability; slight deformation in overhang structures	Liang et al. 2024
SPI–TA soy oil O/W HIPE (*φ* = 80%)	Noncovalent SPI–tannic acid complexation (H‐bond + hydrophobic) for 3D printing	SPI 10 mg/mL; TA 0–100 µmol/g; soy oil 80%; F0–F100 samples	TA ↓ hydrophobicity (*H*_0_: 162 → 53); *θ*_ow 80°→70°; particle size 141–188 nm; *ζ* −22 → −25 mV; stronger interfacial packing with TA ≥50	High‐speed shear 10,000 rpm, 60 s; 25°C; O/W HIPE formation	Gel‐like: *G*ʹ > *G*ʺ'; LVR widens (4.50% → 11.31%); frequency sweep: *G*ʹ dominant; viscosity shear thinning (0.1–100 s^−1^); high *η* for H100	Stable 0–14 days for all; F0 partial separation at 14 days; TA ≥50 µmol/g fully stable 45 days; droplet size decreases with TA	3D‐printed cylinders (Ø 20 × 10 mm); good resolution for TA ≥30; best fidelity in H100	Strong elasticity; dense polygonal droplets; high print fidelity; no nozzle clogging; hardness ↑ (16 → 35 g); chewiness ↑ (0.23 → 1.07 mJ)	Low TA (<30 µmol/g) → phase separation by 45 days; SPI‐only (F0) collapses after printing	W. Hu, Chen, et al. 2023
OMS/CHC–EGCG O/W HIPPE (*φ* = 0.75)	Ternary OMS–CHC–EGCG complex; electrostatic/H‐bond/hydrophobic assembly	OMS:CHC:EGCG = 60:15:4 (15–1); particle size 277.6 ± 9.8 nm; PDI 0.439; *ζ* 13.9 ± 0.37 mV; *θ*_ow 88.17°; complex 0.5%–3.5% w/v	EGCG compacts complex structure; *θ* near 90° → strong O/W adsorption; droplet size *D*_4,3_ 44.9 → 12.3 µm, *D*_3,2_ 22.42 → 6.50 µm (↑conc)	Homogenization 12,000 rpm, 2 min, 25°C; 3D printing: 0.40 mm nozzle, 15 mm/s, 25°C	Shear‐thinning (0.1–100 s^−1^); *G*ʹ >> *G*ʺ (elastic); *G*ʹ and *G*ʺ increase with concentration; rapid recovery after 100 → 1 s^−1^ shear	Stable 28 days at 4°C; centrifugation stable ≥1%; 0.5% shows slight oiling	3D‐printed shapes: cuboid, maple leaf, Superman model	Small droplets; high elasticity; excellent antioxidant activity: DPPH 56.23%–89.65%, ABTS 70.76%–91.78%; high print fidelity ≥2.5%	0.5% unstable; ≤2% poor texture retention; requires ≥2.5% for best resolution	Ji, Gao, et al. 2025
SBP‐NH_2_/OTA NNs ± CA O/W HIPE (*φ* = 0.75)	Covalent SBP‐NH_2_–OTA nanonetworks + CA Schiff‐base bridging	SBP‐NH_2_ 1 wt%; OTA mass ratio 1:0.1–1:0.7; optimal 1:0.5; CA 2.5 wt% in MCT; MCT oil 75%	Nanonetwork morphology; *θ* = 86.83° (NNs) → 93.17° (NNs@CA); CA enhances hydrophobicity and interfacial bridging; *D*_4,3_ decreases with OTA up to 1:0.5; smaller droplets with CA	Homogenization 13,000 rpm, 2 min, 25°C; oil phase: 100% MCT or 97.5% MCT + 2.5% CA	Strong gel‐like behavior; *G*ʹ 84 Pa (SBP‐NH_2_) → >1800 Pa at 1:0.5; *G*ʹ > *G*ʺ; shear thinning (0.1–100 s^−1^); viscosity ↑ with OTA (↓ at 1:0.7); CA further ↑ *G*ʹ and *G*ʺ; time sweep ↑ moduli over 12 h	Stable 90 days at 25°C (no phase separation); centrifugation stable (10,000 × *g*, 10 min); SBP‐NH_2_ alone fails; CA improves WHC and anti‐deformation	3D‐printed cuboid (20 × 20 × 10 mm), uniform gaps; improved resolution with CA	Dense interfacial layer; strong viscoelasticity; high gel strength: 159.34 g·mm, hardness 138.34 g, adhesiveness −71.34 g/s; excellent oxidative protection: LH 17.24 → 11.41 mmol/kg (with CA); antibacterial zones 28.45 mm (*S. aureus*), 19.3 mm (*E. coli*)	Excess OTA (1:0.7) ↓ modulus and viscosity; CA‐free systems less stable; SBP‐NH_2_ alone cannot form HIPE	H. Chen, Yu, et al. 2025
CP–FO O/W HIPE (*φ* = 0.75)	Collagen peptide crosslinked (TGase) forming adsorption monolayer on oil droplets	Collagen peptide 1–6 wt%; optimal 5 wt%; fish oil 75%; TGase 30 µL; sonication 45°C × 30 min	Interfacial film formation; droplet size ↓ 56.96 ± 2.58 µm → 6.70 ± 0.67 µm (5%); uniform droplets; dense accumulation; bridging flocculation; W/O/W at 1% (unstable)	Homogenization 8000 rpm, 2 min; surimi ink prepared with 10%–30% HIPE; printing: nozzle 1.0 or 4.0 mm, speed 25 mm/s, 25°C	Strong shear thinning; *G*ʹ > *G*ʺ (1–10 Hz); modulus ↑ with CP (1%–6%); 5% widest LVR; viscosity decreases with shear; 3ITT recovery ≈96% at 5%	Centrifuge (12,000 × *g*): 1% shows oil + water separation; 2%–6% no oiling; water precipitation decreases from 2% → 5%; stable structure at CP ≥5%	Surimi gel inks (10%–30% HIPE); inverted pyramid + dinosaur models; best printability at 20% HIPE	Viscosity reduction → smooth extrusion; improved continuity; lowest porosity and smoothest edges at 20%; miscibility with surimi matrix; softer texture for elderly/children	Excess HIPE (30%) ↓ mechanical strength; CP 1% unstable; surimi hardness and chewiness ↓ with HIPE addition (e.g., hardness 2.57 → 1.83 N)	S. Lu et al. 2024
HSPI–CG O/W HIPPE (*φ* = 0.75)	HSPI–CG complexes via H‐bonding, electrostatic + hydrophobic forces enhance adsorption and viscoelasticity	HSPI 1 wt% (heated 95°C × 15 min); CG 0–0.8 wt%; control HSPI 1.6 wt%; oil (soybean) 75%	CG ↑ *ζ*‐potential: −49.23 → −61.63 mV (0.2% → 0.8%); *γ* decreases over 3 h; surface pressure *π* ↑ with CG; droplet size: HSPI1.6% 32.2 µm; HSPI1%–CG: 26.30 → 13.49 µm (0.2% → 0.8%)	Homogenization 10,000 rpm, 2 min, 25°C; printing: FOODBOT‐D2; nozzle 0.84 mm, speed 6 mm/s, layer height 0.6 mm, fill 90%	Strong gel‐like structure; *G*ʹ > *G*ʺ (0.1–10 Hz); *G*ʹ and *G*ʺ increase with CG; apparent viscosity increases with CG; shear thinning (0.1–10 s^−1^); high viscosity at CG ≥0.6%	Storage: stable 14 days, slight oiling at 90 days for HSPI1.6% and 1%–0.2%; thermal: stable after 100°C × 15 min; freeze–thaw: only CG ≥0.4% stable after 1 cycle; all fail after 2 cycles	3D‐printed cylinders; CG ≥0.4% → no collapse after 14 days; HSPI1.6% collapses	Small droplets; strong viscoelasticity; improved self‐supporting; enhanced freeze–thaw and thermal stability; CG enables HIPPE formation at lower HSPI content	CG ≥0.8% too viscous to handle; HSPI alone unstable; emulsion fails after two freeze–thaw cycles	R. Li, Guo, et al. 2025
Heat‐treated gelatin–soy oil HIPE (*φ* = 0.80)	Heat treatment modulates gelatin MW, *H*_0_, EAI/ESI, and gelling ability	Gelatin extracted at 50°C/70°C/90°C, 2–8 h; gelatin 80 mg/mL; oil 80%	Stronger networks at 50°C–70°C; larger voids at 90°C; EAI/ESI and *H*_0_ peak at 5 h	Homogenization 8000 rpm, 90 s; printing nozzle 1 mm, 1 mm/s	*G*ʹ > *G*ʺ; highest *G*ʹ E‐50‐2; lowest E‐90‐8; RR: 95.51%, 95.55%, and 98.33%; shear thinning	Best stability and printing: 70°C (2–8 h); 90°C samples collapse; gel strength 79.94 → 25.22 g, hardness 600.7 → 208.4 g	3D‐printed Chinese knot models	High RR (up to 98%); tunable texture; good fidelity at 70°C	90°C gelatin too weak; rough printing at 50°C; reduced strength with long heating	Y. Wang, Zhang, et al. 2024
SPI microgel–CS/soybean oil O/W HIPE	SPI–CS microgel structuring for adjustable printability	SPI microgel 3%; CS 0%–0.5%; oil 75%–80%; NaHCO_3_ 0%–1%	Particle size 530 → 270 nm; *θ* 129.5°→103.1°; droplet size 14.9 → 23.7 µm (*φ* = 75% → 80%)	Pre‐shear 12,000 rpm, 3 min + HPH 500 bar for three cycles	Shear‐thinning; *G*ʹ >> *G*ʺ; viscosity 1240 → 2010 Pa·s (*φ* = 75% → 80%); 3iTT recovery 67%–80%	TSI ↑ with oil (80% least stable); TSI ↓ with CS/particle ↑	3D‐printed dolphin; thermo‐colored maple leaf	Accuracy 97.9% (ΦP 3%); stability 99.2%; tunable texture (gel strength 12.6 → 40.6 g	High oil → rough surface, leakage; low particle ≤1% → collapse	Wu et al. 2025
Gliadin–CMC HIPPE	Protein–polysaccharide complex tuning wettability and elasticity	G:CMC = 1:0 → 1:2; particles 1.5%; oil 75.4 ± 0.4%	Size 513 nm → 2–3 µm; *ζ* +7.9 → −49.6 mV; droplet size 32.1 → 8.8 µm (best at 2:1)	10,000 rpm, 5 min	*G*ʹ > *G*ʺ; shear thinning; recovery: 50.6% → 93.23% (2:1) → 67.2%	β‐Carotene retention ↑ to 42.9% (2:1); stable gel at moderate CMC	3D printing (1 mm nozzle, 15 mm/s)	Best fidelity at 2:1; high elasticity; bioaccessibility ↑ 12.3% → 37.0%	1:0 collapse; 1:1–1:2 weakened network, deformation	D. Zhang, Yang, et al. 2024
SC/TA–fish oil O/W HIPE	SC–TA complexation improves interfacial film strength	SC:TA mass ratio 2:1; SC 10 g/L; oil 16 mL; *φ* ≈ 0.76; buffer pH 6	TA ↑ interfacial strength; ↑ film thickness; ↓ droplet rupture; uniform small droplets	Ultra‐Turrax 10 000 rpm, 3 min; stored 4°C	Highest viscosity and moduli among SC/TA ratios; *G*ʹ > *G*ʺ; improved creep resistance; viscosity comparable to blank surimi	Stable during mixing/heating; SC‐only HIPE partially collapses; SC/TA retains droplets and prevents coalescence	Surimi gel strengthening and 3D printing cylinders (30%–80% infill)	Hardness 2255 ± 164 N; cohesiveness 0.82; gel strength 243 ± 15 g·cm; WHC 70%	Yellowing; TA oxidation risk	Y. Wang, Yu, et al. 2025
EWMG–KGM/XG HIPE	Protein–polysaccharide complex strengthening	3% EWMGs; KGM/XG ratios: KGM, 3/1, 1/1, 1/3, XG; oil 75%	Droplet size 30.2 → 10.6 µm; *θ* 65.9° (1/1); *ζ* < −30 mV; strong adsorption layer	7000 rpm, 3 min; printing 1 mm nozzle, 1 mm/s	*G*ʹ > *G*ʺ; viscosity ↑ with KGM/XG; critical strain 1.79% → 9.77%; shear thinning	Stable 5 days at 4°C; best at 1/1; slight oiling in XG‐rich	3D‐printed pyramid, *π*	High fidelity; strong network; small droplets	XG‐rich → delayed extrusion, residue; some oil leakage	Yu, Han, Xu, et al. 2024
LF–fish oil HIPPE	LF particle stabilization to improve surimi gel and printability	LF 1.5%; oil 80%; droplet size 4.41 → 9.44 µm; *ζ* −29.8 → −12.3 mV (pH 3–9)	Strong LF adsorption; hydrophobic + electrostatic; robust interfacial film	10,000 rpm, 3 min	*G*ʹ > *G*ʺ; viscosity ↑; strong shear thinning; best 3ITT recovery	Stable across pH 3–9, NaCl 0–200 mM, heating, freeze–thaw; 30 days storage	Surimi gel and 3D printing	Gel strength 717.4 vs. 606.3 g·cm; WHC ↑; CL ↓; smooth prints; L* 85.29	Droplet growth at high pH/ionic strength	Sun et al. 2025
β‐CD/CS HIPE (O/W, *φ* = 0.75)	β‐CD–CS electrostatic + H‐bond co‐assembly for stable HIPE and printing	β‐CD 1.2 wt% + CS 0.08–0.40 wt%; optimal: 0.32 wt% CS @ pH 5; oil 75%	Interfacial membrane formation; contact angle ∼90° at pH 5; *ζ*‐potential 35 → 85 mV (↑ with CS)	12,000 rpm, 3 min	Strong shear thinning; *G*ʹ > *G*ʺ; *τ*_0_ ↑ with CS; highest at 0.32% CS; excellent 3ITT recovery (≈ full recovery)	Centrifugally stable; 4‐month storage stable (CS ≥0.16%); instability at pH 7	3D printing grids + cuboids + complex models	Smooth extrusion; high fidelity; uniform filaments; best performance at pH 5 + 0.32% CS	pH 7 → poor *G*ʹ, poor recovery, droplet coalescence	X. Li et al. 2023a
FP–AG HIPE	FP–AG electrostatic complexing	FP–AG 1.5%; oil 74.4%; pH 4 (*θ* 87.9°)	Strong binding (*K* = 6.87 × 10^6^ M^−1^); thick interfacial layer	12,000 rpm, 10 min	*G*ʹ >> *G*ʺ; shear thinning; recovery 88.4%	Highly stable at pH 4	3D printing (grids, bear)	Smooth extrusion; strong structure; Cur retention 32.7% UV, 59.6% at 80°C	Weak/collapsed at pH 8	Y. Chen, Wang, et al. 2023
PeaP–BCNF HIPE + SA/TGase double‐network gel	BCNF reinforces PeaP interface; SA–Ca^2^^+^ + TGase form dual network	PeaP 5%; BCNF 0%–0.16%; oil 75%; SA 0.5%; TGase 12.5 mg/mL	Interfacial tension 11.91 → 9.49 mN/m; droplet size 27 → 11.3 µm; thicker interfacial layer	13,000 rpm, 2 min; gelation via TGase + Ca^2^^+^	*G*ʹ and *G*ʺ ↑ ∼10× after dual‐network; viscosity max 208 Pa·s (0.16% BCNF); fracture stress 0.015 MPa	Heat‐stable (25°C–100°C); freeze–thaw syneresis 14.5% → 24.9% (BCNF ↑)	3D printing (octopus, turtle)	High strength; strong self‐support; sharp edges; rigid solid gel	High BCNF worsens freeze–thaw stability	Y. Wang, Liu, et al. 2023
Casein/pectin HIPE	Hybrid particle thickening and interfacial adsorption	Casein 3% + pectin 1%–5%; oil 75%	Hybrid aggregates (∼10 and ∼100 µm); interfacial coverage; hydrogen bonding	8000 rpm, 1 min	Viscosity 1316 → 4302 Pa·s (0% → 4% pectin); *G*ʹ > *G*ʺ; gel strength 10.37 → 21.19 g (1% → 5%)	Stable shape; viscosity ↑ with pectin	3D‐printed cylinders	Good fidelity; strong structure; hardness ↑	Casein‐only collapses; excessive pectin → uneven filaments	A.‐Q. Bi et al. 2022
PLLA/PCL–h‐SiO_2_ W/O HIPE	Hydrophobic silica for printable HIPE and hierarchical porosity	PLLA + PCL (4–6 wt%); h‐SiO_2_ 2.5–3.0 wt%; water 70%–75%; droplet 17.8–20.1 µm	h‐SiO_2_ adsorption; thin polymer films rupture → pore throats	Manual emulsification 30 min; print via 0.9 mm needle; dry 48 h	Shear‐thinning; *G*ʹ > *G*ʺ <80% strain; yield‐like flow; instant structure recovery	Stable ≥1 month; open‐cell only at *φ*_w_ ≥75%; closed‐cell at *φ*_w_ ≤70%	3D‐printed PLLA/PCL scaffolds	Porosity 98.0%–98.3%; hierarchical pores (macro/meso/micro); rapid drug release ∼80% at 2.5 h, >98% at 10 h; supports mBMSC adhesion/proliferation	High polymer viscosity (>92 mPa·s) prevents HIPE formation; unstable at low SiO_2_ or very high polymer	T. Yang et al. 2017
W/O polyHIPE for carbon lattices	Emulsion‐templated photopolymer for porous carbon	EHA/IBOA/TMPTA (39.7%/39.7%/15.9%); surfactant 4.7%; PI 5%; water 80%–87.5%	Pore (polyHIPE) 21 → 34 µm; carboHIPE 15 → 12 µm after pyrolysis	DLP printing (60 µm layers); carbonization 500°C–800°C	Porous network; density 0.14–0.48 g/cm^3^; modulus 1.28 MPa; D/G 0.6 → 0.8	Macro‐shape retained; ∼97% shrinkage uniform; 80% prints best	3D‐printed carboHIPE lattices	Tunable porosity; lightweight; hierarchical pores	85%–87.5%: curing defects; low char yield 3%–6%	Sengokmen‐Ozsoz et al. 2025
Acrylate polyMIPE for SLA	Tartrazine tunes light absorption and pore size	EHA/IBOA/TMPTA (39.7%/39.7%/15.9%); surfactant 4.7%; PI 5%; water 80%; tartrazine 0%–0.08%	Surface pores 27.18 → 34.02 µm; internal 19.7–25.2 µm; interconnects 1.99–3.33 µm	SLA; 30 µm layers; 8–10 s exposure	Stiffness 0.74–1.10 MPa	Porosity 63.9% → ∼56% (↓ with tartrazine)	3D‐printed trabecular scaffolds	Best cell growth at 0.08%; high fidelity porosity	High tartrazine ↓ HIPE stability; thin struts merge	Sengokmen‐Ozsoz et al. 2024
Write more briefly	Optimized HA:TA ratio (7:1) enhances interfacial activity and network formation	Pea protein isolate + hyaluronic acid + tannic acid (P‐H_7_‐T_1_ most effective)	Hydrogen bonding + hydrophobic + electrostatic interactions; lowest *γ* achieved at HA:TA = 7:1; rapid adsorption to interface	Noncovalent assembly → small droplets → densely packed HIPEs; *φ* = 75% phase, homogenized at 12,000 rpm	Strong shear thinning; *G*ʹ >> *G*ʺ; rapid thixotropic recovery; highest moduli for P‐H_7_‐T_1_	Excellent storage (28 days) and thermal (90°C) stability; minimal coalescence	Edible 3D printing inks; curcumin delivery with high digestibility and bioaccessibility	Smallest droplets; strongest network; best print fidelity; improved lipid digestion and curcumin bioaccessibility (∼79%)	Requires precise HA:TA ratio; high TA reduces stability and printability	Z. Li et al. 2024
PPI–PC HIPE	Heat–pH‐modified PPI + PC for stronger networks	PPI 2% (pH 10, 100°C); PC 1%; oil 80%; pH 7	Smaller droplets; thicker interfacial film; steric–electrostatic stabilization	12,000 rpm, 2 min	*G*ʹ >> *G*ʺ; high viscosity; 3ITT recovery 73%–94%	Stable 7 days at 4°C; heat‐stable 50°C–90°C	3D printing cubes and turtle shapes	Highest modulus; best fidelity; no collapse	PPI alone collapses; optimal only at PC = 1%, pH 7	R. Zhao et al. 2025
m‐HAp/m‐SiO_2_–PCL HIPE	Dual‐particle stabilization for hierarchical scaffolds	PCL 6%–12%; m‐HAp 7.5%–15%; m‐SiO_2_ 1.5%; water 75%	Rod HAp + SiO_2_ adsorption; droplets 10–50 µm	3500 rpm; print 30 mm/s; 0.34–0.51 mm nozzle	Shear‐thinning; *G*ʹ > *G*ʺ; viscosity ∼4746 Pa·s (0.01 s^−1^)	Porosity 94.6%–96.7%; stable >1 month	Bone scaffolds, drug release	Hierarchical pores; modulus 42–516 kPa; ibuprofen release 75%–96%; good cytocompatibility	Uses CH_2_Cl_2_; high HAp weakens scaffold	Y. Hu et al. 2019
PLA‐GMA/PEGDA HIPE	Photocurable HIPE for DLP	PLA‐GMA (Mn ∼5400), PEGDA, L‐121, toluene; water 85%	Lower *γ* with toluene; pores 1–10 µm	DLP (405 nm); layer ∼90 µm	Viscosity tunable by diluent; fast curing	Shrinkage 8%–10%; pores retained	Porous biodegradable scaffolds	High porosity; fast UV curing; DLP‐printable	Sensitive to viscosity; shrinkage; diluent‐dependent structure	Choi et al. 2022
PPI–HMP–EGCG PE (*φ* = 0.52) and HIPPE (*φ* = 0.83)	Protein–pectin–polyphenol complexes strengthen interface	1% PPI–HMP–EGCG; cinnamaldehyde 0%–100%; tea oil carrier	Schiff‐base crosslinking; elastic interfacial film (*G*ʹ > *G*ʺ)	12,000 rpm, 2 min + 10,000 × *g*, 5 min	Shear‐thinning; *G*ʹ↓ as cinnamaldehyde ↑	Stable ≤50% cinnamaldehyde (30 days at 4–25°C); unstable ≥75%	3D printing (lines, cuboids, turtles)	Smooth extrusion; low volatile loss (4%–11%)	Poor printing at ≥50% (PE) and 100% (HIPPE)	Feng et al. 2022
Cod protein HIPE	Protein adsorption–gel network	Cod protein 10–50 mg/mL; oil 85%	AP 70%–96%; *θ* ≈ 77°; *γ* ↓; dense interfacial film	8000 rpm, 100 s; nozzle 1 mm	*G*ʹ > *G*ʺ; strong shear thinning; viscosity ↑ with protein	Stable 7 days at 4°C; best at 40–50 mg/mL	3D food printing	High fidelity; strong gels; good extrusion	Low protein weak; *φ* ≥ 92% fails	X. Li et al. 2020
WPI–GA‐C HIPE	Maillard conjugate strengthens interface	WPI–GA‐C 1%–4%; oil 74%–84%; optimal: 3% + 80%	Thick elastic film; irreversible adsorption; *D*_4,3_ 5.94 µm, PDI 0.26	14,000 rpm, 5 min	Shear‐thinning; *G*ʹ >> *G*ʺ; *η* (0.01 s^−1^) 93 → 370 Pa·s	Stable 30 days; centrifugation stable; 0.5%/84% fail	3D food printing, dysphagia foods	High fidelity; strong self‐support; minimal droplet growth	Collapse at low WPI–GA‐C or *φ* = 84%	Kan et al. 2023
LM–HIPEG	Carbopol–Ga^3^^+^ coordination stabilizes high‐*φ* LM	EGaIn 82.5%; Carbopol gel 17.5%	Ga_2_O_3_ shell (∼5 nm) + Carbopol (∼10 nm); strong repulsion	25,000 rpm, 5 min; DIW 370–430 kPa	*G*ʹ 1.1 × 10^4^ → 4.1 × 10^4^ Pa; shear thinning	Stable 24 h at 100°C; −30°C load ≥50 g	LM DIW printing; circuits	Resolution 210 µm; *φ* high; conductive (5 × 10^5^ S/m)	*φ* > 90% unstable; *φ* < 74% weak	Lin et al. 2024
FSG–fish oil HIPE	MTGase‐crosslinked FSG particles stabilize ω‐3 HIPE	FSG 10–60 mg/mL; oil 80%; *θ* 67.2°; *ζ* −13.1 → −20.4 mV	*γ* 22.17 → ↓; *π*_3600_ 11.01 → 14.67 mN/m; AP 75.8% → 94.2%; droplets 53.98 → 8.21 µm	15,000 rpm, 60 s; MTGase 3 U/g	*G*ʹ >> *G*ʺ; *n* 0.44 → 0.02; *K* 13.4 → 323.6 Pa·s*^n^*; *τ*_f_ 31 → 273 Pa	Centrifuge‐stable ≥40 mg/mL; thermal 121°C; freeze–thaw stable (30–60 mg/mL); oxidation ↓ (MDA 41.25 → 14.94 µmol/kg)	Surimi 3D printing; ω‐3 enriched gels	High fidelity; oxidation protection; finer nozzle printing	FSG ≥50 mg/mL ↓ surimi gel strength	Y. Wang, Hu, et al. 2024
CN‐HAMA HIPE droplets	H_2_O_2_‐triggered shell disruption → shear‐activated release	Core: sunflower oil + Nile Red; Shell: CN 0.4%–1%, HAMA 0.5%–2%; APS/TEMED crosslinking	Dual‐network shell; oxidant cleavage weakens crosslinks; size ∼40–80 µm	Water‐in‐oil‐in‐water microfluidics; UV + redox crosslinking	Shear‐dependent rupture; stiffer shells at ↑HAMA	Stable at RT; rapid rupture under H_2_O_2_ (0.5%–1%) + shear	Shear‐activated drug/bioactive release	Tunable release (oxidant + shear); robust shell pre‐activation	Requires oxidant + shear; high HAMA slows release	X. Huang et al. 2023
LMPC/CPC HIPE	Insect proteins stabilize 80% O/W HIPE	Protein 1%–3% (LMPC/CPC); oil 80%	*ζ* −28.5 → −42.7 mV; droplets 15–58 µm; heat → larger aggregates	15,000 rpm, 2 min	*η* (0.1 s^−1^) 779–2300 Pa·s; *G*ʹ 450–1670 Pa; *τ*_y_ 139–399 Pa; recovery 63%–99%	Stable 14 days at 25°C; oil loss <8%	3D‐printed fat analogues	Strong structure; good fidelity; sustainable proteins	LMPC‐ThT <2% → W/O; CPC lower recovery	Ballon et al. 2025
PPI–inulin HIPE	Maillard conjugate strengthens interface	2% PPI–inulin; oil 80%	Droplets 17–44 µm; *ζ* −30 → −40 mV	12,000 rpm, 2 min	*G*ʹ >> *G*ʺ; *G*ʹ 1100–1300 Pa; recovery 80%–88%	Stable *φ* = 75%–82.5%; 85% coalesces	3D food inks	High fidelity; strong elasticity	PPI alone collapses	W. Jiang et al. 2023
SPI–CMC HIPE	SPI/CMC electrostatic complexes strengthen interface and structure	Oil 80%; β‐carotene 0.1%; SPI/CMC ratios 20:1 → 1:1	Droplets 65.15 → 22.06 µm (optimum 2:1); dense film ↓ coalescence	12,000 rpm, 2 min	Shear thinning; recovery 89.43% (2:1); *G*ʹ > *G*ʺ; strongest viscoelasticity at 2:1	β‐Carotene EE 73.17% → 94.52%; bioaccessibility 16.55% → 31.32%; thermal and storage stability highest at 2:1	3D food printing (cross + cube shapes)	High fidelity at 2:1; smooth filaments; strong self‐support	1:1 → collapse, larger droplets, low *G*ʹ; excess CMC reduces stability	Shen et al. 2025
WPI/WPIA HIPE	Protein fibrillation ↑ stability and printability	WPI/WPIA 0.5%–2%; corn oil 80%; pH 3 or 7	WPIA *θ* ∼92°; WPI *θ* ∼68°; IFT 12.8 vs. 12.0 mN/m; denser shell	Acid–heat (24 h, pH 2, 60°C) for WPIA; 10,000 rpm, 30 s	*G*ʹ > *G*ʺ; at 2%: WPI *G*ʹ 1302 Pa, WPIA 1154 Pa; τy WPI 35.7 Pa, WPIA 44.5 Pa	WPIA stable to heat (90°C), salt ≤300 mM, 28 days; WPI unstable pH 7	3D printing (cubes, letters, lotus)	WPIA: better fidelity, storage stability, and recovery (>100%)	WPI collapses; WPIA pH 7 weak, oil leakage	C. He, Xu, et al. 2024
Zein–SPI composite HIPPE	Ethanol‐induced zein/SPI co‐aggregation → colloidal stabilizer	Zein/SPI (0%–100% SPI); total particles 1%–3% w/v; oil 80%	Droplet size >80 → 20–30 µm with SPI↑; tighter packing; strong steric layer	Stirring + homogenization (12,000 rpm, 2 min)	*G*ʹ >> *G*ʺ; strongest at 50%–75% SPI; *τ*_y_ increases with SPI; shear thinning	Stable 30 days; lower coalescence; oil leakage minimal at 50%–75% SPI	3D printing (fat‐mimetic structures)	High fidelity at 50%–75% SPI; robust network; small droplets	Pure zein unstable; ≥90% SPI reduces elasticity	L. Zhang et al. 2022
ZTS O/W HIPE	Zein–TA–SA ternary complexes stabilize interface	1.8% ZTS; oil 75%; ZT‐0.4 + SA (1:1–5:1)	Droplets 33.47 → 19.18 µm; *ζ* −60 mV; dense shell	15,000 rpm, 6 min	*G*ʹ > *G*ʺ; viscosity ↑ with SA	Stable pH 2–11; NaCl 0–1000 mM; β‐CE retention 53.6% (28 days)	3D printing; β‐carotene delivery	Small droplets; strong stability; good print fidelity	Unstable at pH 1; needs ≥1.2%–1.5% ZTS	X. Liu, Xie, et al. 2023
WPI–SA HIPE	WPI–SA complexes strengthen interface	WPI 2%–6%; SA 0.05%–0.20%; oil 75%–85%	Droplets 35 → 12 µm; *ζ* −22 → −38 mV	12,000 rpm, 2 min	*G*ʹ 350 → 1800 Pa; *τ*_y_ 45 → 128 Pa	Stable 30 days; SA ≥0.1% prevents creaming	3D printing	High fidelity; firmer structure	Needs SA; >0.2% too rigid	J. Zheng, Xiong, et al. 2024
CNC–HIPPE	CNC rod particles stabilize interface	CNC 0.5%; oil ∼80%; 50 mM NaCl	Dense CNC shell; irreversible adsorption	High‐pressure homogenization (50 MPa) + centrifugation	*G*ʹ > *G*ʺ; strong shear thinning; high τy	Stable 22 days; no oiling‐off	3D printing	High fidelity; low CNC use; good elasticity	>0.75% CNC → poor printing; high salt → blockage	Ma et al. 2022
RP–CMC (RC) HIPPE	Protein–polysaccharide complexes (pH cycle) tune wettability via DS	RC 0.7/0.9/1.2; 2.5% RC; oil 80%–90%	*γ* ↓: 18.63 → 12.49 mN/m; *θ* 163° → 96° (ideal ∼90°); Dh 634 → 132 nm	13,000 rpm, 60 s	*G*ʹ > *G*ʺ; *G*ʹ ↑ with concentration; optimal at 2.5% RC; strong shear thinning	Stable 10 days at 4°C; heat‐stable 90°C/30 min; best stability at DS 1.2	3D printing (cylinders, complex shapes)	Best fidelity at DS 1.2; strong gel network; tunable viscosity	Unstable at pH 3; low RC (≤1%) → droplet deformation; Φ >85% → coalescence	Wan et al. 2021
ODSA–CD‐MOF HIPE	MOF–Pickering stabilization	5 wt% OCM; oil *φ* = 0.8; pH 10–12	Contact angle ∼92°; dense MOF shell; 3D network	10,000 rpm, 0.2–2 min	*G*ʹ >> *G*ʺ; strong shear thinning	Stable 120 days; droplet 7–9 µm; freeze–thaw stable	3D printing	High fidelity; WHC ∼98%; long‐term stability	Needs alkaline pH; >5% OCM reduces stability	Y. Zhang and Yu 2023
ε‐PL‐TOCN/camellia oil O/W HIPPE (82%)	ε‐PL grafting tunes CNF wettability for HIPPE formation + 3D printability	0.8 wt% ε‐PL‐TOCNs (pH 3); camellia oil 82% *φ*	Higher contact angle (79° → 68°); increased interfacial tension; CNF network around droplets; *ζ* = +51 mV	Pre‐emulsion (18,000 rpm, 1 min) → oil feeding at 9000 rpm; DIW printing (15–20 kPa, 10 mm/s)	Shear‐thinning; *G*ʹ > *G*ʺ; higher *φ* → higher *G*ʹ	Stable ≥30 days; droplet size 41.8 ± 7.1 µm (82% *φ*)	3D‐printed edible shapes; porous foams (with *n*‐hexane)	Food‐grade; strong stability; good viscoelasticity and print fidelity	Needs pH 3; unstable <0.8% stabilizer	S. Zhang, Chen, et al. 2023
HA–MCC/soybean oil O/W HIPE‐gel	HA–MCC complexation stiffens network; HA Mw + conc. tune printability	MCC 5%; HA 0.1%–1% (L‐HA 80–200 kDa; H‐HA 200–600 kDa); soybean oil 75%	HA–MCC hydrogen bonding; HA chain entanglement; reduced droplet size; stronger steric stabilization	High‐shear mixing (10,000 rpm); oil‐fed pump (60 drops/min); 3D printing (0.8 mm nozzle, 28 mm/s)	Strong shear thinning; *G*ʹ > *G*ʺ; *K* ↑ sharply with HA; *n* ≈ 0.11–0.21	H‐HA1.0: very low instability; WHC >90%; printing stability ∼98%	3D‐printed food designs (flowers, bows, cubes)	High shape fidelity; elastic gel texture; clean‐label	Requires ≥1% HA; MCC alone collapses	Z. Chen, Sun, et al. 2025
PPI–soybean oil HIPE	PPI level controls network and printability	PPI 4%–12%; oil:water 3:1	Higher PPI → smaller droplets, stronger network	14,000 rpm, 2 min	Shear‐thinning; *G*ʹ > *G*ʺ; PPI↑ → viscosity↑	Stable 30 days; best at 12%; poor freeze–thaw	Extrusion printing	High precision, strong gels	Low PPI collapses; not freeze–thaw stable	C. Bi et al. 2025
ATMs (alginate–coumarin)/O/W PE	UV‐dimerization strengthens particle–particle network	ATMs 0.1 wt%; oil *φ* = 0.5–0.8 (best at 0.8)	Post‐UV: smaller droplets (∼16.8 µm), thicker interfacial film, higher adsorption; IFT ↓ to 9.31 mN/m	Homogenization 10,000 rpm, 3 min	Strong shear thinning; *G*ʹ > *G*ʺ; post‐UV viscosity ∼2×; higher yield stress	Best stability at *φ* = 0.8; stable shapes upon inversion	Direct 3D printing in air/oil (0.6‐mm nozzle; 15 kPa; 8 mm/s)	Excellent shape retention; high mechanical strength; no additives needed	Requires UV step; pre‐UV weaker interface	X. Zhao, Yang, et al. 2023
GMP–soybean oil PE	GMP self‐gels to stabilize droplets	GMP 1.5%–3%; K^+^; oil 75%	GMP network traps droplets	Heat–cool gelation; emulsify	*G*ʹ > *G*ʺ; strength ↑ with GMP	Stable >7 days	3D‐printed degradable scaffold	Degradable; tunable; good fidelity	Weak at low GMP; K^+^‐dependent	Q. Zhao, Fan, et al. 2023
MP–CS O/W HIPE	CS modulates MP adsorption + network density	MP 1.5 wt%; CS 0–1.5 wt%; soybean oil *φ* = 0.75	CS ≤0.9% ↓IFT, strengthens interfacial film; CS >0.9% hinders MP adsorption	Homogenization 12,000 rpm, 120 s	Shear‐thinning; *G*ʹ↑ at 0.6% CS (∼1034 Pa); fast 3ITT recovery	Best stability at 0.6%–0.9% CS; excessive CS → large droplets	3D‐printed food structures	Improved print fidelity, elasticity, shape retention	Poor printability at CS >0.9%; freeze–thaw sensitive	F. Zhang, Wang, et al. 2024
hWPI–oleogel O/W HIPE	Oil gelation + hWPI aggregates for strong HIPE network	hWPI 5%; soybean oil 80%; GM 0%–6%; fucoxanthin	GM crystallizes at interface; smaller droplets (17 → 5 µm); tighter packing	Homogenization 7000 rpm, 1.5 min	Strong shear thinning; *G*ʹ > *G*ʺ; GM↑ → higher moduli; >90% recovery at ≥4% GM	Stable 14 days; salt‐tolerant; freeze–thaw stable at ≥5% GM	Extrusion 3D printing (1 mm nozzle)	Excellent printability at 4%–6% GM; high encapsulation (≈90%); fucoxanthin half‐life ↑2.5×	No printability without GM; oil leakage at ≤2% GM	Shang et al. 2023
CNC/sunflower oil HIPE	CNC network + high *φ* forms printable gel	CNC 2–6 wt%; oil *φ* = 0.65–0.80	CNCs form rigid interfacial layer + percolated network; droplets tightly packed	High‐shear mixing 10,000 rpm, 3 min	Strong shear thinning; *G*ʹ > *G*ʺ; moduli ↑ with CNC	Stable at *φ* ≥0.75; small droplets; minimal coalescence	Direct ink writing (0.41‐mm nozzle; 15 mm/s)	Good shape fidelity; tunable stiffness; clean‐label	Low *φ* (<0.70) not printable; brittle structures at high CNC	C. Li, Zhu, et al. 2025
pH‐PP/CMC/sunflower oil O/W HIPE	pH‐cycle unfolds PP + CMC thickening for stronger HIPE	PP 4%; CMC 0%–0.3%; oil *φ* = 0.75	pH‐cycle ↑ hydrophobicity; CMC ↑ interfacial film thickness; droplets ↓ to 12 µm	Shear 13,000 rpm, 3 min	Shear‐thinning; *G*ʹ > *G*ʺ; CMC↑ → moduli↑; high recovery after 50% strain	Stable 28 days; good heat stability; smaller droplet growth with ≥0.2% CMC	Extrusion 3D printing (0.4‐mm nozzle)	Best fidelity at 0.3% CMC; strong gels; fine droplet size; reduced IFT	Poor freeze–thaw stability; PP alone not printable	L. Xu et al. 2024
CNF/NCh–oil HIPPE	Oppositely charged CNF/NCh strengthen interface + network	0.5% CNF/NCh; oil 75%–88% (cyclohexane 89%)	Interfacial adsorption + fibril network	Pre‐emulsion 22,000 rpm → oil feeding 6500 rpm	Shear‐thinning; *G*ʹ > *G*ʺ; *τ*_0_ ∼30 Pa	Stable ≥60 days	DIW 3D printing; molded shapes; aerogels	Super‐high *φ*; low stabilizer; excellent printability	Loses stability at 89% (sunflower oil)	Cheng et al. 2025
Egg‐white microgel HIPE (*φ* = 75%)	Heat‐induced microgels stabilize O/W HIPE	EWMG (70°C–90°C; 5%–10% EW); 75% soybean oil	Smaller/softer microgels (90°C, 5%) adsorb tightly; ↑ hydrophobicity	Homogenization 7000 rpm, 2 min	Shear‐thinning; *G*ʹ > *G*ʺ; top recovery at 90°C–5%	Good heat + centrifuge stability; smallest droplets at 90°C–5%	3D printing (1.2‐mm nozzle)	Best fidelity, no collapse at 90°C–5%	High EW → large droplets + oil leakage	Yu, Han, Liu, et al. 2024
CO–XG/soybean oil PEG (O/W)	CO–XG complexing strengthens particle adsorption + gel network	CO 4%; XG 0.5%–3%; oil–water 3:7 → 7:3	Higher XG → near‐90° contact angle, stronger hydrogen bonding, denser droplet packing	Homogenization 20,000 rpm, 3 min	Strong shear thinning; *G*ʹ > *G*ʺ; XG↑ → viscosity↑, recovery↑	Stable 60 days; WHC/OHC↑; smaller droplets at high XG	Extrusion 3D printing (0.25‐mm nozzle)	Excellent fidelity at ≥2.5% XG; stable, self‐supporting inks	Low XG (<1%) collapses; high viscosity reduces flowability	Z. Zheng, Ma, et al. 2025
G/SNP–corn oil HIPE	Centrifugation + NaCl tuning	G/SNPs (1:3); corn oil; NaCl 0–200 mM	Core–shell G/SNPs adsorb strongly; NaCl ↓ repulsion → smaller droplets	12,000 rpm pre‐emulsion → 10,000–15,000 × *g* centrifuge	Shear‐thinning; *G*ʹ >> *G*ʺ; max at 100 mM NaCl	*φ* up to 81.8%; excellent heat/light stability; high astaxanthin retention	3D printing; best fidelity at 100–150 mM NaCl	Simple; strong gels; high bioaccessibility (∼42%)	200 mM NaCl causes droplet fusion	Song et al. 2023
ZGNP–soybean oil HIPPE (75%)	GA modifies zein → better wettability + Mg^2^^+^ crosslinking	ZGNPs 0.5%–2.5%; oil 75%; MgCl_2_ 0–90 mM	GA coats ZNPs + migrates to interface; Mg^2^^+^ reduces repulsion + forms salt bridges	12,000 rpm, 2 min	Shear‐thinning; *G*ʹ >> *G*ʺ; Mg^2^^+^ ↑ moduli (∼5×)	No creaming 20 days; droplet size stable at ≥1.5% ZGNPs	3D printing (0.40 mm nozzle, 15 mm/s)	High fidelity at 30–90 mM Mg^2^^+^; strong recovery	No Mg^2^^+^ → collapse; high Mg^2^^+^ → larger droplets	Qiu et al. 2023
QP–CA/SA/PC HIPE (vegan)	Soluble QP–polysaccharide complexes at pH 6 ↑ adsorption + repulsion	1% QP; 1% polysaccharide (CA/SA/PC); corn oil *φ* = 75%–84%	CA > SA ≈ PC: stronger charge, smaller droplets, thicker interfacial layer	15,000 rpm, 2 min	Shear‐thinning; *G*ʹ > *G*ʺ; sharp modulus↑ at pH 6; CA highest	Excellent heat, freeze–thaw, 7‐day storage stability; CA best	Extrusion 3D printing (1‐mm nozzle): square and butterfly shapes	High *φ* (84%) with only 1% complex; best fidelity for QP/CA	QP alone weak; fails at *φ* ≥87%; pH‐sensitive	Zhong et al. 2024
Meso‐zein HIPE → spray‐dried powder	UHPH + meso‐zein form dense, elastic interfacial films	Olive oil + β‐carotene; meso‐zein 10–30 mg/mL	Droplets ↓ to ∼1.5 µm; thick meso‐zein layer	UHPH (100–150 MPa) → spray drying	Rehydrated gel‐like HIPE; strong viscoelasticity	High βC protection; good storage	Powdered delivery system	Solvent‐free; stable; rehydratable	Not 3D printable; requires UHPH	C. Wang, Jin, et al. 2023
LF–EGCG–BP ternary covalent HIPE	BP strengthens LF–EGCG interfacial film	LF–EGCG + beet pectin; fish oil 80%	Droplets ↓; *ζ*‐potential ↑; stronger elastic films	Ultra‐Turrax 10,000 rpm; Maillard‐type conjugation	Higher *G*ʹ, viscosity, recovery (up to 94.7%)	Lower LPO/MDA; lower TSI; smaller droplets	Stable 3D‐printed constructs	Strong gel network; best at BP 20:1	High BP causes viscosity, reduced adsorption	Sun et al. 2024
GLT–CNC–TA HIPE	TA reinforces GLT/CNC network	GLT 1%; CNC 0.6%; TA 0%–1.2%; soybean oil	TA (0.6%) ↑ wettability and layer thickness	15,000 rpm; heat 90°C; centrifuge → HIPE gel	*G*ʹ >> *G*ʺ; max strength at 0.6% TA	High OHC; good oxidation resistance	3D printing (1.2 mm)	Strong gel; converts low‐oil → HIPE; best at 0.6% TA	Too much TA → aggregation, leakage	H. Zhang et al. 2025
JP/OP–sunflower oil HIPPE	JP/OP synergy strengthens adsorption + network	JP:OP = 3:1–1:3; total 3%; oil *φ* = 70%–85%	Optimal ratio (1:1) → dense layer, small droplets	Homogenization 10,000 rpm, 2 min	Strong shear thinning; *G*ʹ >> *G*ʺ; highest at 1:1	Stable 30 days; minimal coalescence; good oxidative stability	3D printing (1 mm nozzle, 20 mm/s)	Excellent fidelity at 1:1; super‐stable at *φ* = 85%	Extreme ratios → large droplets + weak gels	Wu et al. 2022
PeaP–HPS HIPE gel	pH‐modulated PeaP + HPS network strengthening	PeaP 5%–10%; sunflower oil *φ* = 75%; HPS 2%–4%; pH 3–11	pH alters solubility, adsorption; pH 11 → lowest IFT; pH 3 → hydrophobic forces	Homogenization 13,000 rpm; HPS gelatinized at 85°C	*G*ʹ >> *G*ʺ; stronger at pH 3 and 11; ↑HPS → ↑moduli	Best holding at pH 3 and 11; 10% PeaP + 4% HPS → no leakage	3D printing; best fidelity at pH 11 (10% PeaP + 4% HPS)	Tunable by pH; strong gel at high PeaP/HPS	Weak printability at low PeaP/HPS; brittle at pH 5	Y. Wang, Yang, et al. 2024
SBP–EGCG–algal oil HIPPE (75%–88%)	EGCG–protein covalent complex gives dense shells + antioxidant network	SBP–EGCG (0.5%–2%); algal oil 75%–88%; astaxanthin	Smaller particles, *θ* ≈ 88°; dense shell + continuous network; lowest IFT	10,000 rpm, 2 min	*G*ʹ >> *G*ʺ; shear thinning; ≥1.5%–2% → 100% recovery	Stable 50 days; strong heat/light/UV protection; high encapsulation (96%)	3D printing (1‐mm nozzle); clear layers at ≥1%	Excellent astaxanthin retention; high bioaccessibility (∼48%); strong gel	SBP alone weak; excess EGCG (5:1) → larger particles	L. Zhang, Zhou, et al. 2023
Ultrafine soybean meal–corn oil HIPE	Smaller particles → denser interface + higher *φ* → stronger gels	Soybean meal 5%–10%; corn oil 70%–78%	Higher meal (%) ↓ droplet size; higher oil (%) ↑ packing	15,000 rpm, 5 min (*φ* = 80%); 5000 rpm, 5 min (70%–78%)	*G*ʹ >> *G*ʺ; viscosity↑ with meal% and oil%; shear thinning	Best at 10% meal and *φ* = 76%–78%; minimal delamination (21 days)	3D printing (Mickey head; FSE‐2 printer)	Optimal fidelity at 8% meal + *φ* = 76%	Too low viscosity (5%–6% meal, *φ* = 70%–72%) → collapse	Liao et al. 2024
DGSP–rapeseed oil W/O‐HIPPE (*φ* = 75%)	SP modifies diosgenin crystals → better wettability and adsorption	3% DGSP (ratios 3:1, 1:1, 1:3); water 75%	DGSP 1:1 → *θ* ≈ 106°, IFT 6.5 mN/m; small granular crystals form dense shell	Mix 1200 rpm → homogenize 12,000 rpm, 3 min	Shear‐thinning; *G*ʹ >> *G*ʺ; best recovery (78%) at 1:1	Strong F/T stability; small droplets (<20 µm); no delamination	3D printing (0.8‐mm nozzle)	High fidelity; self‐supporting; all‐natural particle	DGSP 1:3 inverts to O/W; diosgenin alone unstable	M. Wang, Zhou, et al. 2024
7S–KGM–soy oil HIPE (*φ* = 80%)	KGM enhances 7S adsorption + network strength	7S 2.5%; KGM 0%–0.625%	Best layer density + smallest droplets at 0.375% KGM	12,000 rpm, 180 s	Strong shear thinning; *G*ʹ >> *G*ʺ; best recovery and τ_0_ at 0.375%	Excellent thermal and oxidation stability; flocculation at high KGM	3D + 4D printing (color‐change CUR system)	High fidelity, self‐supporting; controlled 4D color response	High KGM (≥0.50%) → aggregation, weaker gels	Xie et al. 2025
SPI–CS–sunflower oil O/W HIPPE (*φ* ≈ 0.75)	CS electrostatic binding strengthens SPI adsorption + network	SPI 1%–5%; CS 0%–0.3%; oil 75%; curcumin encapsulated	SPI–CS complex ↓ droplet size; ↑ *ζ*‐potential; denser shells	12,000 rpm, 2 min	Shear‐thinning; *G*ʹ >> *G*ʺ; CS↑ → moduli↑, *τ*_0_↑	Stable 30 days; high curcumin retention; good heat/light stability	3D printing (0.6–1 mm nozzle)	High fidelity at SPI 3%–5% + CS 0.2%–0.3%; strong gels	SPI alone weak; too much CS → aggregation	Hou et al. 2025
7S–KGM–soybean oil HIPE (80%)	KGM enhances 7S adsorption + network strength	7S 2.5%; KGM 0%–0.625%; oil 80%	Best interfacial layer + smallest droplets at 0.375% KGM	Homogenization 12,000 rpm, 180 s	Strong shear thinning; *G*ʹ >> *G*ʺ; highest moduli and recovery at 0.375%	Excellent thermal and oxidative stability; high *φ* packing	3D/4D printing (color‐change curcumin system)	High fidelity; self‐supporting; pH‐responsive 4D features	High KGM (≥0.5%) → aggregation + reduced strength	H. Wang, Ouyang, et al. 2023
Glycinin amyloid fibril–soy oil HIPE	Longer fibrils → stronger interface + network	20 mg/mL fibrils (0–12 h heating); *φ* = 80%	Longer fibrils ↓*γ*, ↑*π*; dense elastic films (best at 11F9)	Ultrasonic + thermal fibrillation → homogenization	Shear‐thinning; *G*ʹ >> *G*ʺ; max moduli and recovery for 11F9	Stable 45 days; heat‐resistant	3D printing	High fidelity; strong gel; dense packing	Short fibrils weak; overheated (12 h) fibrils degrade	Y. Wang, Guo, et al. 2025
HIPE–rice starch gel (amylose‐dependent)	Amylose level tunes gel strength and printability	HIPE (*φ* = 75%) + rice starch gels (1.9%–18.9% amylose)	Amylose–protein interactions tighten packing; low amylose weak interface	HIPE prep → starch gelatinization → blending → extrusion printing	Shear‐thinning; *G*ʹ↑ with amylose; low amylose weak gels	High amylose → good integrity, OHC, bioactive protection	3D‐printed resveratrol/β‐carotene foods	Strong gels; high fidelity; protects actives	Low‐amylose systems collapse	L. Zheng, Li, et al. 2024
Microfluidic W/O/W HIPE	Microfluidics makes monodisperse, fat‐reduced HIPEs	W1: 0.6% gellan; O: palm/sunflower + lecithin; W2: OSA starch + gellan; 47%–62% fat	Gellan gel + oil crystallization form rigid shells	Capillary microfluidics; 60°C; controlled flow ratios	Shear‐thinning; *G*ʹ >> *G*ʺ; best mechanics at 54% fat	Stable 90 days; freeze–thaw stable; low oxidation	3D printing	Low‐fat, highly stable, printable	Too low (47%) or too high (62%) fat weakens printability	L. Guo et al. 2024
SC–soybean oil HIPE (*φ* = 0.80)	Hydrothermal starch–casein complex (H‐bonding) enhances adsorption and gel network	3% casein + 5% rice starch; heated 60°C–100°C; *φ* = 80%	100°C SC gives lowest IFT (∼24 mN/m), dense shell, small droplets	One‐step shear (8000 rpm, 2 min) after SC thermal treatment	Strong shear thinning; *G*ʹ >> *G*ʺ; highest moduli and recovery for 100°C	Stable 27–36 days (best at 4°C); tightly packed droplets	3D printing (1.2 mm nozzle; cylinder model)	Excellent fidelity, self‐supporting, strongest at 100°C	Casein alone fails; 60SC weak structure and poor printing	Y. Liu, Tan, et al. 2023
GOS‐GCP–flavor oil HIPE (*φ* = 80%)	Flavor–protein binding increases hydrophobicity → freeze–thaw stability	50 mg/mL GOS‐GCP; soybean oil + 10%–30% litsea cubeba oil	Higher *θ* (75° → 81°), lower IFT; stronger adsorption and denser interfacial layer ≥15% flavor oil	Two‐step shear: 8000 g, 90 s → acidification → 3000 g, 30 s	Shear‐thinning; *G*ʹ >> *G*ʺ; flexible gel network with high flavor content	Stable after five freeze–thaw cycles at ≥15% flavor oil; low oiling‐off	3D printing (1 mm nozzle); stable after freeze–thaw	Preserves volatile flavors; high resolution; good thixotropic recovery (>70%)	≤10% flavor oil → droplet coalescence and structure collapse after cycles	S. Hu, Xiao, et al. 2023
SPI–BC HIPE	BC + pH‐unfolded SPI strengthen interface and gel	SPI 20 mg/mL; BC; pH 2/12; oil	Lower *γ* and *θ*; ↑ hydrophobicity; dense SPI–BC film	pH‐shift treat SPI → mix BC → homogenize	Shear‐thinning; *G*ʹ >> *G*ʺ; strongest at BC + pH 2	Best freeze–thaw stability; small droplets	3D printing	Strong gels; high fidelity	SPI alone weak; pH 12 less stable	R. Zhao et al. 2025
CSPP–palm olein HIPPE (80%)	CS–PP complex + TPP crosslinking strengthen interface and gel	0.1% CS; 1%–3% PP; *φ* = 80%	Smaller droplets; stronger adsorption; TPP reinforces CS–PP network	Two‐step homogenization	Shear‐thinning; *G*ʹ >> *G*ʺ; CSPP1:150 best printing	3 days (25°C)/10 days (4°C); TPP extends stability	3D printing	High fidelity; improved storage	Low PP ratios unstable; CSPP1:150 low thixotropic recovery	Karandagaspitiya et al. 2025
OG–XG HIPE (*φ* = 80%)	XG enhances OG adsorption and gel network	OG 1%–5%; XG 0%–0.5%; oil 80%	Smaller droplets; stronger interface, best at 4% OG + 0.5% XG	High‐shear emulsification	Shear‐thinning; *G*ʹ >> *G*ʺ; moduli ↑ with OG/XG	Stable 7 days; reduced coalescence	3D printing	High fidelity; smooth extrusion	OG alone weak; excess XG too viscous	Luo et al. 2025
PPH NP–HIPPE (O/W)	Natural polysaccharide–protein NPs form dense multilayer barrier	1.5% PPH NPs; isopropyl myristate *φ* = 0.8	Multilayer adsorption; steric + electrostatic stabilization; small droplets (∼9–12 µm)	Homogenization 11,000 rpm, 24 s	Strong shear thinning; *G*ʹ >> *G*ʺ; viscosity ↑ with NP %	Stable to centrifuge (10,000 × *g*), pH 3–11, 0–500 mM salt, 4°C–121°C; re‐forms after freeze–thaw	3D printing (0.2 mm nozzle)	Excellent shape fidelity; stable 14 days; minimal oiling‐off	*φ* = 0.9 unstable; −20°C causes temporary breakdown	D. Li, Yin, et al. 2023
Casein/Soy/Pea/WPI HIPE (80%)	Post‐acidification induces protein aggregation → stronger gel	5% protein; *φ* = 80%; pH shifted 10 → 2–6	IFT stays low; interfacial layer unchanged; bulk network strengthened	Make HIPE at pH 10 → add HCl	Shear‐thinning; *G*ʹ↑; fast recovery	Stable droplets; no oiling‐off	3D printing	Universal strategy; high fidelity	Too much aggregation reduces smoothness	Xiao et al. 2024
β‐CD or β‐CD–CS HIPE (*φ* = 75%)	CS + β‐CD strengthen interfacial network, improve CUR protection	β‐CD 1.2%; CS 0.32%; sunflower oil + CUR	Denser β‐CD/CS layer; ↑ steric and electrostatic stabilization	12,000 rpm, 3 min	Shear‐thinning; *G*ʹ > *G*ʺ; strongest at pH 5 with CS	Stable 20 days; heat‐stable at pH 3–6; pH 7 unstable	3D printing	High CUR stability, fidelity, bioaccessibility	β‐CD alone weak; heat >85°C breaks structure	X. Li et al. 2023b
OSA starch–XG HIPE (*φ* = 75%)	XG thickens continuous phase, enhances gel and printability	1% OSA; 0%–0.4% XG; *φ* = 75%	XG ↑ viscosity → ↓ droplet collision; smaller droplets (0.1%–0.3% XG)	One‐pot mixing + 23,000 rpm, 2 min	Shear‐thinning; *G*ʹ > *G*ʺ; *τ*_0_ ↑ (175 → 433 Pa) with XG	Stable 30 days at 0.1%–0.3% XG; instability at 0% and 0.4% XG	3D printing (0.84 mm nozzle)	Best fidelity at 0.3% XG; strong recovery; clear layers	0% XG collapses; 0.4% XG too viscous → poor emulsification	Teng et al. 2025
Arg–MMP HIPPE (*φ* = 75%)	Arg unfolds myosin → microgel with ↑ amphiphilicity	Myosin 20 mg/mL; Arg 0%–3%; 75% corn oil	Smaller droplets (∼20 µm); denser adsorption layer; ↑ hydrophobicity and ‐SH	Heat (90°C) → microgelization → homogenization	Shear‐thinning; *G*ʹ > *G*ʺ; max strength at 1% Arg; >70% recovery	Dense packing; stable structure; improved deformation resistance	Low‐salt surimi 3D printing	High stability; strong gels; good self‐supporting layers	Excess Arg (≥3%) causes aggregation; 0% poor printability	Q. Lu et al. 2025
Pea protein microgel HIPPE (*φ* = 75%)	Heat‐formed microgels strengthen interface and gel	PPM (60°C–100°C); *φ* = 75%	Strong adsorption; smaller droplets; thicker shell	Hydration → thermal gelation → high‐shear	Shear‐thinning; *G*ʹ >> *G*ʺ; best at 100°C	High stability; low coalescence	3D printing	High fidelity; protein‐dense ink	Low‐T PPM gives weak gels	Zhou et al. 2025
OCD1–SH HIPE (*φ* = 75%)	Non‐electrostatic OCD1–SH assembly strengthens interface	3% OCD1; 0.125–1.25% SH; soybean oil 75%	Hydrophobic insertion + H‐bonding; ↓IFT (∼15 mN/m); thicker layer	Homogenization 13,000 rpm, 3 min	Shear‐thinning; *G*ʹ >> *G*ʺ; strongest at ∼0.5% SH	pH 3–11 stable; salt strengthens; heat ≥60°C unstable	3D printing	High fidelity; tunable strength	Excess SH → aggregation; heat‐sensitive	S. Li, Wei, et al. 2025
CRP/XG HIPPE (*φ* = 75%)	CRP–XG crosslinked network strengthens interface and gel	3% CRP; XG 0.4%–1.5%; soybean oil 75% + lutein	H‐bonding + hydrophobic interaction; denser shell; ↓IFT; thicker coating	9000 rpm, 3 min	Shear‐thinning; *G*ʹ > *G*ʺ; strongest at XG‐1.5	Excellent storage, thermal (≤95°C), freeze–thaw, pH 3–9, and salt stability	3D printing (“H” and “D” shapes)	High lutein EE (92%); high residual lutein (89%); ↑FFA release and bioaccessibility (52%)	CRP alone unstable; alkaline pH (11–13) weak; low‐XG gels soft	R. Liu, Liu, et al. 2025
Lyc‐YP HIPPE (*φ* = 85%	Lyc enhances YP interfacial film + antioxidant function	2.5 mg/mL YP; canola oil + 0–1.5 mg/mL Lyc	Lyc adsorbs on YP → thicker shell; large droplets at high Lyc	13,000 rpm, two 30‐s intervals	Shear‐thinning; gel‐like	High EE; strong antioxidant effect	3D‐printed meat fat replacer	High fidelity; lower oxidation; good yield	High Lyc increases droplet size, alters texture	Cao et al. 2025
SSOS/XG HIPPE (*φ* = 74%)	XG + SSOS synergistic network → stronger gel, better protection	10% SSOS; 0%–0.6% XG; MCT oil 74% + β‐carotene	H‐bonding; thicker interfacial layer; improved wettability; steric–electrostatic stabilization	15,000 rpm, 6 min	Shear‐thinning; *G*ʹ > *G*ʺ; *τ*_0_ ↑ with XG; good recovery	6‐month retention up to 32% (0.6% XG); smaller droplets; reduced coalescence	3D printing (cylinders, clover, cherry blossom)	Near‐100% printing accuracy at 0.6% XG; higher β‐carotene bioaccessibility (up to 21%)	Too much XG lowers bioaccessibility (6% at 0.6%); SSOS alone unstable	An et al. 2025
SPH–CLEO HIPPE (*φ* = 80%)	SPH peptides improve CLEO dispersion + antioxidant/color stability	SPH (5%–7% w/v); CLEO 5%; *φ* = 0.8	SPH adsorbs strongly; smaller droplets (↓ with SPH↑); enhanced film elasticity	Homogenization 10,000 rpm, 3 min	Shear‐thinning; *G*ʹ > *G*ʺ; viscosity ↑ with SPH; good recovery	Stable color (L*, a*, b* retention); DPPH radical inhibition ↑; storage‐stable	3D‐printed lattices and cubes	Strong antioxidant and color retention; smooth extrusion; high fidelity	Low SPH → weak gels; high SPH → overly viscous	Lan et al. 2025
LF–QU HIPE	LF–QU complexes enhance interface + structure	LF (0.4–0.8 wt%), QU, rapeseed oil (80%), aqueous phase	H‐bonding, hydrophobic and electrostatic LF–QU interactions; strong adsorption; *θ* → 90° at 0.8 wt%	High‐speed homogenization (10,000 rpm); O/W HIPE formation	Shear‐thinning; *G*ʹ > *G*ʺ; viscoelasticity ↑ with LF; tight droplet packing	Stable 30 days; low MDA/LPO; minimal phase separation at 0.8 wt%; small droplets	3D printing of gel‐like edible structures with high fidelity	High encapsulation (>90%); improved QU bioaccessibility (44%); strong antioxidant protection	Requires high LF (≥0.7 wt%) for stability; low LF → flocculation and collapse	Y. Huang et al. 2025
QPI–TA–HMP HIPE (*φ* = 75%–80%)	Ternary complex (protein–polyphenol–pectin) strengthens interfacial layer and gel network	1% QPI; 0.05% TA; 0.125%–1% HMP; soybean oil 75%–80%	H‐bonding, hydrophobic and electrostatic interactions; smaller droplets; dense coating	10,000 rpm, 2 min	Strong shear thinning; *G*ʹ >> *G*ʺ; gel strength ↑ with HMP	QTH3 (1:0.05:1) most stable; small droplets; strong gel; oil leakage ↓	3D printing (“Superman,” turtle, rabbit)	High fidelity at high HMP; fast recovery; compact network	QT alone unstable; low HMP collapses; very acidic pH causes aggregation	Qu et al. 2025
SPI/BC co‐assembly HIPE (*φ* = 75%)	pH‐shift–induced SPI unfolding + BC entanglement → co‐assemblies	2% SPI/BC (30:1 or 15:1); 75% soybean oil	Unfolded SPI exposes SH and hydrophobic groups; BC forms 3D network; dense uniform interfacial layer	12,000 rpm, 2 min homogenization; pH shift 7 → 2 → 7	Strong shear thinning; *G*ʹ > *G*ʺ; LVR wider with higher BC; co‐assemblies show highest *G*ʹ stability	Smallest droplet size (15.6 µm); minimal change over 28 days; no oil separation; best heat stability (90°C, 30 min)	3D printing (0.8 mm nozzle, 22 mm/s)	High fidelity; no collapse after 48 h; strongest gel (break force ∼102 g)	Requires acid pH shift; mixtures (no pH shift) show phase separation	C. Wang, Zhao, et al. 2025
CPCNP–TA, CPCNP–SA, CPCNP–XG HIPEs (*φ* = 0.75)	Binary complexes strengthen adsorption and viscoelasticity	1% CPCNPs + TA/SA/XG (0.1 g per 2 g CPCNPs); MCT oil 75%	H‐bonding + electrostatic interactions; XG gives highest adsorption, lowest *γ*, thickest interfacial layer	13,500 rpm, 60 s homogenization	Shear‐thinning; *G*ʹ > *G*ʺ; strongest gel: CPCNP‐XG	High oxidative stability; XG lowest LH/MDA; stable 28 days; best centrifugal stability	3D printing (flower model)	High fidelity; strongest recovery (3ITT); anthocyanins further improve structure	More complex prep; TA increases aggregation; CPCNP alone weak	J. Wang, Wang, et al. 2025
FA–SPI‐stabilized HIPE	FA–SPI interactions (H‐bond + hydrophobic) enhance interfacial strength	SPI + ferulic acid (FA); optimal ratio SPI:FA = 6:1	FA increases β‐sheets, hydrophobicity; ↓β‐turn; stronger adsorption	Prepare FA–SPI complex → emulsification	Shear‐thinning; *G*ʹ >> *G*ʺ; viscosity and elasticity highest at 6:1	Improved WHC; tighter network; good storage stability	3D printing of FA–SPI HIPE gels	Best print fidelity at 6:1; high antioxidant activity; stable structure	Too much FA → over‐elasticity, poorer extrusion	L. Liu, Yang, et al. 2025

Abbreviations: BCNF, bacterial cellulose nanofiber; CHC, chitosan hydrochloride; CNF, cellulose nanofiber; CP, collagen peptide; DGSP, diosgenin–soy protein complex; DSC, differential scanning calorimetry; EAI, emulsifying activity index; EGCG, epigallocatechin gallate; ESI, emulsion stability index; FA, ferulic acid; FFA, free fatty acid; FP, fish protein; FSG, fish sarcoplasmic protein gel; GMP, glycomacropeptide; HMP, high‐methoxyl pectin; JP, jackfruit protein; KGM, konjac glucomannan; LF, lactoferrin; LM, liquid metal; LVR, linear viscoelastic region; MDA, malondialdehyde; MOF, metal–organic framework; m‐SiO_2_, mesoporous silica; MTGase, microbial transglutaminase; NCh, nanochitin; O/W, oil‐in‐water; ODSA, octadecyl succinic anhydride; OHC, oil‐holding capacity; OMS, octenyl‐modified starch; PC, polyphenol complex; PEG, polyethylene glycol; POV, peroxide value; PPC, protein–polysaccharide complex; PVA, poly(vinyl alcohol); SC, sodium caseinate; TGase, transglutaminase; TOCN, TEMPO‐oxidized cellulose nanofiber; TSI, Turbiscan stability index; WHC, water‐holding capacity; WPI, whey protein isolate; WPIA, whey protein isolate amyloid; ε‐PL, ε‐poly‐L‐lysine.

HIPPEs are not formed solely by increasing oil content, but rather by satisfying a set of coupled physicochemical requirements that collectively stabilize highly concentrated droplet assemblies. First, the dispersed‐phase volume fraction must typically exceed the random close‐packing threshold (*φ* ≈ 0.74), forcing droplets into direct contact and generating mechanical jamming (X. He and Lu 2024). Second, stabilizing particles must possess intermediate wettability, generally corresponding to contact angles near 90°, which maximizes attachment energy and minimizes particle desorption from the oil–water interface (Binks 2002; Durgut and Claeyssens 2025). Third, particle concentration must be sufficient to achieve extensive interfacial coverage, since incomplete coverage promotes droplet coalescence and catastrophic phase separation under the high capillary stresses associated with concentrated emulsions. Fourth, interfacial films must exhibit adequate mechanical strength and elasticity to accommodate droplet deformation while resisting rupture during processing and storage. Finally, the continuous phase must provide sufficient viscosity to suppress droplet mobility without excessively restricting extrusion flow. Successful HIPPE formation, therefore, emerges from a balance between interfacial adsorption, particle wettability, droplet packing, and continuous‐phase rheology rather than from any single formulation parameter alone.

The oil fraction further constrains stabilization requirements. HIPPEs formulated at *φ* = 0.75–0.77 typically tolerate lower particle coverage, whereas systems at *φ* ≥ 0.80 require enhanced interfacial reinforcement. These indicate that stabilization is governed by the combined effects of internal phase fraction and interfacial packing density, linking composition directly to the rheological envelope discussed in Section 3.3.

Collectively, HIPPE stabilization can be understood as the coupled outcome of five governing factors: (i) *φ* ≥ 0.74 to induce droplet jamming, (ii) intermediate particle wettability to maximize adsorption energy, (iii) sufficient interfacial particle coverage to prevent coalescence, (iv) mechanically robust interfacial films capable of resisting deformation, and (v) continuous‐phase rheology that supports network integrity while preserving processability. Variations in particle chemistry, morphology, protein–polysaccharide interactions, and processing conditions influence HIPPE performance primarily through their effects on these stabilization mechanisms.

### 3.2 Particle–Interface Interactions

**FIGURE 3:**
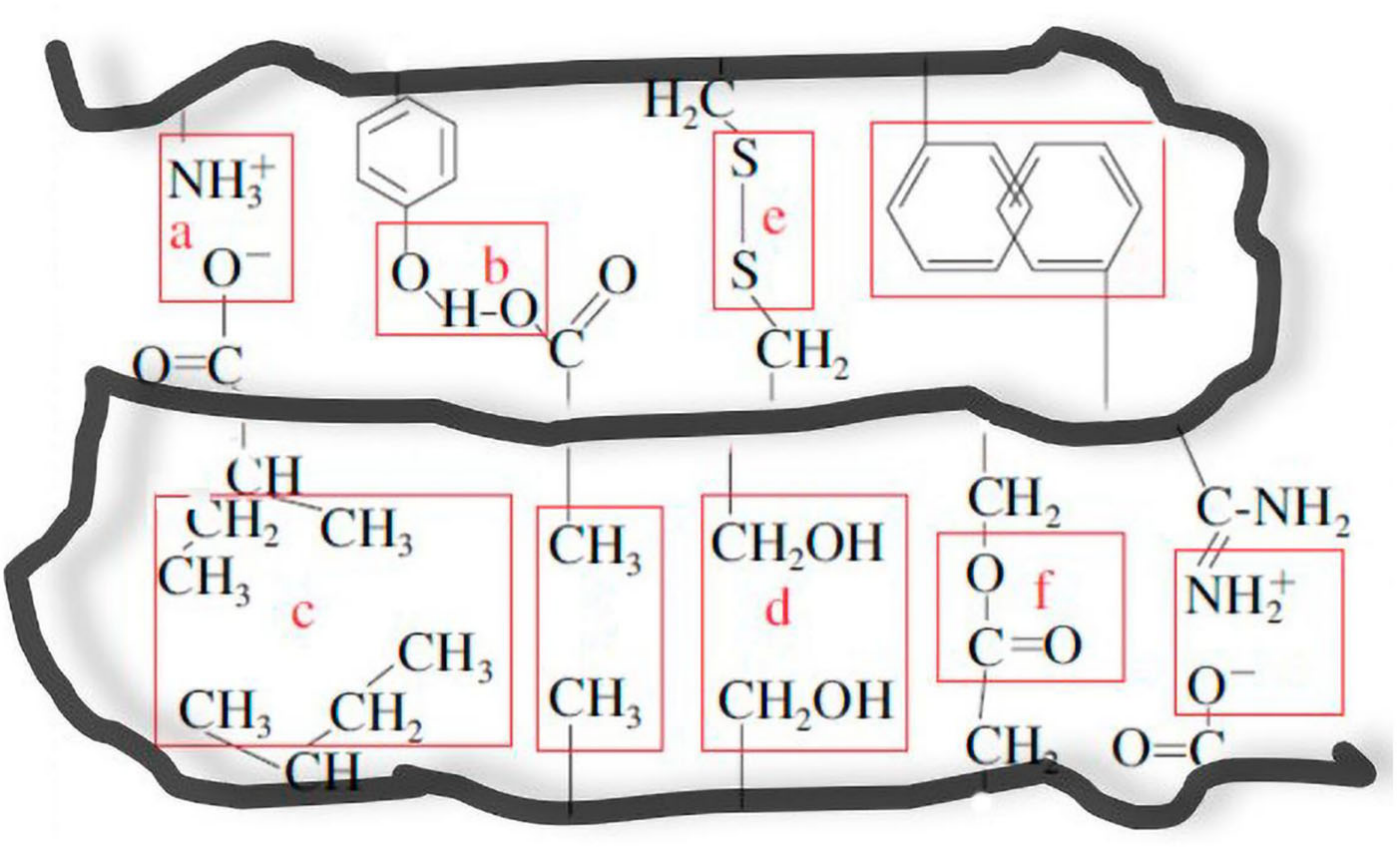
Schematic representation of potential molecular functional groups and dominant intermolecular and covalent interactions: (a) ionic interactions, (b) hydrogen bonding, (c) hydrophobic interactions, (d) van der Waals interactions, (e) disulfide covalent bonds, and (f) ester covalent linkages, adapted from M. Zhang, Li, et al. (2023).

At high internal phase fractions, droplet deformation increases capillary pressure, requiring interfacial particle layers to reorganize without desorption to preserve stability. Systems employing anisotropic particles consistently exhibit enhanced resistance to this stress. In a study, starch nanocrystal–stabilized HIPPEs show storage moduli (*G*ʹ) in the range of 1–10 kPa and retain structural integrity at *φ* ≥ 0.80, whereas similar spherical particle systems display *G*ʹ values below 500 Pa and partial oil leakage under shear (Shahbazi, Jäger, et al. 2024; Shuang et al. 2024). Similar behavior occurs in chitin nanowhisker HIPPEs, where rod‐like particles form percolated interfacial networks that suppress coalescence for up to 90 days at *φ* = 0.8 (Shuang et al. 2024). Mechanistically, anisotropic particles exhibit enhanced resistance to interfacial stress because their elongated morphology increases the particle–interface contact area and adsorption energy while promoting lateral particle–particle interactions. This results in interconnected interfacial networks that distribute capillary and shear stresses more efficiently than spherical particles, thereby reducing particle desorption, droplet coalescence, and interfacial failure under deformation (Berton‐Carabin and Schroën 2015; Binks 2002). At the molecular level, interfacial assembly of biopolymer‐based Pickering stabilizers is governed by a combination of noncovalent interactions (hydrogen bonding, hydrophobic interactions, van der Waals forces, and electrostatic/ionic interactions) and, in selected systems, covalent linkages such as oxidative crosslinking or ester bonds formed via thermal treatment, enzymatic crosslinking, or polyphenol‐mediated reactions, which together determine adsorption irreversibility and interfacial elasticity (Figure 3).

Particle surface modification and intermolecular complexation influence particle–interface interactions by altering wettability, adsorption energy, interfacial packing density, and adsorption kinetics, thereby affecting the stability and mechanical strength of HIPPE interfaces. Surface modification or intermolecular complexation can enhance interfacial anchoring by exposing hydrophobic domains, strengthening intermolecular interactions, or increasing particle deformability, thereby promoting the formation of more cohesive and elastic interfacial films. Protein–polyphenol systems primarily represent intermolecular complexation rather than strict covalent particle modification. Soy protein isolate (SPI)–tannic acid (TA) HIPPEs exhibit increased interfacial adsorption (from ∼64% to ∼75%) and reduced droplet diameters (from ∼35 to ∼9 µm) at moderate TA levels, leading to *τ*_0_ over 150 Pa and improved extrusion continuity (W. Hu, Chen, et al. 2023). In whey protein–polyphenol systems, increasing polyphenol concentration reduces interfacial tension and raises viscosity at 0.1 s^−1^ from ∼1200 to >2000 Pa·s, correlating with enhanced layer fidelity (Ji, Sun, et al. 2025). Collectively, these examples demonstrate that particle modification primarily affects HIPPE performance through its influence on particle–interface interactions, including adsorption behavior, interfacial film formation, and interfacial mechanical stability, which subsequently govern bulk rheological behavior.

### 3.3 Rheological and Interfacial Properties Relevant to Bioink Performance

Printable HIPPE‐based bioinks consistently exhibit a finite *τ*_0_, strong shear‐thinning behavior (with flow behavior index *n* typically ranging from 0.2 to 0.4), and rapid recovery after shear. Table 1 shows that *τ*_0_ below ∼50 Pa often results in filament spreading, whereas systems with *τ*_0_ ≥ 100–300 Pa maintain filament integrity and layer stacking. For example, phosphorylated perilla protein–chitosan (CS) HIPPEs show an increase in *τ*_0_ around 24 Pa to about 160 Pa as CS content increases, accompanied by a rise in viscosity recovery from ∼70% to >90% and improved shape fidelity (Q. Zhao, Fan, et al. 2023). Comparable trends are observed in gelatin–oleofoam HIPPEs, where increasing gelatin concentration elevates *G*ʹ from approximately 2.5 to 9.8 kPa and enables self‐supporting extrusion (X. He and Lu 2024).

Interfacial properties strongly govern these rheological responses. Systems with lower interfacial tension and high particle surface coverage display *G*ʹ >> loss modulus (*G*ʺ) over broad frequency ranges and recovery ratios exceeding 80%–95% in three‐interval thixotropy tests (3ITTs). All β‐CD, cellulose nanocrystal (CNC)‐stabilized, and protein fibril–stabilized HIPPEs fall within this regime, whereas formulations with incomplete interfacial coverage show delayed recovery and layer fusion (K. He, Sheng, et al. 2024; X. Li et al. 2023a). These trends directly connect interfacial assembly (Section 3.2) to extrusion performance.

Beyond static oscillatory rheology, several studies demonstrate that printability correlates more robustly with coupled interfacial adsorption kinetics and nonlinear viscoelastic descriptors than with *τ*_0_ alone. For instance, in MP–trehalose HIPPEs (*φ* = 0.80), interfacial diffusion (*K*_diff_ 0.125–0.194 mN·m^−1^·s^−0.5^) and penetration/rearrangement constants (*K*_p_ 2.23 × 10^4^ → 3.44 × 10^4^ s^−1^; *K*_r_ 8.63 × 10^4^ → 10.22 × 10^4^ s^−1^) increased at 10% trehalose, concomitant with droplet size reduction (67.6 → 25.1 µm), *G*ʹ >> *G*ʺ dominance, and ∼93% 3ITT, enabling filament integrity above 90% printing accuracy (Hong et al. 2025). Similarly, heat‐set soy protein isolate (HSPI)–carrageenan (CG) complexes (1% HSPI, 0.2%–0.8% CG) showed progressive surface pressure (π) increases and droplet contraction (26.3 → 13.5 µm) with rising CG, while *G*ʹ and apparent viscosity increased systematically, producing stable cylinders only when CG ≥0.4% (R. Li, Guo, et al. 2025). These examples demonstrate that interfacial adsorption parameters, including diffusion and rearrangement kinetics, are closely associated with droplet microstructure, viscoelastic behavior, and printing performance. Integration of such interfacial kinetic descriptors with bulk rheological measurements may improve mechanistic understanding of formulation–printability relationships in HIPPE‐based bioinks.

### 3.4 Formulation Parameters Controlling Internal Architecture

Internal architecture in HIPPEs is dictated by the coupled effects of internal phase fraction, particle concentration, and particle geometry. Increasing *φ* from ∼0.75 to above 0.80 enhances droplet crowding and elasticity but narrows the processing window because the reduced ability of droplets to rearrange under stress can promote brittle failure if interfacial reinforcement is insufficient. Consequently, higher *φ* systems generally require greater particle coverage and stronger interfacial networks to maintain structural integrity during extrusion. Particle geometry also strongly influences internal architecture. Anisotropic particles such as fibrils, nanowhiskers, and nanocrystals can form interconnected interfacial networks and inter‐droplet bridges that distribute stresses more effectively than spherical particles. As a result, anisotropic stabilizers often permit stable HIPPE formation at higher *φ* values while maintaining elasticity, structural cohesion, and printing fidelity (Shahbazi, Jäger, et al. 2024; Zong et al. 2022).

An investigation of SPI microgel HIPPEs shows viscosity increases from around 1100 to 2400 Pa·s when oil fraction rises from 40% to 60%, while stability declines at higher oil contents without additional interfacial reinforcement (Wu et al. 2024). Particle concentration exhibits a nonmonotonic effect. In octenyl succinic anhydride (OSA) starch–citrus fiber systems, increasing fiber content up to 0.3 wt% improves shape accuracy to ∼98%, whereas further increases to 0.4 wt% cause swelling and extrusion defects due to excessive continuous‐phase structuring (Wei et al. 2025). Similar over‐stiffening effects are reported for xanthan gum (XG)‐rich systems, where high viscosity impedes droplet rearrangement despite enhanced stability (Teng et al. 2025). Oil viscosity and polarity further influence droplet deformation and stress distribution, impacting extrusion smoothness without directly altering interfacial stabilization mechanisms (F. Yang et al. 2024). Collectively, the studies reviewed indicate that successful HIPPE formation is governed by a narrow balance among internal phase fraction, particle wettability, interfacial coverage, and network connectivity. While increasing *φ* and particle concentration generally enhances elasticity and shape retention, excessive reinforcement often reduces extrudability and promotes brittle behavior. Anisotropic and fibrillar stabilizers appear particularly advantageous because they achieve comparable mechanical performance at lower particle concentrations than spherical particles. However, systematic comparisons under standardized processing conditions remain scarce, limiting the establishment of universal formulation criteria.

## 4 HIPPEs as Structurally Tunable Bioinks

### 4.1 Structural Modulation Through Droplet Size, Particle Wettability, and Phase Ratios

Structural tunability in HIPPE bioinks is governed by the coupled control of droplet size distribution, interfacial particle affinity, and phase ratio because these variables determine (i) interfacial area per unit volume, (ii) the degree of droplet deformation/jamming, and (iii) the connectivity of the percolated droplet network under printing‐relevant stresses (H. Chen, Yu, et al. 2023). In practice, reducing droplet size at fixed *φ* increases the particle coverage requirement but yields mechanically sharper interfaces and improved dimensional accuracy; OSA–potato starch/citrus insoluble fiber ingredient (CIFI) HIPPEs reduced droplet sizes from ∼16 to 7 µm (CF03) and ∼7 to 3 µm (CM1120) while increasing *τ*_0_ from 10 to 131 Pa and 33 to 218 Pa, respectively, which translated into higher printed shape accuracy (up to ∼97.7%) and reduced side‐area loss over 96 h (from 47% to 19% for CF03; 23% to 13% for CM1120) (Wei et al. 2025). At a similar *φ* (∼0.75), protein–polysaccharide complexation achieves a comparable structural shift by strengthening droplet interfacial coverage and the surrounding aqueous network rather than simply increasing particle loading. In pea protein isolate (PPI)/Konjac glucomannan (KG) HIPPEs, increasing KG reduced droplet size from 7.90 to 6.47 µm, decreased water mobility according to LF‐NMR, and increased *τ*_0_ from ∼200 to ∼897 Pa, enabling stable printed gels only above the KG threshold where network strength became sufficient (Z. Liu et al. 2024).

**FIGURE 4:**
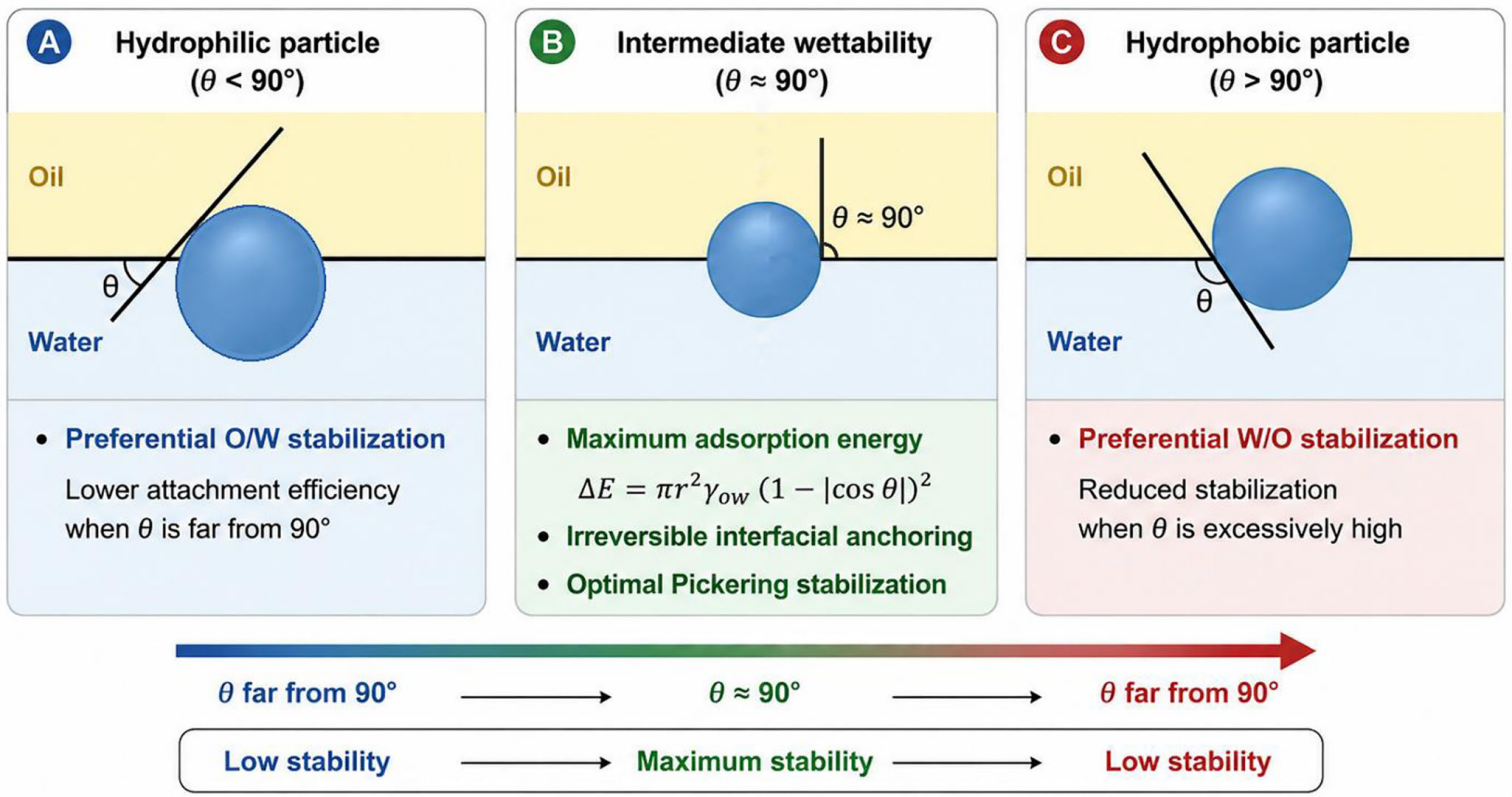
Schematic illustration of particle wettability‐contact‐angle‐controlled Pickering stabilization in HIPPE‐based bioinks. Particles with intermediate wettability adsorb irreversibly at the oil–water interface, enabling stable oil‐in‐water (*θ* < 90°) or water‐in‐oil (*θ* > 90°) HIPPE structures.

Wettability tuning determines whether particles remain trapped at the interface during extrusion‐induced deformation or detach and allow coalescence (Figure 4); this appears as abrupt changes in droplet size stability and recoverable elasticity rather than gradual trends (S. Lu et al. 2024). Hydrophobically modified solid stabilizers clearly demonstrate this. Silylated CNC HIPPEs were printable across a wide range of aqueous conditions (pH 3–12; NaCl 0–1 M) at extremely low stabilizer contents (≥0.06 wt%) while still exhibiting strong shear thinning (viscosity [*η*] ∼10^3^ → 10^1^ Pa·s from 0.01 to 100 s^−1^) and rapid recovery (∼80%–90%), indicating that contact‐angle tuning can substitute for high stabilizer loading by increasing adsorption irreversibility (X. He et al. 2022). Phase ratio primarily governs the transition from viscoelastic paste to jammed solid. In SPI microgel systems, increasing the oil fraction from 40% up to 60% increased viscosity from ∼1143 to ∼2421 Pa·s and strengthened printed accuracy (reported best at the highest oil fraction), but at the expense of higher flocculation/oil separation risk, indicating a *φ*‐dependent trade‐off between stiffness and inter‐droplet mobility that directly affects filament continuity (Wu et al. 2024). These combined mechanisms set the “structural design space” that later underpins time‐dependent shape programming in multidimensional printing (Sections 4.2–4.4).

### 4.2 Rheological Characteristics Relevant to Printability

**FIGURE 5:**
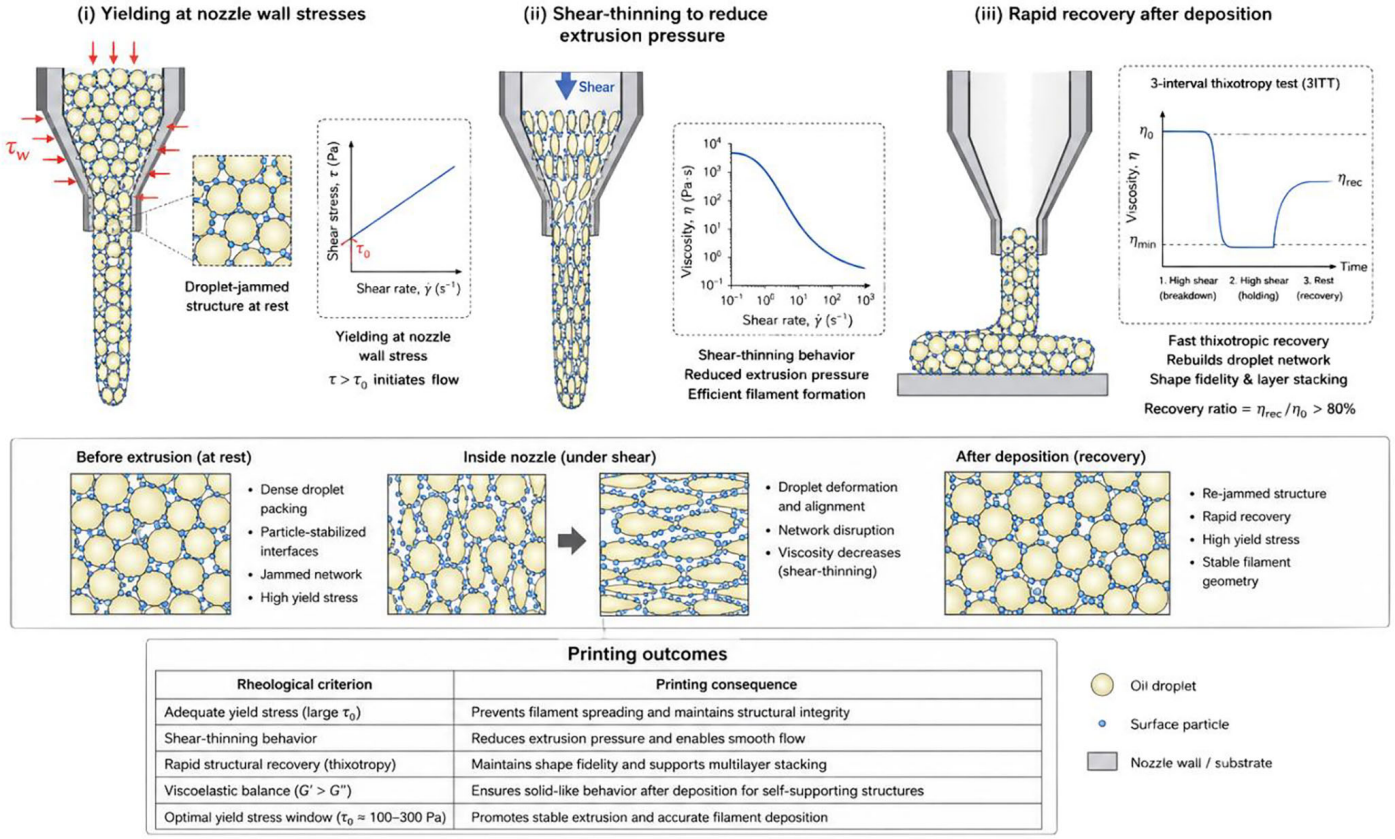
Rheological criteria governing printability of HIPPE‐based bioinks in extrusion‐based printing.

Printability emerges when the ink simultaneously satisfies three mechanical constraints: (i) yielding at nozzle wall stresses, (ii) shear thinning to reduce extrusion pressure, and (iii) rapid recovery to rebuild the droplet network before gravitational or capillary force distorts the deposited filament (Z. Wang, Huang, et al. 2023). Systems that achieve this balance often show large *τ*_0_ while maintaining recoverable thixotropy (Figure 5). Egg yolk/carboxymethyl cellulose (CMC) HIPPEs provide a clear example: increasing CMC shifted droplet size from 34.6 to 8.9 µm and raised viscosity at 0.1 s^−1^ from ∼99 to ∼769 Pa·s. Further, *τ*_0_ increased from ∼24 to ∼91 Pa. The formulation that printed best (YC‐7.5) combined high viscosity with recoverable structure such that complex features (ears/fins) remained intact after deposition (Hou et al. 2024). A distinct route to the same mechanical target is ion‐mediated network consolidation. Gelatin HIPPEs strengthened by sodium citrate (Na_3_Cit) showed extremely strong elastic dominance (*K*′ ∼1782 Pa, *n*ʹ ∼0.029) and high retention of functional payloads at elevated temperatures, indicating that Hofmeister‐driven junction strengthening can elevate solid‐like response without requiring very small droplets (L. Zheng, Li, Wang, et al. 2025).

Interfacial mechanics can dominate bulk viscoelasticity when the interfacial film becomes thick/rigid relative to droplet size, which increases *G*ʹ and suppresses filament necking. Covalently anchored interfacial systems show this most strongly; the aldehyde‐modified alginate (Ugi reaction precursor) (Ugi‐OA)/aminated silica HIPPE achieved a significant viscosity amplification (∼100×) upon aminated silica nanoparticle (ASN) addition and exhibited gel‐like elasticity (*G*ʹ > 100 Pa across 10–1000 rad/s), enabling printing of fine symbols and multilayer motifs at low nanoparticle concentrations (Z. Wang, Huang, et al. 2023). In contrast, systems where viscosity is raised primarily through bulk thickening can reach high fidelity but are limited by flow instabilities at high solids. Gum Arabic (GA) HIPPEs achieved stable printing only near the optimal GA concentration (6 wt%), where *G*ʹ exceeded approximately 10^3^ Pa and recovery reached about 85%–90%, while further viscosity increases reduced flowability and compromised complex deposition (Liang et al. 2024). These examples show that equivalent printability can arise from different mechanical architectures (interfacial rigidity vs. continuous‐phase thickening), which becomes critical when biocompatibility and diffusion constraints are introduced (Section 4.4). Although interfacial particle networks are the primary source of elasticity in HIPPEs, the continuous phase and oil phase also contribute to the overall rheological response. Continuous‐phase polymers can increase viscosity, promote droplet bridging, and improve structural recovery, whereas oil viscosity influences droplet deformability, lubrication, and stress transmission during extrusion. Consequently, printability emerges from the combined contributions of interfacial mechanics, continuous‐phase structuring, and dispersed‐phase properties rather than from any single component alone.

A complementary strategy involves maximizing interfacial connectivity per unit particle mass rather than increasing total solids. Pea‐protein fibril HIPPEs (1–5 wt%) demonstrate this principle; fibrillation reduced droplet size from 32.6 to 12.4 µm and elevated *G*ʹ from 1.2 to 4.8 kPa, while maintaining ∼85% thixotropic recovery and storage stability for ≥30 days (Shahbazi et al. 2021). Comparable efficiency is observed in hydrophobically modified cellulose nanocrystal (H‐CNC) systems, where as little as 0.06 wt% hydrophobized CNC stabilized *φ* up to 0.83, yielding strong shear thinning (10^3 ^→ 10^1^ Pa·s) and rapid recovery (∼80%–90%) across pH 3–12 and 0–1 M NaCl (X. He et al. 2022). In contrast, spherical particle systems often require >3 wt% solids to achieve equivalent *τ*_0_ values. This comparison indicates that anisotropic morphology and hydrophobic surface tuning shift the percolation threshold of interfacial networks, expanding the printable composition window while preserving low continuous‐phase viscosity. Such mass efficiency is particularly relevant for biomedical formulations where excess particle load may compromise cytocompatibility.

### 4.3 Encapsulation and Biological Functionality

HIPPE bioinks provide intrinsic compartmentalization for hydrophobic payloads while allowing rheology control through interface design, so encapsulation and printability can be tuned simultaneously rather than sequentially (D. Zhang, Yang, et al. 2024). Drug delivery scaffolds derived from water‐in‐oil (W/O) HIPEs demonstrate high encapsulation efficiency (EE) and controlled release driven by pore architecture formed during solidification. In food‐grade HIPPE systems, interfacial engineering can substantially improve the retention and gastrointestinal bioaccessibility of lipophilic compounds. For example, gliadin–CMC HIPPEs increased β‐carotene retention to approximately 42.9% and enhanced bioaccessibility from about 12.3% to 37.0%. D. Zhang, Yang, et al. (2024) attributed this improvement to gliadin–CMC complexation, which reduced droplet size, strengthened the interfacial barrier surrounding oil droplets, and improved structural recovery (∼93%), thereby enhancing protection of β‐carotene during processing and digestion while maintaining excellent printability. From a food science and safety perspective, the selection of stabilizing particles and continuous‐phase components is equally important as rheological performance and printability. Although many HIPPE systems are formulated using food‐grade proteins, polysaccharides, and biopolymeric particles, increasing structural complexity and interfacial engineering may introduce additional toxicological and regulatory considerations, particularly when chemically modified particles, reactive crosslinking systems, or nanostructured stabilizers are employed (Rehman et al. 2024; Wu et al. 2022). Moreover, the highly porous and high‐moisture characteristics of many extrusion‐printed HIPPE foods may accelerate oxygen diffusion, lipid oxidation, moisture migration, and microbial proliferation. As a result, shelf‐life stability, spoilage kinetics, and contamination susceptibility may be affected during post‐print handling, packaging, and storage, particularly in high‐moisture systems with elevated water activity (X. He and Lu 2024; Tyowua et al. 2024). Consequently, future food‐grade HIPPE systems should be evaluated not only for EE and printing fidelity, but also for digestion behavior, oxidative stability, microbiological safety, allergenicity, and long‐term storage performance under realistic food‐processing conditions.

Encapsulation goes beyond passive retention; it can also be linked to structural transitions triggered after printing. In microfluidic shell–core systems, cellulose nanocrystal (shell component) (CN)/methacrylated hyaluronic acid (HAMA) HIPE droplets were engineered for shear‐activated rupture under oxidant exposure, enabling on‐demand release only after combined chemical and mechanical stimuli, which operationally separates “printing stability” from “release activation” (X. Huang et al. 2023). An alternative strategy is to embed functionality in the printed porous lattice rather than the droplets. Enzymatic reactor polyHIPEs fabricated by extrusion‐based printing with simultaneous UV curing during deposition showed generally low enzyme leaching (∼0.14%–2.3% for most formulations), which was considered acceptable for immobilized‐enzyme reactor applications; however, leaching increased under specific printing conditions, particularly in fine 110‐µm‐nozzle‐printed structures, where mechanical deformation during washing raised enzyme loss to ∼8% and the estimated maximum activity error to 12.79% (Wenger et al. 2020). These mechanisms directly relate to how formulation affects nutrient transport and viability in cell‐relevant constructs (Section 4.4).

### 4.4 Influence of Formulation on Cell Viability and Proliferation

Cell compatibility in HIPPE‐derived constructs depends not only on the intrinsic biocompatibility of their components but also on how scaffold microstructure, porosity, interfacial chemistry, and potential leachable species influence cellular behavior under biological conditions (Ghosh et al. 2023; Y. Hu et al. 2019). Although HIPPE‐based printing has been extensively explored for food and nutraceutical applications, several HIPE/HIPPE‐derived systems have also been investigated for tissue‐engineering purposes. For instance, Ghosh et al. (2023) fabricated 3D‐printed poly(ε‐caprolactone) (PCL)‐based HIPE scaffolds exhibiting interconnected porous structures, high porosity (>93%), and support for human osteosarcoma MG‐63 cell line (MG63) osteoblast‐like cell growth. Similarly, T. Yang et al. (2017) reported printable PLLA/PCL HIPPE scaffolds with interconnected macroporous architectures that promoted cell attachment and proliferation, while Y. Hu et al. (2019) developed mesoporous hydroxyapatite (m‐HAp)/silica‐containing HIPPE scaffolds designed for bone tissue engineering applications. These studies demonstrate the potential of HIPPE‐derived architectures as tissue‐engineering platforms, although direct cell‐laden HIPPE bioprinting remains relatively underexplored.

Materials with solvent‐based continuous phases can still support cells after processing, yet they introduce formulation‐dependent toxicity limits. In PCL–dichloromethane (DCM)/water HIPE scaffolds, Cloisite 30B content improved stability (stable only when ≥1 wt%) but reduced MG63 viability when clay exceeded about 2 wt%, illustrating that the stabilizer level required for emulsion integrity can conflict with cellular tolerance even if the final scaffold is porous and mineralizable (Ghosh et al. 2023). In contrast, inorganic nanoparticles can be used in curing systems where washing and post‐curing reduce exposure; however, enzyme‐laden hydrogel‐in‐polyHIPE constructs still showed measurable enzyme leaching and diffusion limitations linked to void architecture and nozzle‐dependent equilibration times, demonstrating that pore connectivity and path length can be as biologically limiting as chemistry (Wenger et al. 2020).

Overall, current evidence suggests that HIPPE‐derived architectures can provide interconnected porosity, structural support, and compartmentalized cargo delivery while maintaining printability. However, most studies remain focused on scaffold fabrication rather than direct cell‐laden bioprinting, and systematic evaluation of long‐term cell behavior, tissue integration, and biological functionality remains limited. Consequently, biological validation currently lags behind advances in HIPPE formulation and printing design, highlighting the need for systematic investigations of long‐term cell viability, tissue integration, degradation behavior, and in vivo performance.

## 5 Application in Multidimensional Extrusion‐Based Printing

### 5.1 HIPPEs in 3D and 4D Bioprinting

In extrusion‐based 3D printing, HIPPEs serve as physically self‐supporting inks when the emulsion network simultaneously provides a measurable *τ*_0_ and fast thixotropic rebuild. This allows deposited filaments to “lock” before capillary leveling or gravity‐driven slump becomes dominant. This relationship is evident in systems where modest compositional changes shift dimensional error sharply. For example, in a recent study, κ‐carrageenan fractionation altered interfacial activity and droplet size (12.4 to 6.2 µm from κ‐carrageenan fraction [KCF] to κ‐carrageenan oligosaccharide fraction [KCO]) and increased elastic dominance (*G*ʹ ∼280 to 520 Pa), which reduced oil separation at 24 h (19% to 6%) and enabled high‐fidelity surimi structures with less than 3% dimensional error specifically for the strongest‐adsorbing fraction (Tyowua et al. 2024). A separate 3D route uses depletion‐driven droplet clustering rather than interfacial adsorption alone. OSA starch–citrus fiber HIPPEs printed with high shape accuracy (≈99.23% at 0.3% CF) while maintaining low‐friction tribology (friction coefficient [*μ*] < 0.05 at 10 mm/s), indicating that droplet packing together with fiber filling of the inter‐droplet spaces can produce both geometric stability and desirable oral‐processing mechanics in printed foods (F. Yang et al. 2024).

HIPPE‐enabled 4D printing is typically realized by embedding a stimulus‐responsive chemical potential into the continuous phase or by designing an interface that undergoes controllable weakening/rearrangement under defined triggers, while retaining sufficient mechanical robustness for printing at baseline conditions. Particles host–guest complexed by β‐CD provide a direct interfacial route. At oil 75%, tuning pH changed *τ*_0_ from ∼244 Pa (pH 3) to ∼34.8 Pa (pH 7) and improved recovery (3ITT) when β‐CD ≥1.2%, enabling printed lattices and complex motifs that later underwent programmed color change when curcumin (CUR) and NaHCO_3_ were incorporated (pH/thermal coupling) (X. Li, Fan, et al. 2023). A mechanistically distinct 4D pathway uses temperature‐triggered interfacial failure to drive pigment migration. β‐CD/CMC complexes maintained print integrity initially, but above ∼48°C–50°C, the complex weakened, producing rapid color transitions within ∼50–300 s at 60°C–65°C; the same design window also revealed the engineering constraint—severe oil leakage at 70°C—showing that 4D actuation speed can be increased at the cost of interfacial containment unless additional network reinforcement is introduced (Z. Guo et al. 2025).

Although the term multiresponsive printing is commonly associated with five‐axis manufacturing systems involving rotational motion, it is used here in an operational sense to describe spatially heterogeneous or functionally graded constructs in which location‐specific properties are programmed during extrusion through compositional or structural variation (Ghazal et al. 2023). Dual‐syringe deposition of KCF‐stabilized phases (KCF/κ‐carrageenan middle‐molecular‐weight fraction [KCM]/KCO) demonstrates this principle, because each fraction shifts droplet size and *G*ʹ in a predictable manner (12.4 → 6.2 µm; 280 → 520 Pa). Multiregion prints can be designed so that load‐bearing segments use KCO‐like rheology while compliant regions use KCF/KCM‐like rheology, without altering oil fraction or adding new crosslinkers mid‐print (Ghazal et al. 2023; Tyowua et al. 2024). Similarly, casein HIPEs tuned by phenolic‐rich shiitake oil produced large yield‐stress gains (81.8 → 309 Pa as shiitake increased) and a strong shift in plasticity, enabling prints only when the inter‐droplet network exceeded the threshold where cylinders no longer collapsed (best at 50%–60% shiitake oil blend). This creates a compositional axis for region‐specific stiffness within the same base formulation family (A.‐Q. Bi et al. 2024).

### 5.2 Emerging Multi‐Stimuli‐Responsive and Adaptive Architectures

**FIGURE 6:**
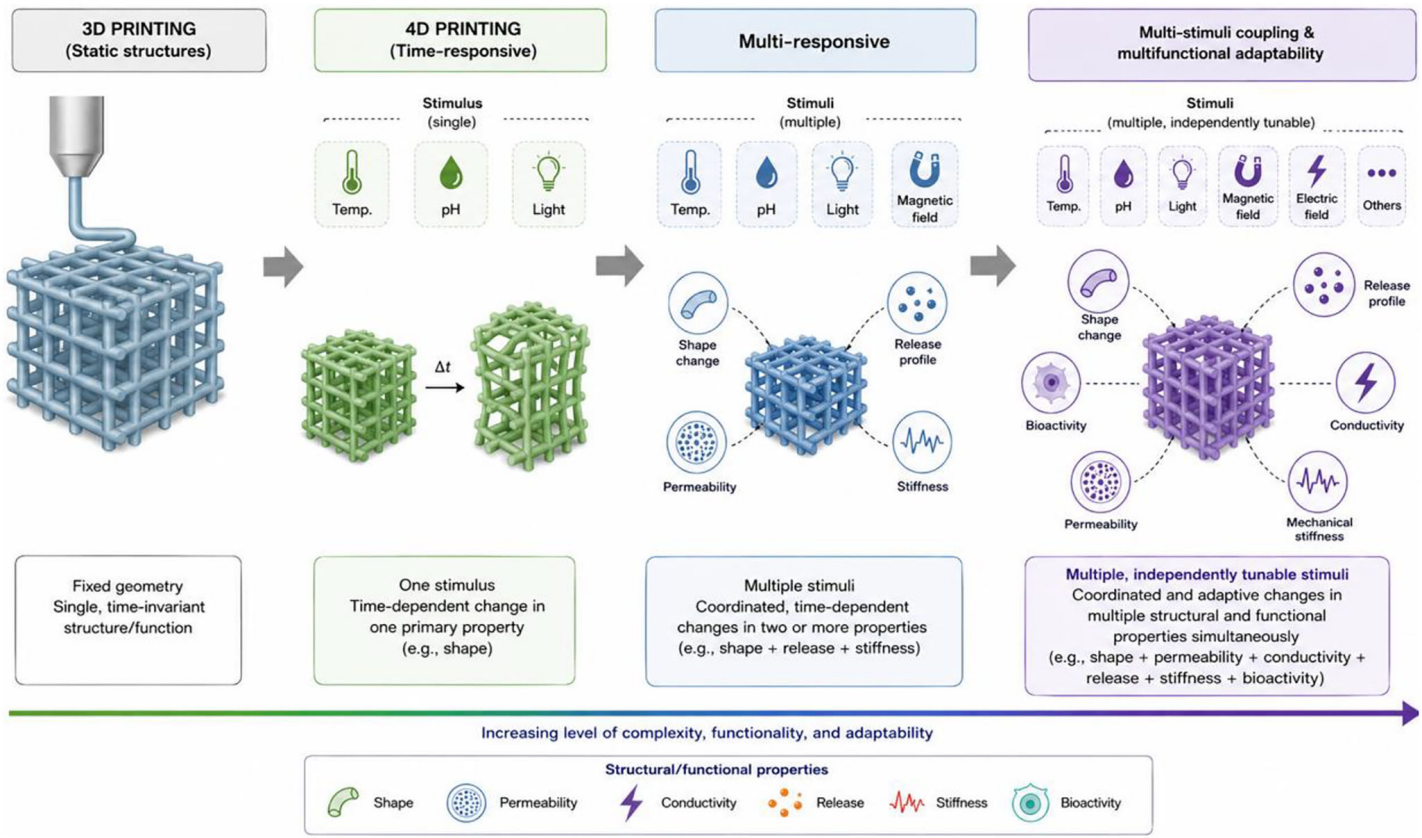
Schematic illustration of the progression from conventional 3D printing toward increasingly adaptive, multifunctional, and multiresponsive printing architectures.

Moving beyond conventional 4D concepts, emerging adaptive multiresponsive architectures involve multi‐stimuli coupling and multifunctional adaptability (Figure 6), where multiple independent stimuli trigger coordinated changes in more than one structural or functional property (e.g., shape, permeability, conductivity, release, or stiffness). Consequently, the progression illustrated in Figure 6 should be interpreted as increasing levels of functional adaptability and responsiveness rather than as distinct manufacturing dimensions. HIPPE architectures support this because stimulus sensitivity can be positioned at different hierarchical levels: (i) at the interface (particle shell), (ii) within the continuous matrix (polymer network), or (iii) within the dispersed phase (payload‐driven functions). Importantly, most HIPPE systems reported to date remain within the scope of 3D printing combined with single‐stimulus responsiveness and should therefore be considered enabling platforms for future adaptive multiresponsive architectures rather than fully realized multifunctional systems.

Quantitatively, multi‐stimuli programmability emerges when stimulus‐dependent modulus shifts exceed the mechanical safety margin required for structural retention. In β‐CD/CS HIPPEs (*φ* = 0.75), optimal formulations (β‐CD 1.2 wt%, CS 0.32 wt%, pH 5) exhibited strong *G*ʹ > *G*ʺ behavior and near‐complete thixotropic recovery, whereas at pH 7, the reduction in *ζ*‐potential and weakened interfacial membrane led to droplet coalescence and filament fusion (X. Li et al. 2023a). Likewise, thermoresponsive SNC‐P6 systems maintained long‐term stability (≥30 days) with lower critical solution temperature (LCST)‐like transitions near 37°C, allowing temperature‐triggered swelling without catastrophic droplet breakdown (Shahbazi, Jäger, et al. 2024). These data collectively imply that advanced multiresponsive functionality requires a dual condition: (i) stimulus‐induced modulation of interfacial or matrix cohesion and (ii) preservation of droplet‐scale confinement below the capillary failure threshold. Designing stimuli windows within this mechanical envelope is therefore essential to prevent leakage‐driven loss of dimensional accuracy.

Thermoresponsive Pickering stabilizers illustrate hierarchy‐level programming. Hydroxybutyl‐modified starch nanocrystals/nanoparticles (SNC‐P6/SNP‐P6) created long‐term stable HIPPE gels (≥30 days) with high print fidelity metrics (reported printability/print fidelity metric [Pr] ≈ 0.98 ± 0.08 for SNC‐based) and an LCST‐like transition around 37°C, enabling temperature‐triggered swelling behavior, which can be combined with geometric constraints from printing to yield temperature‐dependent actuation modes (Shahbazi, Jäger, et al. 2024). In metallic soft‐matter printing, liquid–metal high‐internal‐phase systems (eutectic gallium–indium alloy [EGaIn] 82.5%) stabilized by Carbopol–Ga^3^^+^ coordination exhibited very high elastic moduli (*G*ʹ ∼1.1 × 10^4^ → 4.1 × 10^4^ Pa) with shear thinning sufficient for DIW at ∼370–430 kPa, while remaining stable at 100°C for 24 h and supporting loads at −30°C. This couples mechanical stability, thermal resilience, and functional conductivity in a single printed architecture—an adaptive materials paradigm that is difficult to reach with conventional colloidal systems (Lin et al. 2024). Although no universally accepted definition of 6D printing currently exists, future HIPPE‐enabled 6D systems are expected to integrate multiple independently addressable stimuli capable of modulating several structural and functional properties simultaneously. For example, temperature‐responsive interfaces may regulate shape transformation, while pH‐responsive matrices simultaneously modulate release kinetics and permeability. Such hierarchical responsiveness could enable printed structures capable of adapting their geometry, transport properties, and functional performance according to environmental conditions. Realization of these systems will require precise integration of interfacial engineering, programmable materials, and digital manufacturing technologies. Current approaches toward 6D HIPPE architectures exhibit distinct advantages and limitations. Thermoresponsive systems offer simple actuation mechanisms and high printing fidelity but are often restricted by narrow temperature windows. pH‐responsive systems enable reversible modulation of structure and release behavior, although their performance may be sensitive to environmental fluctuations. Covalently anchored or dynamically crosslinked interfaces provide enhanced structural robustness and repeated‐stimulus durability but increase formulation complexity and manufacturing requirements. Conductive liquid–metal HIPPEs introduce electrical functionality and environmental adaptability; however, their applicability to food and biomedical systems remains limited. These observations suggest that future 6D HIPPE bioinks will likely require integration of multiple responsive mechanisms to simultaneously achieve printability, stability, adaptability, and application‐specific functionality.

### 5.3 Printed Constructs (Structure–Function Relationships)

Across HIPPE‐derived constructs, printed performance is often controlled more by interfacial packing and droplet‐scale organization than by bulk composition alone, because extrusion imposes large transient strains that can either preserve or disrupt the interfacial particle layer. Open‐cell porous scaffolds templated from printable HIPEs highlight this mapping from droplet architecture to macroporosity and transport. PLLA/PCL HIPEs stabilized by hydrophobic silica produced long‐term stable inks (at least 1 month) and yielded scaffolds with approximately 98.0%–98.3% porosity, where the internal water fraction controlled whether the final structure was open‐cell (*φ*_w_ ≥ 75%) or closed‐cell (*φ*_w_ ≤ 70%); this directly links printing feed formulation to post‐fabrication permeability and tissue‐ingrowth potential (T. Yang et al. 2017). In photopolymerizable emulsion systems, the same structure–function relationship is observed through processing optics rather than *φ* alone: adding tartrazine in stereolithography (SLA) polyMIPEs tuned pore features (surface pores ∼27.18 to 34.02 µm; interconnects ∼1.99 to 3.33 µm), altering porosity (∼63.9% down to ∼56% with tartrazine) while retaining print fidelity, which mechanistically reflects absorption‐controlled cure depth and hence emulsion “locking” kinetics during layer formation (Sengokmen‐Ozsoz et al. 2024).

### 5.4 Comparison With Conventional Hydrogel Bioinks

Compared with hydrogel inks, whose printability typically depends on polymer entanglement and rapid physical or chemical gelation, HIPPE inks achieve shape fidelity primarily through droplet jamming and interfacial elasticity, enabling high internal porosity and lipophilic payload compatibility without requiring very high polymer concentrations. A practical printing distinction is that HIPPE formulations can be tuned along orthogonal axes—interfacial layer strength (particle chemistry), packing/jamming (*φ* and droplet size), and matrix viscosity (continuous‐phase biopolymers)—so the same target fidelity can be reached with different trade‐offs in diffusion, softness, and stimulus response. Photocurable HIPE routes further decouple extrudability from final solidity. Polylactide glycidyl methacrylate (PLA‐GMA)/poly(ethylene glycol) diacrylate (PEGDA) HIPEs printed via digital light processing (DLP) achieved fast curing with controlled shrinkage (∼8%–10%) while retaining microporosity (pores ∼1–10 µm), indicating that emulsion templating can be embedded into light‐based additive manufacturing workflows to yield hierarchical porosity that homogeneous hydrogels do not readily generate at comparable build speeds (Choi et al. 2022). Compared with conventional hydrogel inks, HIPPEs often exhibit superior printing fidelity at comparable solid contents because droplet jamming and interfacial elasticity provide immediate post‐extrusion structural support. In addition, HIPPEs offer greater hydrophobic cargo LC, hierarchical porosity, and enhanced control over diffusion and mass transport through manipulation of droplet size, interfacial composition, and network connectivity. Conversely, hydrogel systems remain advantageous for cell‐laden bioprinting and tissue‐engineering applications because their cytocompatibility, degradation behavior, and cell–matrix interactions have been more extensively validated. Future bioink development may benefit from hybrid HIPPE–hydrogel platforms that combine the structural advantages of HIPPEs with the biological functionality of hydrogel systems. Recent reviews have highlighted the growing potential of advanced printing strategies for cultivated meat fabrication and other biofabrication applications, where precise control of rheology, hierarchical structure, and functionality is essential to successful construct development (Ng et al. 2025). Likewise, recent analyses of biopolymeric HIPPEs have emphasized that the same rheological characteristics governing food printability may facilitate future translation toward tissue‐engineering and regenerative‐medicine platforms (L. Zheng, Li, and Wang 2025). Beyond structural fabrication, these design advantages create opportunities for stimuli‐responsive and adaptive functionalities, which are discussed in the following section.

## 6 Stimuli‐Responsive and Functional Architectures

### 6.1 Stimuli Categories—Thermal, pH, Magnetic, Electric, and Biochemical Cues

Stimulus responsiveness in HIPPE‐based inks can be engineered at three interconnected levels, which are the continuous‐phase network, the particle‐laden interface, and the payload‐bearing internal phase. The same printed geometry can be programmed to change permeability, stiffness, or release kinetics under defined triggers. Thermally driven responses are frequently realized by thermoreversible or heat‐strengthened aqueous networks that preserve interfacial jamming while shifting modulus. A typical example is the Hofmeister‐strengthened gelatin HIPE, where Na_3_Cit‐induced chain bundling produced a strongly elastic, shear‐thinning system (reported *K*′ ≈ 1781.7 Pa; recovery >70%) and markedly improved thermal protection of an encapsulated polyphenol (resveratrol retention >80% after 8 h at 90°C) (L. Zheng, Li, Wang, et al. 2025). A second thermal axis is temperature‐sensitive interfacial integrity, where heat can intentionally weaken or reorganize the interface to enable functional switching; heat‐treated gelatin extracts showed that processing history shifts gel strength and print outcomes (e.g., gel strength ∼79.94 to 25.22 g across extraction regimes), demonstrating that “thermal stimulus” can be encoded as a controlled, formulation‐dependent change in network cohesion rather than only a post‐print trigger (Y. Wang, Zhang, et al. 2024).

The pH responsiveness is most effective when the trigger directly modulates electrostatic complexation and wettability, because both interfacial adsorption and bulk yield behavior shift concurrently. OP HIPPEs illustrate that viscosity and recovery depended strongly on pH, with the highest viscosity and near‐complete recovery reported around pH 3.5 (3ITT recovery ∼96.18% at optimized formulation), whereas higher pH levels caused weaker recovery and print defects such as layer fusion (Q.‐H. Li, Li, et al. 2023). For explicitly switchable behavior, dynamic covalent networks provide a sharper trigger. Aminated particles crosslinked with aldehyde hyaluronic acid formed printable emulsion‐filled gels whose emulsification/demulsification can be reversibly controlled by pH cycling (e.g., pH 8 → 2 → 8), linking pH to network continuity and therefore to release/transport states (Shi et al. 2025).

Magnetic and electric field‐responsive HIPPE systems remain comparatively underexplored, particularly in food‐grade and bioink applications. This limitation does not arise from difficulties in quantifying electromagnetic stimuli, since parameters such as field strength, frequency, magnetic flux density, and electrical input are routinely reported in broader additive‐manufacturing studies. Rather, the primary challenge lies in incorporating field‐responsive components into HIPPE formulations while simultaneously maintaining emulsion stability, printability, biocompatibility, or food‐grade compliance. Although electromagnetic‐field‐driven printing and actuation are well established in materials science, soft robotics, and biomedical engineering, their integration into HIPPE‐based printing remains at an early stage and represents a promising direction for future multifunctional and adaptive architectures.

Biochemical responsiveness differs from simple biofunctionalization because it requires a biological trigger to induce a structural or functional response. In principle, HIPPE architectures could achieve biochemical responsiveness through enzyme‐sensitive crosslinks, metabolite‐responsive interfaces, redox‐responsive particles, or biologically triggered degradation mechanisms that alter permeability, release behavior, or mechanical properties in response to specific biochemical signals. Although such strategies remain largely unexplored in HIPPE‐based printing, they represent an important future direction for adaptive biofabrication. By contrast, enzymatic reactor systems such as hydrogel‐filled polyHIPE constructs primarily demonstrate biofunctional activity rather than true biochemical responsiveness, since the enzyme performs a catalytic function without directly triggering adaptive structural changes in the printed architecture (Wenger et al. 2020).

### 6.2 Smart Materials and Responsive Behaviors Enabled by HIPPE‐Based Inks

HIPPE inks act as smart materials when a trigger changes one hierarchy level (interface, matrix, or internal phase) but is mechanically “amplified” by the jammed droplet skeleton into a macroscopic change in stiffness, permeability, or release. Oxidant–shear coupling is a clear example of orthogonal control; core–shell HIPE droplets with a CN/HAMA shell remain stable at rest but rupture rapidly under H_2_O_2_ (0.5%–1%) when combined with shear, enabling shear‐activated release only after chemical weakening of the shell (X. Huang et al. 2023). This differs structurally from pH‐only systems because chemical scission defines a rate‐limiting step at the shell, after which mechanical stress becomes the actuator—an architecture well‐aligned with “adaptive” constructs where function is gated by a local biochemical environment.

Light‐triggered strengthening provides a second route where the stimulus does not destabilize the emulsion but instead locks the microstructure for enhanced post‐print stability. UV‐dimerization of coumarin‐modified alginate particles increased viscosity (about two times higher reported post‐UV) and improved droplet packing (droplet size ∼16.8 µm after UV), converting a comparatively weaker pre‐UV ink into a more deformation‐resistant printed state without requiring bulk polymerization of the entire matrix (X. Zhao, Yang, et al. 2023). Mechanistically, this differs from thermal gelation because the stimulus primarily acts on particle–particle connectivity, thereby retaining printability, while the final object gains resistance to creep and shape relaxation.

A third design strategy involves small‐molecule modulation of freezing/phase transitions to preserve architecture during storage and thermal cycling—an important aspect of food‐grade “4D/6D‐like” performance defined by environmental history. Myofibrillar‐protein HIPEs with trehalose (optimal ∼10%) showed strong freeze–thaw resilience across multiple cycles (stable after F–T5 where controls failed early). This aligns with trehalose increasing interfacial adsorption (AP% reported to increase to ∼85.22% at 10% Tre) and reinforcing the jammed network against ice‐induced coalescence and water migration (Hong et al. 2025). This system illustrates an environmental stabilization strategy that improves the robustness and storage stability of printed HIPPE architectures under processing and storage stresses.

### 6.3 Emerging Trends in Multi‐Stimuli Adaptability and Hierarchical Design

Multi‐stimuli adaptability in HIPPE printing emerges through trigger stacking—where one stimulus adjusts interfacial or matrix state and another governs actuation. Practical examples include pH‐responsive rheology combined with temperature‐driven property shifts, as in OP HIPPEs that show strong pH‐dependent viscosity and contact‐angle changes with heating (reported *θ* increasing to about 90.2° at 50°C under optimized conditions), implying that pH can set the interfacial assembly state while temperature tunes droplet–matrix mobility during or after deposition (Q.‐H. Li, Li, et al. 2023). A complementary route uses environmental robustness (broad pH/salt tolerance) as a prerequisite for layered, heterogeneous constructs that experience local gradients. Hydrophobized CNC HIPPEs maintained stability across pH 3–12 and NaCl 0–1 M while preserving high thixotropic recovery (∼80%–90%), enabling printed objects that can be subsequently converted into a responsive colloidal system (e.g., methanol‐responsive or thermoresponsive variants) without catastrophic emulsion breakup—an enabling condition for hierarchical responsiveness rather than a single‐trigger effect (X. He et al. 2022).

## 7 Challenges, Limitations, and Knowledge Gaps

### 7.1 Material, Rheological, and Biocompatibility Challenges

A key limitation of HIPPE bioinks is that the same microstructural features that enable extrusion fidelity—high droplet crowding, percolated droplet contacts, and interfacial jamming—also compress the usable formulation space. This is evident in systems where stabilizer level simultaneously controls stability and biological compatibility; in a PCL–DCM/water HIPE, clay <0.5 wt% caused instability within <12 h, yet increasing Cloisite 30B above the stability threshold (≥1 wt%) introduced a separate constraint (>2 wt% reduced MG63 viability) illustrating how print‐stable interfacial coverage can conflict with cytocompatibility when inorganic particles are used (Ghosh et al. 2023). Even in food‐grade formulations, high structurant loadings can become a handling barrier. For instance, carboxylated chitin nanowhisker (C‐ChNW) HIPEs required approximately 7.5 wt% nanowhiskers to maintain *φ* ≈ 0.8 stability for 90 days, a level that markedly shifts the material toward a particulate gel where flow continuity and nozzle fouling become practical risks (Shuang et al. 2024).

Rheologically, many HIPPEs exhibit the “right” signatures (*τ*_0_, strong shear thinning, rapid recovery) but still fail in demanding geometries because the yield point is achieved by over‐consolidating the network. In gelatin oleofoam‐like systems, print fidelity improved only at higher gelatin concentrations, while low gelatin content leads to spreading and poor layer adhesion; yet the same gels become temperature‐fragile, melting above ∼30°C, which restricts processing and storage envelopes for structured foods and cell‐laden workflows (X. He and Lu 2024). Conversely, formulations tuned for very high viscosity or strong elasticity can impair extrudability and defect healing. More broadly, several HIPPE formulations exhibit favorable rheological indicators (high *τ*_0_, *G*ʹ > *G*ʺ, and rapid recovery) yet still fail in complex printing geometries due to excessive post‐extrusion stress relaxation, insufficient resistance to filament collapse, interlayer instability, or other nonlinear deformation phenomena that are not fully captured by conventional rheological measurements; for example, GA HIPEs showed the highest fidelity near 6 wt% GA, while at 8 wt%, it became excessively viscous with reduced flowability, demonstrating a practical upper bound on “stiffness‐driven” fidelity (Liang et al. 2024).

### 7.2 Scale‐Up, Reproducibility, and Regulatory Considerations

Reproducibility issues often arise because droplet size and interfacial packing depend on both formulation and energy history; scaling a rotor–stator or microfluidization recipe rarely preserves the same shear field distribution. This is evident in ambient‐curing W/O HIPE putty systems (ACC copolymer/solvent with zinc oxide [ZnO]), where a laboratory homogenization route (10,000 rpm, 2.5 min) was reported along with a scale‐up process (1500 rpm, 12 min). Although both produced printable HIPEs, the process is inherently history‐sensitive because curing proceeds over 3–7 days, and network consolidation is coupled to solvent evaporation and ionic/covalent crosslinking kinetics, amplifying batch‐to‐batch variation unless mixing and environmental conditions are tightly standardized (H. Jiang et al. 2024; Rashidi et al. 2022).

From a regulatory and translational perspective, solvent‐containing or reactive chemistries introduce additional hurdles beyond colloidal stability. Organic‐solvent HIPE scaffolds (e.g., DCM [CH_2_Cl_2_]‐containing systems) can deliver excellent porosity and printability but immediately raise concerns about residual solvents, occupational exposure, and material compatibility for food contact and biomedical use (Ghosh et al. 2023; Y. Hu et al. 2019). Even when materials are food‐grade, high‐energy processing (e.g., high‐pressure homogenization at hundreds of bars) complicates industrial adoption unless equipment equivalence, cleaning validation, and droplet‐size QC metrics are defined; in SPI microgel HIPEs, the combination of pre‐shear, HPH (500 bar for three cycles), and thermal treatment created strong networks and ensures high accuracy, but the multistep pathway increases critical control points that must be locked down for reproducible manufacturing (Wu et al. 2024).

For translational food applications, regulatory approval and food safety validation remain major barriers to industrial implementation. While many HIPPE formulations utilize generally recognized as safe (GRAS) proteins and polysaccharides, regulatory uncertainty may arise when chemically modified particles, nanostructured stabilizers, multifunctional additives, or solvent‐assisted fabrication routes are incorporated into printable food systems (Durgut and Claeyssens 2025; Rehman et al. 2024). In addition, high internal porosity and elevated water activity may increase susceptibility to lipid oxidation, microbial spoilage, and contamination during storage and distribution unless packaging strategies, hygienic processing conditions, preservation systems, and cold‐chain control are rigorously optimized (Tyowua et al. 2024). Standardized assessment of toxicological safety, residual solvent content, particle migration, allergenic potential, and storage stability, therefore, remains necessary before large‐scale commercialization of HIPPE‐based printed foods can be realistically achieved.

### 7.3 Gaps in Mechanistic Understanding and In Vivo Translation

A persistent knowledge gap is that most HIPPE printing studies report bulk rheology and static droplet sizes but rarely quantify dynamic interfacial mechanics under extrusion‐like deformation (fast area change, particle rearrangement, intermittent yielding). The consequence is that “same” *τ*_0_ values may not predict filament continuity or interlayer welding, especially when the interface is stimulus‐sensitive. For instance, β‐CD/CMC complex HIPEs showed abrupt weakening above ∼48°C–50°C with leakage at 70°C, implying that minor thermal fluctuation can shift the failure mode from bulk yielding to interfacial collapse; however, without interfacial rheology during heating–shearing, it remains difficult to model safe processing windows or predict failure thresholds across printers (Z. Guo et al. 2025).

Despite these promising scaffold‐based studies, the majority of HIPPE printing reports remain focused on food‐grade systems, encapsulation platforms, or acellular porous constructs. Direct bioprinting studies involving living cells, long‐term tissue maturation, vascularization, and in vivo performance remain scarce. Consequently, the designation of HIPPEs as bioinks currently reflects their potential applicability to biofabrication rather than widespread demonstration of cell‐laden bioprinting systems.

Translation to biomedical settings also remains limited by sparse long‐horizon biological evidence under realistic mechanical histories. Where cell data exist, it is often linked to a specific particle type or concentration window rather than to generalizable design rules. For example, clay‐stabilized polymeric HIPEs showed strong biomineralization capacity and drug‐loading performance, but cell viability became particle loading dependent, underscoring that “biocompatible outcome” cannot be inferred from print fidelity alone (Ghosh et al. 2023). Furthermore, the available biological evidence remains fragmented, with most studies relying on short‐term in vitro assessments using established cell lines rather than primary cells. Systematic investigations of long‐term cell proliferation, differentiation, tissue integration, immune response, and degradation‐related biological effects remain scarce, limiting the establishment of generalizable biocompatibility design criteria for HIPPE‐based bioinks. Systematic in vivo validation is uncommon relative to the breadth of in vitro print demonstrations, leaving degradation pathways, host–material responses, and clearance of particulate stabilizers insufficiently mapped for many candidate systems.

Another unresolved gap concerns the absence of standardized nonlinear rheological descriptors during extrusion‐relevant deformation. Although many studies report *G*ʹ, *G*ʺ, and *τ*_0_, fewer quantify large‐amplitude oscillatory shear (LAOS) behavior, strain‐stiffening, or harmonic distortion parameters that govern filament necking and interlayer welding. For example, SNC‐based HIPPEs exhibited reduced harmonic nonlinearity (*Q*_0_ decreased by ∼two orders of magnitude) relative to unmodified systems, correlating with improved scaffold integrity (Shahbazi, Jäger, et al. 2024). Similarly, soybean protein (SBP)‐NH_2_/oxidized tannic acid (OTA) nanonetwork HIPPEs achieved *G*ʹ increases from 84 to >1800 Pa with cinnamaldehyde (CA) bridging, but flow defects appeared at excessive crosslinking ratios (H. Chen, Yu, et al. 2025). These observations indicate that optimal printing does not coincide with maximal elasticity, but rather with a controlled balance between nonlinear strain accommodation and rapid structural rebuilding. Establishing LAOS‐based criteria or capillary number thresholds under extrusion flow would significantly enhance predictive capability and reproducibility across laboratories.

## 8 Future Perspectives

### 8.1 Design Strategies for Next‐Generation HIPPE‐Based Bioinks

Progress is likely to be driven by interface‐first design that decouples (i) extrusion flow, (ii) post‐deposition recovery, and (iii) long‐term stability, rather than using a single lever (more particles, higher polymer) to solve all three. One practical direction is to engineer high‐function, low‐solid interfaces by exploiting synergistic stabilizers that thicken the interfacial shell without forcing the continuous phase into a densely gelled state. Future research should focus on establishing quantitative design criteria linking interfacial coverage, particle morphology, droplet packing density, and rheological behavior to printing performance, thereby reducing reliance on empirical formulation optimization. In addition, future HIPPE bioinks should incorporate interfacial architectures capable of maintaining functionality under repeated stimulation cycles, since long‐term responsiveness is expected to depend not only on bulk rheology but also on the durability and adaptability of particle‐stabilized interfaces.

### 8.2 Integration With Artificial Intelligence and Digital Fabrication Paradigms

HIPPE formulation spaces are high‐dimensional (including oil fraction, particle chemistry, ionic strength, pH, processing energy, and aging), and therefore the output space is also multidimensional (such as flow curves, recovery kinetics, droplet statistics, and print metrics). This structure aligns well with data‐driven optimization, provided that the field converges on harmonized descriptors and test protocols. Future machine‐learning frameworks should enable predictive formulation design, optimization of printing parameters, and real‐time quality control through integration of rheological, interfacial, and printing‐performance datasets. Multi‐material and gradient printing will further benefit from such models because local composition shifts can be treated as controlled perturbations within a learned stability manifold, rather than as entirely new formulations each time.

### 8.3 Toward Multifunctional and Adaptive Biomedical Architectures

For biomedical translation, the most compelling trajectory is to leverage HIPPE compartmentalization to combine orthogonal functions—mechanical support, drug delivery, and controlled porosity—within a single printable ink, while maintaining low‐toxicity components and predictable degradation. Future biomedical applications should exploit HIPPE compartmentalization to simultaneously provide mechanical support, controlled release, interconnected porosity, and stimuli‐responsive functionality within a single printable platform. Particular attention should be directed toward long‐term biocompatibility, degradation behavior, in vivo validation, and the development of adaptive multi‐stimuli‐responsive architectures. Across these directions, the key enabling step is to consider interface mechanics, droplet topology, and processing history as equally important design factors—measured, modeled, and controlled—rather than optimized indirectly through trial‐and‐error formulation. A particularly important direction is the development of genuinely adaptive 6D architectures capable of responding to multiple independent stimuli while simultaneously modulating multiple properties such as shape, permeability, release behavior, conductivity, and mechanical performance. Achieving this objective will require hierarchical integration of responsive interfaces, programmable matrices, and functional payloads together with standardized criteria for evaluating multi‐stimuli responsiveness and long‐term functional stability. Such systems will likely require robust interfacial anchoring strategies capable of preserving structural integrity during repeated exposure to thermal, chemical, mechanical, or biochemical stimuli while maintaining predictable functional responses over extended operational periods.

## 9 Conclusion

HIPPEs have emerged as a distinctive class of interface‐engineered bioinks for extrusion‐based printing, combining high internal phase fractions (*φ* ≥ 0.74), tunable droplet architectures, and particle‐stabilized interfaces that generate the *τ*_0_, shear‐thinning behavior, and rapid structural recovery required for successful printing. Across the literature, successful HIPPE bioinks consistently operate within a common printability window characterized by droplet sizes generally below ∼15–20 µm, storage moduli typically exceeding ∼10^2^–10^3^ Pa, and structural recovery values above ∼80% in 3ITT measurements. Unlike conventional hydrogel inks that rely primarily on bulk polymer networks, HIPPEs derive their functionality from interfacial particle assembly and droplet jamming, enabling simultaneous control of printability, porosity, encapsulation, and stimuli‐responsive behavior. These attributes have supported applications ranging from food structuring and nutraceutical delivery to tissue‐engineering scaffolds and emerging adaptive multi‐stimuli‐responsive architectures responsive to thermal, pH, biochemical, and other environmental stimuli. Nevertheless, important challenges remain, including formulation sensitivity, limited understanding of dynamic interfacial behavior during extrusion, scale‐up reproducibility, long‐term biological validation, and food safety and regulatory considerations. Future progress will require standardized interfacial and rheological characterization, integration of artificial intelligence‐assisted formulation design, and comprehensive translational studies to establish HIPPEs as robust platforms for next‐generation multidimensional printing and multifunctional biofabrication.

## Author Contributions

**Parham Joolaei Ahranjani**: conceptualization, methodology, writing – original draft, investigation. **Kamine Dehghan**: conceptualization, investigation, methodology, writing – original draft. **Gergely Kali**: supervision, writing – review and editing. **Andreas Bernkop‐Schnürch**: supervision, writing – review and editing. **Giovanna Ferrentino**: supervision, writing – review and editing.

## Conflicts of Interest

The authors declare no conflicts of interest.

## Supporting information




**Supporting information**: crf370579‐sup‐0001‐SuppMat.docx
