# Supplementary material for: High Internal Phase Pickering Emulsions as Structurally Tunable Bioinks for Extrusion‐Based Printing From 3D Fabrication to Adaptive Multiresponsive Architectures
Source: Compr Rev Food Sci Food Saf. 2026 Jul 23;25(4):e70579. doi: 10.1111/1541-4337.70579 (PMC13396979; doi:10.1111/1541-4337.70579)
Supplement: Supplementary file 1 — Supporting information: crf370579‐sup‐0001‐SuppMat.docx [file CRF3-25-e70579-s001.docx]

**Supporting information**

**High internal phase Pickering emulsions as structurally tunable bio-inks for extrusion-based printing from 3D fabrication to adaptive multi-responsive architectures**

Parham Joolaei Ahranjani^1,2,†^, Kamine Dehghan^1,†^, Gergely Kali^2*^, Andreas Bernkop-Schnürch^2*^, Giovanna Ferrentino^1*^

^1^Faculty of Agricultural, Environmental and Food Sciences, Free University of Bolzano, Piazza Università, 1, Bolzano, 39100, Italy.

^2^Centre for Chemistry and Biomedicine (CCB), Department of Pharmaceutical Technology, Institute of Pharmacy, University of Innsbruck, Innrain 80/82, 6020 Innsbruck, Austria.

([Parhamjoolaei@gmail.com](mailto:Parhamjoolaei@gmail.com), [Kaminehdehghan@yahoo.com](mailto:Kaminehdehghan@yahoo.com))

^†^These authors contributed equally to this work.

(Corresponding authors: [giovanna.ferrentino@unibz.it](mailto:giovanna.ferrentino@unibz.it); [Gergely.Kali@uibk.ac.at](mailto:Gergely.Kali@uibk.ac.at); [andreas.bernkop@uibk.ac.at](mailto:andreas.bernkop@uibk.ac.at))

**Dec. 2025**

**Pub med: 142**

|  | **Search terms** | **Hits** |
| --- | --- | --- |
| **#1** | "High internal phase Pickering emulsions"[Title/Abstract] OR "High internal phase emulsions"[Title/Abstract] OR "Particle-stabilized HIPEs"[Title/Abstract] OR "Solid-stabilized high internal phase systems"[Title/Abstract] OR "High internal phase emulsion gels"[Title/Abstract] OR "Pickering emulsions"[Title/Abstract] OR "Solid particle-stabilized emulsions"[Title/Abstract] OR "emulsion gel"[Title/Abstract] OR "Irreversible interfacial adsorption"[Title/Abstract] OR "Colloidal particle stabilization"[Title/Abstract] OR "Wettability-controlled emulsions"[Title/Abstract] OR "Particle--liquid contact angle"[Title/Abstract] OR "Interfacial adsorption of particles"[Title/Abstract] OR "Biopolymer-stabilized emulsions"[Title/Abstract] OR "Natural particle emulsifiers"[Title/Abstract] OR "Emulsion interfacial engineering"[Title/Abstract] OR "Droplet stabilization by solids"[Title/Abstract] OR "Particle-jammed interfaces"[Title/Abstract] OR "Emulsion microstructure control"[Title/Abstract] OR "Food-grade Pickering systems"[Title/Abstract] OR "Protein--polysaccharide Pickering emulsions"[Title/Abstract] OR "pH- and ionic strength-responsive Pickering emulsions"[Title/Abstract] OR "Ultra-high internal phase emulsions"[Title/Abstract] OR "Pickering-stabilized concentrated emulsions"[Title/Abstract] OR "Interfacial particle-jammed networks"[Title/Abstract] OR "High oil volume fraction emulsions"[Title/Abstract] OR "Structured emulsions with particle interfaces"[Title/Abstract] OR "Viscoelastic HIPPE networks"[Title/Abstract] OR "τ₀ emulsion systems"[Title/Abstract] OR "Biopolymer-stabilized HIPPEs"[Title/Abstract] OR "Protein-polysaccharide HIPPEs"[Title/Abstract] OR "Irreversibly adsorbed particle interfaces"[Title/Abstract] | **3,031** |
| **#2** | "Extrusion-based printing"[Title/Abstract] OR "3D printing"[Title/Abstract] OR "4D printing"[Title/Abstract] OR "5D printing"[Title/Abstract] OR "6D printing"[Title/Abstract] OR "Multidimensional printing"[Title/Abstract] OR "Additive manufacturing of soft materials"[Title/Abstract] OR "Direct ink writing (DIW)"[Title/Abstract] OR "Rheology-driven printing"[Title/Abstract] OR "Viscoelastic ink extrusion"[Title/Abstract] OR "Multi-material printing"[Title/Abstract] OR "Stimuli-responsive printed structures"[Title/Abstract] OR "Shape-morphing architectures"[Title/Abstract] OR "Functional material fabrication"[Title/Abstract] OR "Structured food fabrication"[Title/Abstract] OR "Food-grade additive manufacturing"[Title/Abstract] OR "Layer-by-layer deposition"[Title/Abstract] OR "Customized printing architectures"[Title/Abstract] OR "Dynamic printing platforms"[Title/Abstract] OR "High-fidelity extrusion printing"[Title/Abstract] | **26,570** |
| **#3** | #1 AND #2 | **142** |

**Scopus: 312**

|  | **Search terms** | **Hits** |
| --- | --- | --- |
| **#1** | TITLE-ABS-KEY ("High internal phase Pickering emulsions" OR "High internal phase emulsions" OR "Particle-stabilized HIPEs" OR "Solid-stabilized high internal phase systems" OR "High internal phase emulsion gels" OR "Pickering emulsions" OR "Solid particle-stabilized emulsions" OR "emul-gel" OR "Irreversible interfacial adsorption" OR "Colloidal particle stabilization" OR "Wettability-controlled emulsions" OR "Particle-liquid contact angle" OR "Interfacial adsorption of particles" OR "Biopolymer-stabilized emulsions" OR "Natural particle emulsifiers" OR "Emulsion interfacial engineering" OR "Droplet stabilization by solids" OR "Particle-jammed interfaces" OR "Emulsion microstructure control" OR "Food-grade Pickering systems" OR "Protein--polysaccharide Pickering emulsions" OR "pH- and ionic strength-responsive Pickering emulsions" OR "Ultra-high internal phase emulsions" OR "Pickering-stabilized concentrated emulsions" OR "Interfacial particle-jammed networks" OR "High oil volume fraction emulsions" OR "Structured emulsions with particle interfaces" OR "Viscoelastic HIPPE networks" OR "τ₀ emulsion systems" OR "Biopolymer-stabilized HIPPEs" OR "Protein-polysaccharide HIPPEs" OR "Irreversibly adsorbed particle interfaces" ) | **8,992** |
| **#2** | TITLE-ABS-KEY ( "Extrusion-based printing" OR "3D printing" OR "4D printing" OR "5D printing" OR "6D printing" OR "Multidimensional printing" OR "Additive manufacturing of soft materials" OR "Direct ink writing (DIW)" OR "Rheology-driven printing" OR "Viscoelastic ink extrusion" OR "Multi-material printing" OR "Stimuli-responsive printed structures" OR "Shape-morphing architectures" OR "Functional material fabrication" OR "Structured food fabrication" OR "Food-grade additive manufacturing" OR "Layer-by-layer deposition" OR "Customized printing architectures" OR "Dynamic printing platforms" OR "High-fidelity extrusion printing") | **103,805** |
| **#3** | #1 AND #2 | **312** |

**Web of science: 214**

|  | **Search terms** | **Hits** |
| --- | --- | --- |
| **#1** | **TI=**("High internal phase Pickering emulsions" OR "High internal phase emulsions" OR "Particle-stabilized HIPEs" OR "Solid-stabilized high internal phase systems" OR "High internal phase emulsion gels" OR "Pickering emulsions" OR "Solid particle-stabilized emulsions" OR "emul-gel" OR "Irreversible interfacial adsorption" OR "Colloidal particle stabilization" OR "Wettability-controlled emulsions" OR "Particle--liquid contact angle" OR "Interfacial adsorption of particles" OR "Biopolymer-stabilized emulsions" OR "Natural particle emulsifiers" OR "Emulsion interfacial engineering" OR "Droplet stabilization by solids" OR "Particle-jammed interfaces" OR "Emulsion microstructure control" OR "Food-grade Pickering systems" OR "Protein--polysaccharide Pickering emulsions" OR "pH- and ionic strength-responsive Pickering emulsions" OR "Ultra-high internal phase emulsions" OR "Pickering-stabilized concentrated emulsions" OR "Interfacial particle-jammed networks" OR "High oil volume fraction emulsions" OR "Structured emulsions with particle interfaces" OR "Viscoelastic HIPPE networks" OR "τ₀ emulsion systems" OR "Biopolymer-stabilized HIPPEs" OR "Protein-polysaccharide HIPPEs" OR "Irreversibly adsorbed particle interfaces") | **2,569** |
| **#2** | **AB=**("High internal phase Pickering emulsions" OR "High internal phase emulsions" OR "Particle-stabilized HIPEs" OR "Solid-stabilized high internal phase systems" OR "High internal phase emulsion gels" OR "Pickering emulsions" OR "Solid particle-stabilized emulsions" OR "emul-gel" OR "Irreversible interfacial adsorption" OR "Colloidal particle stabilization" OR "Wettability-controlled emulsions" OR "Particle--liquid contact angle" OR "Interfacial adsorption of particles" OR "Biopolymer-stabilized emulsions" OR "Natural particle emulsifiers" OR "Emulsion interfacial engineering" OR "Droplet stabilization by solids" OR "Particle-jammed interfaces" OR "Emulsion microstructure control" OR "Food-grade Pickering systems" OR "Protein--polysaccharide Pickering emulsions" OR "pH- and ionic strength-responsive Pickering emulsions" OR "Ultra-high internal phase emulsions" OR "Pickering-stabilized concentrated emulsions" OR "Interfacial particle-jammed networks" OR "High oil volume fraction emulsions" OR "Structured emulsions with particle interfaces" OR "Viscoelastic HIPPE networks" OR "τ₀ emulsion systems" OR "Biopolymer-stabilized HIPPEs" OR "Protein-polysaccharide HIPPEs" OR "Irreversibly adsorbed particle interfaces") | **4,417** |
| **#3** | **AK=**("High internal phase Pickering emulsions" OR "High internal phase emulsions" OR "Particle-stabilized HIPEs" OR "Solid-stabilized high internal phase systems" OR "High internal phase emulsion gels" OR "Pickering emulsions" OR "Solid particle-stabilized emulsions" OR "emul-gel" OR "Irreversible interfacial adsorption" OR "Colloidal particle stabilization" OR "Wettability-controlled emulsions" OR "Particle--liquid contact angle" OR "Interfacial adsorption of particles" OR "Biopolymer-stabilized emulsions" OR "Natural particle emulsifiers" OR "Emulsion interfacial engineering" OR "Droplet stabilization by solids" OR "Particle-jammed interfaces" OR "Emulsion microstructure control" OR "Food-grade Pickering systems" OR "Protein--polysaccharide Pickering emulsions" OR "pH- and ionic strength-responsive Pickering emulsions" OR "Ultra-high internal phase emulsions" OR "Pickering-stabilized concentrated emulsions" OR "Interfacial particle-jammed networks" OR "High oil volume fraction emulsions" OR "Structured emulsions with particle interfaces" OR "Viscoelastic HIPPE networks" OR "τ₀ emulsion systems" OR "Biopolymer-stabilized HIPPEs" OR "Protein-polysaccharide HIPPEs" OR "Irreversibly adsorbed particle interfaces") | **1,411** |
| **#4** | **TI=(** "Extrusion-based printing" OR "3D printing" OR "4D printing" OR "5D printing" OR "6D printing" OR "Multidimensional printing" OR "Additive manufacturing of soft materials" OR "Direct ink writing (DIW)" OR "Rheology-driven printing" OR "Viscoelastic ink extrusion" OR "Multi-material printing" OR "Stimuli-responsive printed structures" OR "Shape-morphing architectures" OR "Functional material fabrication" OR "Structured food fabrication" OR "Food-grade additive manufacturing" OR "Layer-by-layer deposition" OR "Customized printing architectures" OR "Dynamic printing platforms" OR "High-fidelity extrusion printing"**)** | **19,976** |
| **#5** | **AB=(** "Extrusion-based printing" OR "3D printing" OR "4D printing" OR "5D printing" OR "6D printing" OR "Multidimensional printing" OR "Additive manufacturing of soft materials" OR "Direct ink writing (DIW)" OR "Rheology-driven printing" OR "Viscoelastic ink extrusion" OR "Multi-material printing" OR "Stimuli-responsive printed structures" OR "Shape-morphing architectures" OR "Functional material fabrication" OR "Structured food fabrication" OR "Food-grade additive manufacturing" OR "Layer-by-layer deposition" OR "Customized printing architectures" OR "Dynamic printing platforms" OR "High-fidelity extrusion printing"**)** | **51,530** |
| **#6** | **AK=(** "Extrusion-based printing" OR "3D printing" OR "4D printing" OR "5D printing" OR "6D printing" OR "Multidimensional printing" OR "Additive manufacturing of soft materials" OR "Direct ink writing (DIW)" OR "Rheology-driven printing" OR "Viscoelastic ink extrusion" OR "Multi-material printing" OR "Stimuli-responsive printed structures" OR "Shape-morphing architectures" OR "Functional material fabrication" OR "Structured food fabrication" OR "Food-grade additive manufacturing" OR "Layer-by-layer deposition" OR "Customized printing architectures" OR "Dynamic printing platforms" OR "High-fidelity extrusion printing"**)** | **39,767** |
| **#7** | #1 OR #2 OR #3 | **5,324** |
| **#8** | #4 OR #5 OR #6 | **71,656** |
| **#9** | #7 AND #8 | **214** |

Total references: 668

Unrepeated references: 352

Relevant references: 143
